# Synthesis and Neuroprotective Action of Xyloketal Derivatives in Parkinson’s Disease Models

**DOI:** 10.3390/md11125159

**Published:** 2013-12-18

**Authors:** Shichang Li, Cunzhou Shen, Wenyuan Guo, Xuefei Zhang, Shixin Liu, Fengyin Liang, Zhongliang Xu, Zhong Pei, Huacan Song, Liqin Qiu, Yongcheng Lin, Jiyan Pang

**Affiliations:** 1 School of Chemistry & Chemical Engineering, Sun Yat-Sen University, No. 135 Xingangxi Road, Guangzhou 510275, China; E-Mails: lishich@mail2.sysu.edu.cn (S.L.); zhxuefei@mail2.sysu.edu.cn (X.Z.); lsxinhoho@gmail.com (S.L.); xzhliang1979@163.com (Z.X.); qiuliqin@mail.sysu.edu.cn (L.Q.); ceslyc@mail.sysu.edu.cn (Y.L.); 2 Department of Neurology, The First Affiliated Hospital, Sun Yat-Sen University, No. 58 Zhongshan Road II, Guangzhou 510080, China; E-Mails: sczmc39@sina.com (C.S.); guoweny@mail2.sysu.edu.cn (W.G.); liangfy@mail2.sysu.edu.cn (F.L.); peizhong@yahoo.com (Z.P.)

**Keywords:** xyloketal, *Caenorhabditis elegans*, zebrafish, *C57BL/6*, Parkinson’s disease

## Abstract

Parkinson’s disease (PD) is the second most common neurodegenerative disease affecting people over age 55. Oxidative stress actively participates in the dopaminergic (DA) neuron degeneration of PD. Xyloketals are a series of natural compounds from marine mangrove fungus strain *No. 2508* that have been reported to protect against neurotoxicity through their antioxidant properties. However, their protection *versus* 1-methyl-4-phenylpyridinium (MPP+)-induced neurotoxicity is only modest, and appropriate structural modifications are necessary to discover better candidates for treating PD. In this work, we designed and synthesized 39 novel xyloketal derivatives (**1**–**39**) in addition to the previously reported compound, xyloketal B. The neuroprotective activities of all 40 compounds were evaluated *in vivo* via respiratory burst assays and longevity-extending assays. During the zebrafish respiratory burst assay, compounds **1**, **9**, **23**, **24**, **36** and **39** strongly attenuated reactive oxygen species (ROS) generation at 50 μM. In the *Caenorhabditis elegans* longevity-extending assay, compounds **1**, **8**, **15**, **16** and **36** significantly extended the survival rates (*p* < 0.005 *vs.* dimethyl sulfoxide (DMSO)). A total of 15 compounds were tested for the treatment of Parkinson’s disease using the MPP+-induced *C. elegans* model, and compounds **1** and **8** exhibited the highest activities (*p* < 0.005 *vs.* MPP^+^). In the MPP+-induced *C57BL/6* mouse PD model, 40 mg/kg of **1** and **8** protected against MPP+-induced dopaminergic neurodegeneration and increased the number of DA neurons from 53% for the MPP+ group to 78% and 74%, respectively (*p* < 0.001 *vs.* MPP+ group). Thus, these derivatives are novel candidates for the treatment of PD.

## 1. Introduction

Parkinson’s disease (PD), the second most common neurodegenerative disease, affects a large number of people and has a mean onset age of 55, and the incidence increases with age. Substantial evidence has demonstrated that mitochondrial dysfunction and the consequent oxidative stress, including oxidation of dopamine species, initially provokes this neurodegeneration [1,2]. Oxidative stress can damage lipids, proteins and DNA [3,4] and induce Lewy Body (LB) protein aggregation [5]. Therefore, blocking oxidative stress is one strategy for treating PD. Coenzyme Q10 (CoQ10) is a natural antioxidant that has been isolated and assessed for its effect on PD [6,7]. Many of the neurotoxins involved in PD, such as 6-hydroxydopamine (6-OHDA), 1-methyl-4-phenyl-1,2,3,6-tetrahydropyridine (MPTP), paraquat and rotenone [8,9,10,11], induce neurodegeneration via the formation of reactive oxygen species (ROS). Of these compounds, MPTP, which is metabolized to the active 1-methyl-4-phenylpyridinium (MPP+), has been widely used in toxin-induced PD models, because it can induce parkinsonian syndrome with all of the characteristic features of PD [12,13,14].

Model organisms, such as zebrafish, *C. elegans*, yeast and *drosophila melanogaster*, are powerful tools in drug screening and signaling research [15] because of their short lifecycles, availability and low costs. More importantly, using whole organisms provides more insight into the biological processes of mammalian diseases than *in vitro* models. Zebrafish respiratory burst assays are an ROS assay based on the activity of NADPH oxidase. Juglone can produce ROS that induce mitochondrial dysfunction and cause mitochondrial membrane potential loss during *C. elegans* longevity-extending assays [16,17]. These two assays are often used for the high-throughput screening of compounds and the *in vivo* identification of therapeutic targets [18,19].

**Chart 1:**
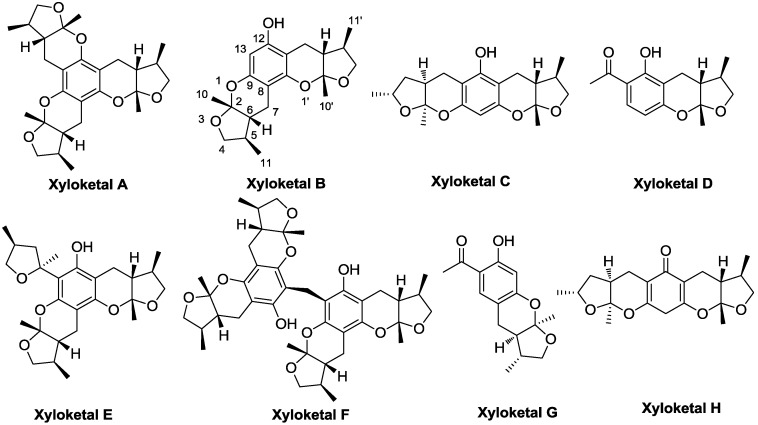
Structure of naturally isolated xyloketals A–H.

**Chart 2:**
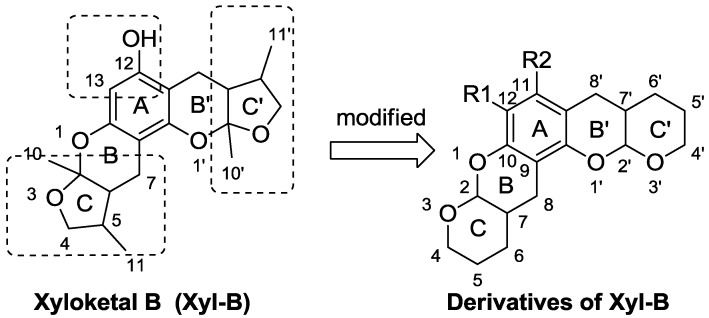
Modifications of xyloketal B.

Marine environments are rich sources of novel and unusual secondary metabolites, many of which show considerable promise as therapeutic agents. However, most of these compounds are stereochemically complex or have low activity. To develop chemically simple and active candidates, appropriate structural modifications are important. Xyloketals are a series of novel natural compounds from marine mangrove fungi (Chart 1) [20,21,22,23,24]. We previously demonstrated that xyloketal B showed protection in different cell models and can protect against MPP+-induced neurotoxicity via its antioxidant properties [25,26,27,28]. Considering their remarkable structural and biological properties, the stereoselective synthesis of xyloketals has attracted much interest because of their unique bicyclic acetal moieties fused to their aromatic core structure [29,30,31,32,33,34,35,36,37]. Although xyloketal B displays good *in vitro* antioxidative activity, its protective action against neural cell injury is moderate, and its effects in PD treatment remain unproven. Moreover, the structure of xyloketal B (Chart 1), which contains six asymmetric carbons at the 2, 2′, 5, 5′, 6 and 6′ positions, renders its asymmetric synthesis and modification difficult. Therefore, we have become keenly interested in designing and synthesizing novel analogues of xyloketal B to overcome its shortcomings, improve its activity and discover potential candidates for treating PD. In this paper, we describe the design, synthesis, biological evaluation using multiple models and structure-activity relationship (SAR) of 39 new xyloketal derivatives (**1**–**39**) and the previously reported xyloketal B (Xyl-B) [37]. The modifications to xyloketal B focus on expanding the tetrahydrofuran ring into a tetrahydropyran ring and adding substituents to the 12- and 13-positions of the aromatic ring (Chart 2). The antioxidant activities of all 40 compounds were evaluated in oxidative models using zebrafish [38] and *C. elegans* [17]. The neuroprotective activities of any compound with high antioxidative actions were further investigated using MPP+-induced PD models [19,27], including the *C. elegans* and *C57BL/6* mouse models [39], to evaluate their ability to protect against dopaminergic neuron degeneration.

## 2. Results and Discussion

### 2.1. Chemistry

**Scheme 1:**
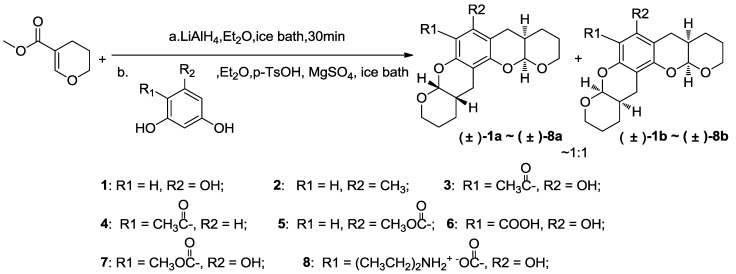
Synthesis of compounds **1**–**8**.

**Scheme 2:**
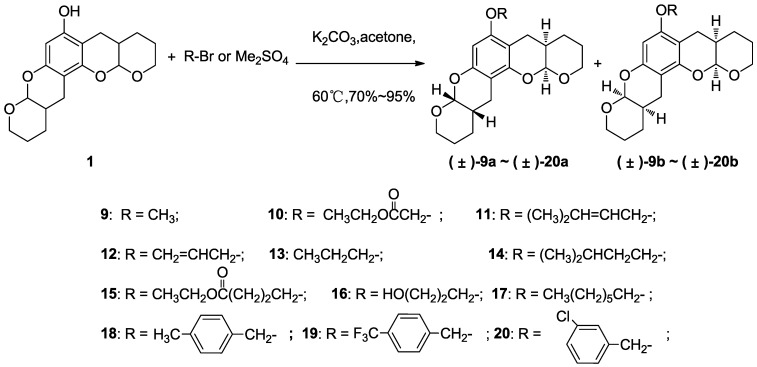
Etherification of **1** using different alkylating agents to prepare **9**–**20**.

**Scheme 3:**
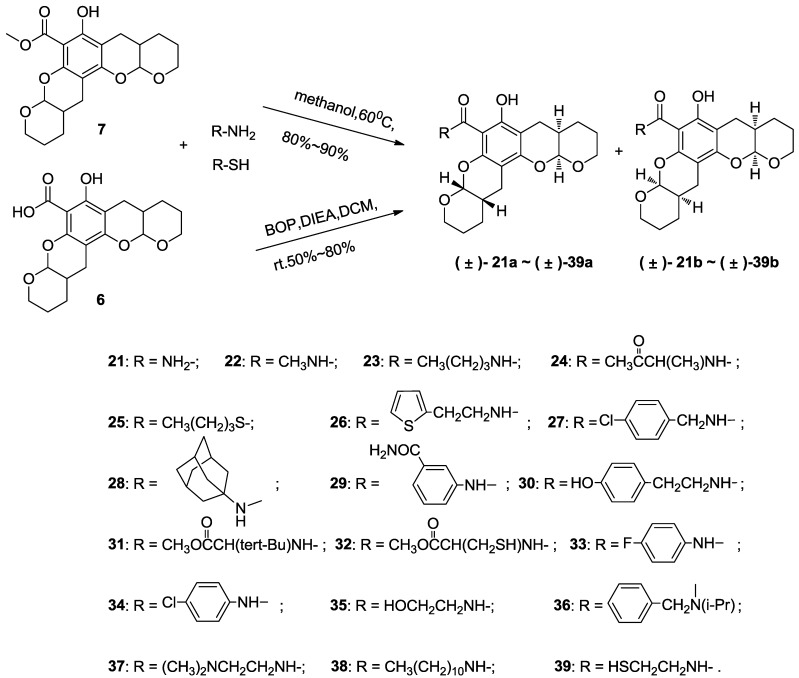
Compounds**21**–**39** are the amide and carbonyl sulfide products of **6**.

The synthetic routes to **1**–**39** are shown in Scheme 1, Scheme 2, Scheme 3, and all new compounds were fully characterized by MS and NMR. The syntheses were performed in the ordinary state without any asymmetric factors, and compounds **1**–**8** were obtained as stereoisomeric mixtures, including both the diastereoisomers and enantiomers from (±)-**1a** and (±)-**1b** to (±)-**8a** and (±)-**8b** by coupling different phenols with the reduction product of methyl 3,4-dihydro-2*H*-pyran-5-carboxylate using previously reported conditions [33,34,37], as shown in Scheme 1. The core intermediate, methyl 3,4-dihydro-2*H*-pyran-5-carboxylate, was easily prepared from commercially available 2,3-dihydropyran [40]. This ester was reduced with lithium aluminum hydride in anhydrous diethyl ether to yield the corresponding alcohol and immediately subjected to a *p*-toluene sulfonic acid-promoted electrophilic aromatic substitution with various phenols to afford targets **1**–**8** in yields of 55%–90%, depending on the phenol.

These compounds were isolated as bis-adduct analogues to Xyl-B and were easily characterized as angular via NMR spectroscopy, which is consistent with the authentic natural product and previous synthetic work, because linear compounds can convert into the more stable angular molecules [21,33]. The two oxygen-containing pyran rings, B and C, can be connected in a *cis* or *trans* fashion. Previous studies indicate that rings B and C were *cis* in the natural and synthetic compounds for all condensations, leading to xyloketal derivatives [28,29,30,31,32,33,34,35,36]. Therefore, two sets of xyloketal derivative diastereoisomers can be formed.

**Chart 3:**
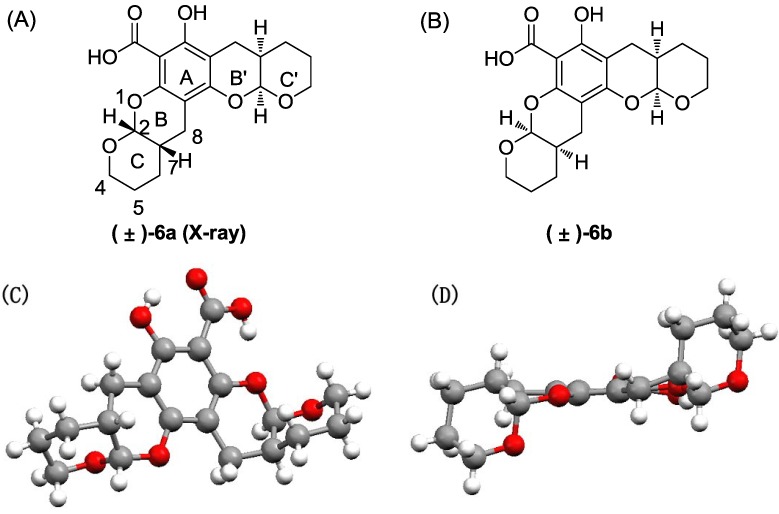
The structures for (±)-**6a** (**A**) and (±)-**6b** (**B**); X-ray structure for one isomer of (±)-**6a** from the front (**C**) and side (**D**) views.

Separating the diastereoisomers via chromatography was very difficult, because of their similarity. In the HPLC analysis, most of the final products showed one or two close peaks under the experimental conditions. The LC-MS results gave the same molecular weight for these two peaks, which suggests that the compounds were diastereomeric mixtures (see the Supplementary Information). However, we found that the diastereoisomers of compound **6** could be separated through repeated chromatography and crystallization. Thus, product **6** was analyzed as an example. Compound **6** was purified via flash chromatography followed by crystallization to afford two pure ingredients, (±)**-6a** and (±)-**6b** (Chart 3). A single (±)**-6a** crystal was used for X-ray crystal analysis (Chart 3A). This analysis confirmed that (±)**-6a** was isolated as a mixture of an enantiomers pair. The crystal structure indicated that the rings were angularly arranged similar to the natural product, xyloketal B; the orientation of rings C and C′ was the *anti-type*, and H-2 and H-7, as well as H-2′and H-7′ were *cis* (the NMR and X-ray data for compound **6** is provided in the Supplementary Information.). Most signals in the 1D and 2D NMR spectra for (±)-**6b** and (±)-**6a** were similar. In the ^1^H NMR spectrum for (±)-**6b**, the coupling constants between H-2 and H-7 and between H-2′and H-7′ were 2.4 and 2.8 Hz, respectively, which indicates a *syn* relationship, as in (±)-**6a**. However, some changes were observed in the ^13^C NMR spectra, especially in the C-4 and C-8 shifts in (±)-**6a** and (±)-**6b,** which differed by δ_C_ 0.41 and 0.37, respectively. Finally, (±)-**6b** was established as the diastereoisomer of (±)-**6a** via NMR analysis. The methenyl hydrogen atoms (H-2, 2′) in the ^1^H NMR spectra of the mixtures were doublets of doublets with chemical shifts of 5.53 and 5.37 for (±)-**6a** and 5.52 and 5.35 for (±)-**6b**. The integral of the H-2 and 2′ peaks in the mixture of (±)-**6a** and (±)-**6b** showed that the diastereoisomeric ratio was ~1:1. Moreover, the integral ratio of the carbon atoms (C-7, 7′) in the mixtures of (±)-**6a** (δ = 30.89, 30.66) and (±)-**6b** (δ = 30.81, 30.75) was ~1:1, which supported the diastereoisomeric ratio (1:1) found for **6**. Although we separated small amounts of diastereoisomers of compound **6**, we performed all further modifications and biological evaluations using the mixture of **6** and its diastereoisomers; thus, the activity of **6** could be compared with those of the other compounds.

The NMR spectra for **1**–**8** show diastereoisomeric features similar to those of **6**. For example, both the ^1^H and ^13^C NMR spectra of **1** show evidence of the diastereoisomers, (±)-**1a** and (±)-**1b**, which have overlapping peaks. The aromatic carbon atom (C-12) resonates as a single peak, and the methenyl (C-2, C-2′) and methylene groups (C-4, C-4′) both present as four closely packed peaks. Moreover, the methenyl groups (C-7, 7′) also appear as four peaks (δ *=* 31.24, 31.20, 31.11 and 31.08). These peaks all indicate that compound **1** consists of two sets of diastereoisomers. Comparing the carbon atoms (C-7, 7′) in (±)-**6a** and (±)-**6b** to those in **1** indicates that the peaks at δ = 31.24 and 31.08 and those at δ = 31.20 and 31.11 belong to diastereoisomers (±)-**1a** and (±)-**1b**, respectively. The integral of the carbon atoms, C-7 and C-7′, indicates an approximately 1:1 ratio of diastereoisomers (±)-**1a** and (±)-**1b**. The structures of compounds **2**–**8** were determined using the same methods used for **1**. 

Compounds **9**–**20** (Scheme 2) were produced in high yield via etherification of the hydroxyl groups on **1** using dimethyl sulfate or various alkyl bromides in the presence of potassium carbonate [37]. The etherification reactions of **1** did not involve the chiral carbons, and we reasoned that the stereochemistry of these compounds remained the same as those for the precursor, **1**. Therefore, two sets of diastereoisomers were likely formed for each derivative in a ~1:1 ratio. This assumption was supported by the NMR data.

The synthetic route to **21**–**39** is shown in Scheme 3. The target compounds were also obtained as mixtures of stereoisomers from (±)-**21a** and (±)-**21b** to (±)-**39a** and (±)-**39b**, which were synthesized by coupling **6** with the corresponding amine or thiol under moderate reaction conditions in the presence of (benzotriazol-1-yloxy)tris(dimethylamino)phosphonium hexafluorophosphate (BOP) and *N*,*N*-diisopropylethylamine (DIEA) or by the amination of **7** [37]. Compound **8** is the diethylamine salt of **6** and shows improved water solubility. The NMR spectra for **21**–**39** indicate diastereoisomeric features similar to those of **6**. The coupling reactions resulted in the desired compounds without affecting the chiral carbons; thus, the amide and carbonyl sulfide products (**21**–**39**) should retain the stereochemistry of **6**.

### 2.2. Xyloketal Derivatives Attenuate the PMA-Induced Neurotoxicity in a Zebrafish Respiratory Burst Assay

The antioxidant activity of the xyloketal derivatives were examined via a zebrafish respiratory burst assay. In these zebrafish respiratory burst assays, phorbol myristate acetate (PMA) was used to activate NADPH oxidase and to induce the generation of excessive quantities of ROS; therefore, a nonfluorescent dye, such as 2′,7′-dihydrodichlorofluorescein diacetate (H_2_DCFDA), could be used to detect the ROS formation based on its oxidization to fluorescent dichlorofluorescein (DCF).

Separating the diastereoisomers was very difficult; therefore, all 39 xyloketal derivatives were used directly in the biological screenings without separating the diastereoisomers. These compounds were obtained through the same synthetic methods and possessed the same structural framework and ring fusion stereochemistry; the only differences were the benzene ring substituents. Although the test compounds are enantiomeric and diastereomeric mixtures, their activity and a preliminary SAR analysis was obtained.

**Figure 1:**
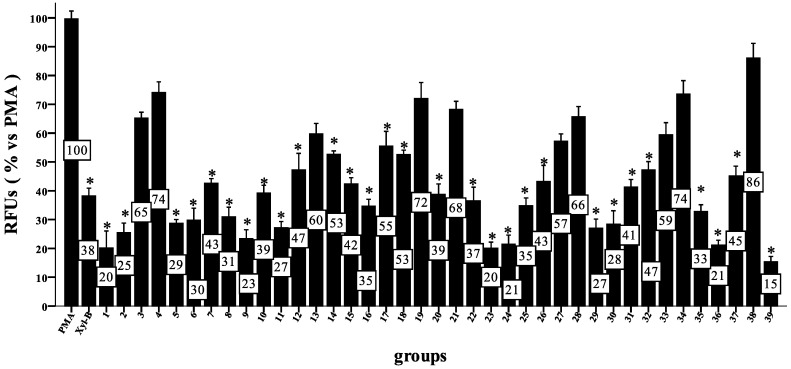
Effect of compounds on phorbol myristate acetate (PMA)-induced reactive oxygen species (ROS) in a respiratory burst assay. Zebrafish were pretreated 72 h post-fertilization (hpf) with one of the compounds or dimethyl sulfoxide (DMSO) for 30 min and then incubated with PMA and 2′,7′-dihydrodichlorofluorescein diacetate (H_2_DCFDA) for 150 min at 28 °C. Relative fluorescence units (RFUs) were measured using a fluorescent instrument (ex. of 485 nm and em. of 530 nm). The RFUs of the PMA group were set to 100%, and the other groups are shown relative to this group. Data were processed via a one-way ANOVA followed by a post hoc test (Games Howell method) (* *p* < 0.01 *vs.* PMA, *n* = 6 wells for each group). Three independent experiments were performed.

The results (Figure 1) showed that most of the compounds exhibited strong antioxidative activity (*p* < 0.01 *vs.* PMA). Compounds **1**, **9**, **23**, **24**, **36** and **39** reduced the ROS level significantly to <25% at 50 μM and are more active antioxidants than Xyl-B, which reduced the ROS level to <38% *vs.* PMA. Because none of the ether derivatives, except **9**, **11** and **16**, showed improved antioxidant activity relative to **1**, the hydroxyl group in the 11 position of **1** must be important, but not necessary, for this activity. Additionally, compounds **2** and **5** showed good activity despite the replacement of the hydroxyl group in the 11 position with a methyl or methyl carboxyl group. Amide derivatives containing the hydrophilic groups of **1**, such as **26**, **29**, **30**, **35**, **37** and **39**, exhibited higher antioxidative activities than those with hydrophobic and aromatic groups, such as **27**, **28**, **33**, **34**, **38**. However, **36** is a special case and showed very high antioxidative activity with both hydrophobic propyl and benzyl groups. Compounds with alkyl group substituents that are too large, such as **38**, showed unfavorable antioxidative activity. Moreover, amides containing amino acids, such as **24**, **31**, and **32**, all exhibited marked activities. Specifically, compound **39**, with the mercapto group, showed the highest activity of all compounds tested.

### 2.3. Xyloketal Derivatives Attenuate the Juglone-Induced Reduction of Worm Viability

**Figure 2:**
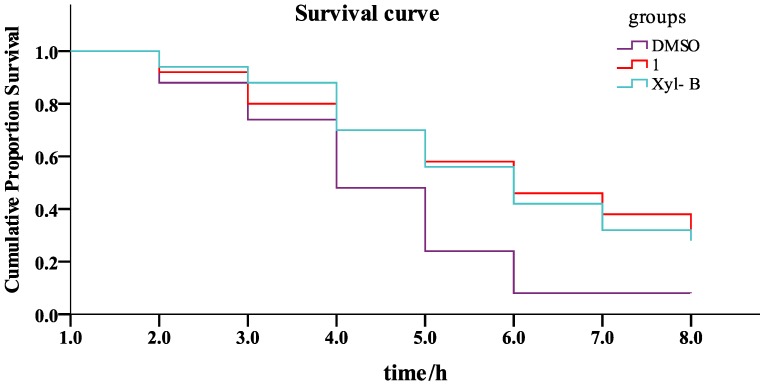
Survival analysis of the representative compounds **1** and Xyl-B in juglone-treated *C. elegans* using the Kaplan–Meier method. The worms were synchronized using sodium hypochlorite and sodium hydroxide. Forty-eight h later, the synchronized worms were further treated with 100 µM of the test compound in their food for an additional 48 h. All worms were then treated with 200 ng/mL of juglone, and the number of dead worms was immediately counted. DMSO was used as a control. The survival curve shows the time-dependent cumulative proportional survival rate. Three independent experiments were performed for each group, and each group contained 50 worms.

**Figure 3:**
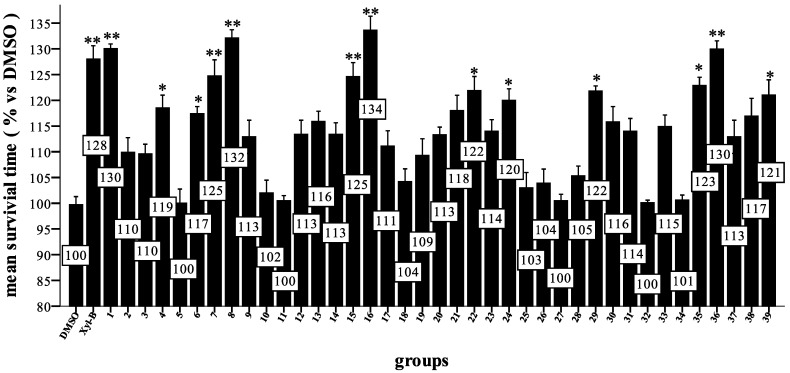
The mean survival times for all of the test compounds analyzed via the Kaplan-Meier method. The worms were synchronized using sodium hypochlorite and sodium hydroxide. Forty-eight h later, the synchronized worms were further treated with 100 µM of the test compound in their food for an additional 48 h. All worms were then treated with 200 ng/mL of juglone, and the number of dead worms was immediately counted. The mean survival time of the DMSO control was normalized to 100%, and the other times are reported relative to this control. (* *p* < 0.05 and ** *p* < 0.005 *vs.* DMSO group, *n* = 50 for each group). Three independent experiments were performed.

As previously reported, juglone oxidants significantly reduce worm viability. A longevity-extending assay using a juglone oxidant to generate ROS and damage the worms was performed with all of the derivatives. The survival time of worms could be extended by pretreatment with the xyloketal compounds. The survival curve showed that the survival rates directly reflected the protective effects of the different compounds (Figure 2). The calculated mean survival times for all test compounds are given in Figure 3.

There were 14 molecules with strong longevity-promoting activity (*p* < 0.05, Figure 3). Of these compounds, Xyl-B, **1**, **8**, **15**, **16** and **36** significantly extended the survival rates (*p* < 0.005 *vs.* DMSO). Notably, compounds **8** and **16** exhibited the strongest protective effects against juglone toxicity and extended the mean survival time of the worms by 132% and 134%, respectively. Similar to the respiratory burst assay, most of the amides containing hydrophilic groups exhibited higher antioxidative activity than those with hydrophobic or aromatic groups; however, **36** was a special case that showed very high antioxidative activity with both hydrophobic propyl and benzyl groups.

The two *in vivo* antioxidative models used above indicated that xyloketal derivatives with benzopyrano pyran skeletons instead of benzopyrano furan skeletons showed increased antioxidative and neuroprotective activities *in vivo*.

### 2.4. Neuroprotective Action of Selected Compounds in the MPP+-Induced C. elegans PD Model

**Figure 4:**
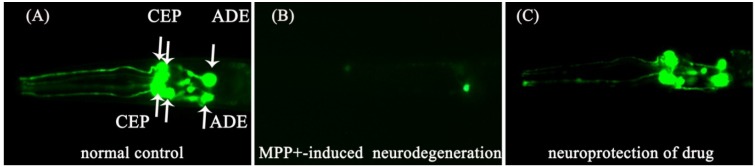
The effects of the tested compounds on 1-methyl-4-phenylpyridinium (MPP+)-induced dopaminergic (DA) neurodegeneration using the MPP+-induced *C. elegans* Parkinson’s disease (PD) model. The representative fluorescence images show dopaminergic neurons in the *BZ555* worm strain. All images were captured using a fluorescent microscope with a 2 ms exposure time. (**A**) Normal control worms exhibited a green fluorescent protein (GFP) signal in cells for three pairs of anterior dopaminergic neurons. The arrows indicate the three DA neuron pairs, which include two CEP neuron pairs and one ADE neuron pair. (**B**) In contrast, worms treated with 1 mM MPP+ for 48 h showed DA neurodegeneration, observed by the loss of GFP signal in the cells, dendrites (CEPs) and axons (ADEs) of DA neurons. (**C**) Treatment with compound **1** significantly attenuated MPP+-induced degeneration in the cell bodies and axons (ADEs), but not in the dendrites (CEPs).

**Figure 5:**
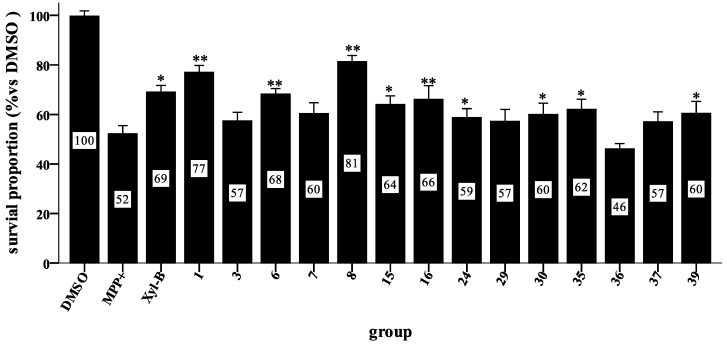
The survival rates for different groups in the MPP+-induced *C. elegans* PD model. Synchronized worms were allowed to hatch to the L1 stage. The worms were treated with 100 µM of the test compound or DMSO, followed by treatment with 1 mM MPP+ before incubating at 20 °C. Approximately 48 h after MPP+ exposure, worm viability was evaluated using a microscope. The survival rate of the DMSO group was normalized to 100% as the blank control, and the MPP+ group treated with 1 mM MPP+ was used as the vehicle control and was approximately 50%. The rates of the other groups are reported relative to the black control. Comparison of the means was performed using a one-way ANOVA followed by a post hoc test (LSD method) (* *p* < 0.05 and ** *p* < 0.005 *vs.* MPP+, *n* = 6 wells for each group). Three independent experiments were performed for each group.

Because ROS play an important role in neurodegenerative diseases, including PD, the 15 compounds with the highest antioxidative activities were selected for further evaluation of their neuroprotective activities using an MPP+-induced *C. elegans* PD model. In *C. elegans*, MPP+ induces dramatic DA degeneration that leads to death (Figure 4). The survival rates indicate the protective results of the tested compounds (Figure 5).

The majority of test compounds provided good protection against MPP+-induced toxicity, which indicates that their neuroprotective activities were likely related to their antioxidative activities. Compounds **1**, **6**, **8** and **16** showed the highest activities of the test compounds (*p* < 0.005 *vs.* MPP+). Compound **8**, the diethylamine salt of **6**, exhibited the strongest antioxidative activity of the examined compounds and increased the MPP+-treated worm survival rate by a remarkable 29% from 52% to 81%.

### 2.5. Neuroprotective Activity of **1** and **8** in the MPP+-Induced Mouse PD Model

**Figure 6:**
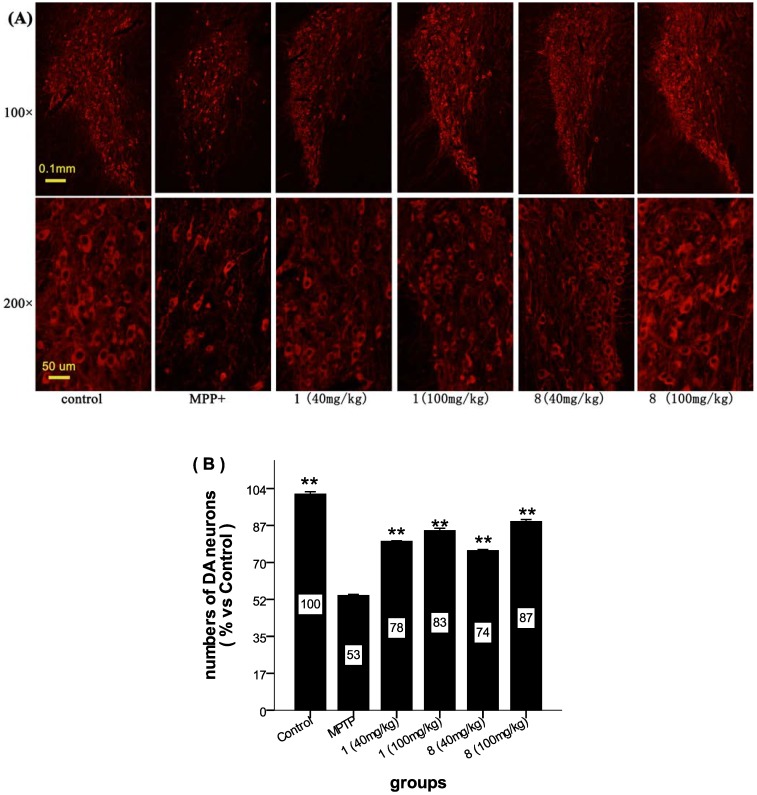
The effects of compounds **1** and **8** on the dopaminergic substantia nigra (SN) neurons using the MPP+-induced mouse PD model. Mice groups were injected with the test compounds (40 mg/kg and 100 mg/kg) or saline for five days following treatment with MPTP (30 mg/kg) or injected with saline for six days. The brain tissues were collected immediately after being transcardially perfused. Immunofluorescent technology was used to observe the DA neurons. (**A**) The upper images were captured with a fluorescent microscope using 100× magnification, and the other images used 200× magnification. (**B**) The number of DA neurons for the different groups. The control group was normalized to 100%, and the other groups are presented as a percentage of the control. Comparison of the means was performed via a one-way ANOVA followed by a post hoc test (LSD method). (** *p* < 0.001 *vs.* MPP+, *n* = 6).

We further investigated the neuroprotective effects of compounds **1** and **8**, the two most promising compounds, using the MPP+-induced *C57BL/6* mouse PD model. The model group was injected with 30 mg/kg of MPTP daily, and 40 mg/kg or 100 mg/kg of **1** and **8** were given to the administration groups intraperitoneally following the MPTP injection. An examination of the dopaminergic neurons in the brain tissue slices from the substantia nigra (SN) was measured using an immunofluorescence technique, and the results are shown in Figure 6.

The staining indicated that the morphology and number of dopaminergic neurons were altered in the MPTP-treated mice, and both **1** and **8** showed remarkable neuroprotective activity (Figure 6A). The number of dopaminergic neurons in the SN tissues was measured using the ImageJ software, and the results are shown in Figure 6B. The control group showed a normal DA neuron number, and the number of DA neurons in this group was normalized to 100%. The MPP+-treated group showed obvious neurodegeneration, with a 53% reduction in the number of dopaminergic neurons in the SN tissue after MPTP injection. Compounds **1** and **8** showed significant improvement in the number of DA neurons, 78% and 74%, respectively, with a 40 mg/kg dose (*p* < 0.001 *vs.* MPP+ group) and 83% and 87%, respectively, with a 100 mg/kg dose (*p* < 0.001 *vs.* MPP+ group). Immunofluorescence analysis of the dopaminergic neurons in the brain tissue slices after treating the *C57BL/6* mice with different drug concentrations showed that **1** and **8** attenuated MPTP toxicity in a dose-dependent manner.

A biological examination of the results revealed that all of the test compounds, except **19** and **28**, showed potent antioxidative activity in both antioxidative models, and compounds containing a benzopyrano pyran skeleton or an amide with hydrophilic substituents demonstrated improved antioxidative activity. For the *C. elegans* PD model, all selected compounds, except **36**, displayed strong neuroprotective activity. Compounds Xyl-B, **1**, **6**, **8** and **16** showed potent activity in both the antioxidative and neuroprotective tests. The mouse PD model further demonstrated the neuroprotective activity of this series of compounds. These findings demonstrate that the newly synthesized xyloketal derivatives are promising candidates for the treatment of PD.

## 3. Experimental Section

### 3.1. Chemistry

All commercially available reagents and solvents were used directly without further purification, unless otherwise stated. ^1^H and ^13^C NMR data were recorded on a 300 MB NMR spectrometer operating at 300 MHz and 75 MHz for ^1^H and ^13^C, respectively, and a 400 MB NMR operating at 400 MHz and 100 MHz for ^1^H and ^13^C, respectively. The deuterated solvents contained tetramethylsilane (TMS) as an internal standard, and all chemical shifts are in ppm (δ). Mass spectra were obtained using a DSQ (low resolution mass spectrometer) and MAT95XP (high resolution mass spectrometer).The purities of all test compounds were at least 95%, as determined by an HPLC equipped with a UV detector at 254 nm with a C18 column (5 μm, 4.6 × 250 mm) at 25 °C.

### 3.2. Synthesis of Intermediates

#### 3.2.1. 5-(Trichloroacetyl)-3,4-dihydro-2*H*-pyran

To a solution of 2,3-dihydropyran (30 g, 0.36 mol) and pyridine (30 g, 0.38 mol) in 200 mL chloroform at −40 °C was added trichloroacetyl chloride (77 g, 0.43 mol) dropwise. The resultant solution was then stirred for 2 h at −40 °C and 1 hour at room temperature before pouring into a mixture of ice and water. The obtained mixture was washed with 1 M HCl (2 × 100 mL), water (100 mL), saturated NaHCO_3_ (100 mL) and saturated brine (100 mL). The separated chloroform layer was dried over anhydrous magnesium sulfate and concentrated *in vacuo* to afford the crude product (71 g, 0.31 mol, 86% yield) as a pale yellow oil. Because it is unstable at room temperature, the crude oil was immediately used in subsequent experiments without further purification. ^1^H NMR (300 MH z, CDCl_3_) δ 8.17 (s, 1H), 4.14 (t, *J* = 5.3 Hz, 2H), 2.40 (t, *J* = 6.4 Hz, 2H), 2.00–1.89 (m, 2H). ^13^C NMR (75 MHz, CDCl_3_) δ 180.26, 161.59, 106.56, 95.78, 67.26, 21.01, 20.02.

#### 3.2.2. 3,4-Dihydro-2*H*-pyran-5-carboxylic Acid

To a solution of crude 5-(trichloroacetyl)-3,4-dihydro-*2H*-pyran (71 g, 0.31 mol) in 150 mL of THF was added 150 mL of an aqueous LiOH·H_2_O (17 g, 0.4 mol) solution. The resultant mixture was stirred for 2 h in a water bath and diluted with 100 mL water. The THF was then removed under reduced pressure on a rotary evaporator, and the remaining solution was washed with chloroform (3 × 100 mL) and adjusted to a pH value of 6 using concentrated HCl. The resultant mixture was extracted with chloroform (3 × 100 mL). The combined organic layer was washed with saturated brine (100 mL), dried over anhydrous magnesium sulfate and concentrated *in vacuo* to afford the crude product (37 g, 0.29 mol, 93% yield) as a light yellow solid. Purification by flash chromatography yielded a white solid. ^1^H NMR (300 MHz, CDCl_3_) δ 7.69 (s, 1H), 4.07 (t, *J* = 5.1 Hz, 2H), 2.27 (t, *J* = 6.3 Hz, 2H), 2.03–1.74 (m, 2H). ^13^C NMR (75 MHz, CDCl_3_) δ 173.55, 157.27, 105.14, 66.86, 21.06, 18.90.

#### 3.2.3. Methyl 3,4-Dihydro-2*H*-pyran-5-carboxylate

To a solution of 3,4-dihydro-*2H*-pyran-5-carboxylic acid (37 g, 0.29 mol) and dimethyl sulfate (37 g, 0.29 mol) in acetone (300 mL) was added potassium carbonate (52 g, 0.38 mol) at room temperature. The resultant solution was stirred overnight, and the acetone was removed under reduced pressure on a rotary evaporator. The obtained mixture was dissolved in 150 mL of water and extracted with ethyl acetate (3 × 100 mL). The combined organic layer was washed with water (100 mL), saturated NaHCO_3_ (100 mL) and saturated brine (100 mL) before drying over anhydrous magnesium sulfate and concentrating *in vacuo.* Flash chromatography purification using ethyl acetate:petroleum ether (1:20) as the eluant afforded the title compound (35 g, 0.25 mol, 84% yield) as a colorless liquid. ^1^H NMR (300 MHz, CDCl_3_) δ 7.46 (t, *J* = 1.1 Hz, 1H), 3.94 (t, *J* = 5.2 Hz, 2H), 3.60 (s, 3H), 2.16 (td, *J* = 6.4, 1.2 Hz, 2H), 1.85 - 1.69 (m, 2H). ^13^C NMR (75 MHz, CDCl_3_) δ 167.79, 155.00, 105.49, 66.36, 50.83, 20.98, 19.10. EI-MS m/z 142 (M).

### 3.3. General Procedure for Synthesizing Compounds **1**–**8**, Using **1** as an Example

#### 3.3.1. Compound **1**

To a suspension of lithium aluminum hydride (0.76 g, 20 mmol) in ether (30 mL) in an ice water bath was added dropwise a solution of methyl 3,4-dihydro-*2H*-pyran-5-carboxylate (1.88 g, 13.2 mmol, 3.2 molar equivalents with respect to phloroglucinol) in ether (20 mL). The resultant mixture was allowed to warm to room temperature and stirred for one additional hour. A 2 M aqueous sodium hydroxide (1.2 g) solution was then added at 0 °C. The resultant mixture was filtered and washed with ether (3 × 50 mL), and the combined filtrates were concentrated *in vacuo* below 10 °C to afford the reduction product as a colorless liquid. This material was diluted with ether (20 mL) and immediately used in subsequent experiments, because it is unstable.

To a suspension of phloroglucinol (0.52 g, 4.1 mmol) and anhydrous magnesium sulfate (2 g) in 40 mL ether at 0 °C was added the ether solution prepared above. *p*-Toluene sulfonic acid (0.1 g) was added after stirring for 1 minute. The resultant mixture was warmed to room temperature and stirred for 30 min. The reaction mixture was then filtered, and the filter cake was washed with ether (3 × 50 mL). The combined filtrates were washed with water (100 mL) and saturated brine (100 mL) before drying over anhydrous magnesium sulfate and concentrating *in vacuo*. Purification via flash chromatography eluting with ether:dichloromethane (2:25) or ethyl acetate:petroleum ether (1:20–1:5) afforded a colorless crystal (0.95 g, 3.0 mmol, 73% yield) as a diastereoisomeric mixture of (±)-**1a** and (±)-**1b** (~1:1). ^1^H NMR (400 MHz, CDCl_3_) δ 6.03 (s, 1H), 5.42 (s, 1H), 5.30 (t, *J* = 3.1 Hz, 1H), 5.23 (t, *J* = 3.1 Hz, 1H), 4.07–3.93 (m, 2H), 3.79–3.63 (m, 2H), 2.79–2.51 (m, 4H), 2.23–2.07 (m, 2H), 1.73–1.59 (m, 8H). ^13^C NMR (101 MHz, CDCl_3_) δ 152.75, 152.71, 151.62, 151.54, 151.16, 151.12, 100.50, 100.41, 100.05, 100.00, 96.74, 96.68, 96.65, 95.70, 63.13, 62.96, 62.84, 62.57, 31.24, 31.20, 31.11, 31.08, 24.56, 24.43, 24.25, 24.14, 23.55, 23.41, 23.31, 22.93, 22.69, 22.54. EI-MS m/z 318 (M). EI-HR-MS m/z found: 318.1457; calcd. for C_18_H_22_O_5_: 318.1462.

#### 3.3.2. Compound **2**

The title compound was obtained as a colorless crystal in 55% yield as a diastereoisomeric mixture of (±)-**2a** and (±)-**2b** (~1:1). ^1^H NMR (400 MHz, CDCl_3_) δ 6.34 (s, 1H), 5.26–5.16 (m, 2H), 4.07–3.92 (m, 2H), 3.75–3.60 (m, 2H), 2.81–2.65 (m, 2H), 2.62–2.41 (m, 2H), 2.19–2.10 (m, 2H), 2.04 (s, 3H), 1.75–1.53 (m, 8H). ^13^C NMR (101 MHz, CDCl_3_) δ 151.57, 135.30, 111.44, 111.42, 101.78, 95.95, 62.64, 62.51, 60.17, 31.73, 31.67, 26.67, 26.56, 24.45, 24.35, 23.35, 23.25, 14.37, 14.03. EI-MS *m/z* 316 (M). EI-HR-MS *m/z* found: 316.1667; calcd. for C_19_H_24_O_4_: 316.1669.

#### 3.3.3. Compound **3**

The title compound was obtained as a colorless crystal in 55% yield as a diastereoisomeric mixture of (±)-**3a** and (±)-**3b** (~1:1). ^1^H NMR (400 MHz, CDCl_3_) δ 14.03 (s, 1H), 5.40–5.33 (m, 2H), 4.05–3.87 (m, 2H), 3.82–3.65 (m, 2H), 2.77–2.51 (m, 7H), 2.23–2.11 (m, 2H), 1.79–1.53 (m, 8H). ^13^C NMR (101 MHz, CDCl_3_) δ 203.34, 162.43, 162.39, 157.04, 156.97, 153.71, 153.59, 105.18, 100.37, 100.28, 98.58, 98.48, 97.47, 97.20, 62.71, 62.62, 62.46, 62.34, 33.08, 30.98, 30.91, 30.74, 30.66, 23.95, 23.89, 23.79, 23.69, 23.49, 23.38, 23.28, 22.75, 22.42. EI-MS *m/z* 360 (M). EI-HR-MS *m/z* found: 360.1565; calcd. for C_20_H_24_O_6_: 360.1567.

#### 3.3.4. Compound **4**

The title compound was obtained as a colorless crystal in 60% yield as a diastereoisomeric mixture of (±)-**4a** and (±)**-4b** (~1:1). ^1^H NMR (400 MHz, CDCl_3_) δ 13.02 (s, 1H), 7.52 (d, *J* = 8.9 Hz, 1H), 6.43 (d, *J* = 8.9 Hz, 1H), 5.36 (d, *J* = 2.6 Hz, 1H), 4.03–3.93 (m, 1H), 3.80–3.69 (m, 1H), 2.80–2.65 (m, 2H), 2.54 (s, 3H), 2.26–2.15 (m, 1H), 1.78–1.53 (m, 4H). ^13^C NMR (101 MHz, CDCl_3_) δ 202.73, 162.65, 159.49, 129.83, 113.53, 108.24, 108.12, 97.10, 62.54, 30.82, 26.08, 23.98, 23.61, 22.78. EI-MS *m/z* 248 (M). EI-HR-MS *m/z* found: 248.1041; calcd. for C_14_H_16_O_4_: 248.1043.

#### 3.3.5. Compound **5**

The title compound was obtained as a colorless crystal in 71% yield as a diastereoisomeric mixture of (±)-**5a** and (±)-**5b** (~1:1). ^1^H NMR (400 MHz, CDCl_3_) δ 6.51 (s, 1H), 5.28 (d, *J* = 2.6 Hz, 2H), 5.26 (d, *J* = 2.6 Hz, 1H), 4.05–3.92 (m, 2H), 3.88 (s, 3H), 3.75–3.57 (m, 2H), 2.93–2.39 (m, 4H), 2.19–2.06 (m, 2H), 1.71–1.57 (m, 8H). ^13^C NMR (101 MHz, CDCl_3_) δ 168.96, 152.36, 152.17, 133.34, 130.83, 128.83, 110.87, 110.79, 105.76, 96.43, 96.33, 65.50, 62.76, 62.34, 60.30, 51.83, 31.54, 31.45, 26.83, 26.27, 24.19, 23.96, 23.55, 23.27. EI-MS *m/z* 360 (M). EI-HR-MS *m/z* found: 360.1566; calcd. for C_20_H_24_O_6_: 360.1567.

#### 3.3.6. Compound **6**

The title compound was obtained as a colorless crystal in 80% yield as a diastereoisomeric mixture of (±)-**6a** and (±)-**6b** (~1:1). ^1^H NMR (400 MHz, CDCl_3_) δ 12.48 (s, 1H), 1H NMR (400 MHz, CDCl3) δ 12.48 (s, 2H), 5.53 (d, *J* = 2.5 Hz, 1H), 5.52 (d, *J* = 2.5 Hz, 1H), 5.37 (d, *J* = 2.6 Hz, 1H), 5.35 (d, *J* = 2.6 Hz, 1H), 4.05–3.64 (m, 8H), 2.88–2.51 (m, 8H), 2.37–2.10 (m, 4H), 1.79–1.51 (m, 16H). ^13^C NMR (101 MHz, CDCl_3_) δ 171.17, 161.28, 161.24, 156.46, 156.38, 151.02, 150.91, 101.93, 101.82, 98.94, 98.85, 98.83, 97.55, 97.49, 94.06, 94.03, 62.90, 62.54, 62.48, 62.42, 30.89, 30.81, 30.75, 30.66, 23.98, 23.81, 23.78, 23.74, 23.55, 23.41, 23.40, 23.32, 22.77, 22.39.

Compound **6** was purified via flash chromatography (petro ether:ethyl acetate = 50:1–30:1) followed by crystallization to afford the pure isomers (±)-**6a** and (±)-**6b**: 

(±)-**6a**: m.p. 171.2–172.1 °C. ^1^H NMR (400 MHz, CDCl_3_) δ 12.47 (s, 1H), 11.31 (s, 1H), 5.51 (d, *J* = 2.4 Hz, 1H), 5.35 (d, *J* = 2.4 Hz, 1H), 4.05–3.67 (m, 4H), 2.87–2.53 (m, 4H), 2.39–2.10 (m, 2H), 1.87–1.48 (m, 8H). ^13^C NMR (101 MHz, CDCl_3_) δ 171.14, 161.24, 156.37, 150.91, 101.93, 98.94, 98.84, 97.55, 94.06, 62.89, 62.54, 30.89, 30.67, 23.99, 23.73, 23.56, 23.39, 23.32, 22.39.

(±)-**6b**: m.p. 178.4–179.5 °C. ^1^H NMR (400 MHz, CDCl_3_) δ 12.47 (s, 1H), 11.31 (s, 1H), 5.53 (d, *J* = 2.4 Hz, 1H), 5.37 (d, *J* = 2.8 Hz, 1H), 3.99–3.68 (m, 4H), 2.82–2.56 (m, 4H), 2.32–2.13 (m, 2H), 1.81–1.52 (m, 8H). ^13^C NMR (101 MHz, CDCl_3_) δ 171.15, 161.28, 156.45, 151.01, 101.82, 98.86, 97.50, 94.04, 62.48, 62.42, 30.81, 30.76, 23.79, 23.54, 23.40, 22.76.

Crystallographic data for (±)-**6a**: Single-crystal growth was performed in EtOAc at room temperature. C_19_H_22_O_7_, crystal dimension 0.42 mm × 0.40 mm × 0.37 mm, space group monoclinic, *C2/c*; unit cell dimensions, *a* = 25.4092 (3) Å, *b* = 12.9938 (2) Å, *c* = 10.67030 (10) Å, volume = 3279.17 (7) Å^3^, *Z* = 8, *D*_calcd_ = 1.468 Mg/m^3^, *m* = 0.939 mm^−1^, *F*000 = 1,536. All single-crystal data were collected via the hemisphere technique on a Bruker SMART 1000 CCD system diffractometer with graphite-monochromated Mo Ká radiation *ë* *=* 1.54178 at 150(2) K. The structure was solved using the direct method. The final *R* value was 0.0322, wR2 = 0.0791 [*I* > 2*ó* (*I*)]. More details are provided in the Supplementary Information.

#### 3.3.7. Compound **7**

The title compound was obtained as a colorless crystal in 90% yield as a diastereoisomeric mixture of (±)-**7a** and (±)-**7b** (~1:1). ^1^H NMR (400 MHz, CDCl_3_) δ 11.97 (s, 1H), 11.96 (s, 1H), 5.34 (apparent t, 2H), 5.32 (d, *J* = 2.6 Hz, 1H), 5.30 (d, *J* = 2.6 Hz, 1H), 4.17–3.93 (m, 4H), 3.91 (s, 6H), 3.83–3.62 (m, 4H), 2.87–2.43 (m, 8H), 2.25–2.01 (m, 4H), 1.77–1.58 (m, 16H). ^13^C NMR (101 MHz, CDCl_3_) δ 172.01, 160.67, 160.65, 156.01, 155.95, 152.77, 152.64, 100.16, 100.08, 99.43, 99.32, 97.38, 97.32, 97.00, 96.97, 95.79, 95.77, 62.93, 62.69, 62.37, 52.03, 31.05, 30.99, 30.47, 30.44, 24.20, 24.02, 23.90, 23.69, 23.51, 23.40, 23.25, 23.01, 22.76, 22.71, 22.55. EI-MS *m/z* 376 (M). EI-HR-MS *m/z* found: 376.1513; calcd. for C_20_H_24_O_7_: 376.1517.

#### 3.3.8. Compound **8**

A solution of **6** (0.2 g, 0.6 mmol) and diethylamine (0.2 g, 6 mmol) in acetone (20 mL) was stirred at room temperature for 30 min. The acetone and excess diethylamine were removed under reduced pressure on a rotary evaporator to afford the title compound as a white foam as a diastereoisomeric mixture of (±)-**8a** and (±)-**8b** (~1:1). ^1^H NMR (400 MHz, CDCl_3_) δ 5.31 (d, *J* = 2.5 Hz, 1H), 5.25 (t, *J* = 2.8 Hz, 1H), 4.13–3.90 (m, 2H), 3.76–3.54 (m, 2H), 3.00 (q, *J* = 7.3 Hz, 4H), 2.74–2.54 (m, 4H), 2.16 (s, 1H), 2.15–2.08 (m, 2H), 1.79–1.54 (m, 8H), 1.29 (t, *J* = 7.3 Hz, 6H).

### 3.4.Typical Method of Etherification of **1** to Prepare **9**–**20**, Using **9** as an Example

#### 3.4.1. Compound **9**

A solution of **1** (0.1 g, 0.31 mmol), potassium carbonate (87 mg, 0.62 mmol) and dimethyl sulfate (78 mg, 0.62 mmol) was stirred in acetone (15 mL) at 60 °C and monitored via thin layer chromatography. The resultant mixture was allowed to cool to room temperature before pouring into water (50 mL). The aqueous layer was extracted with ethyl acetate (3 × 40 mL). The combined organic layers were washed with saturated brine (50 mL), dried over anhydrous magnesium sulfate and concentrated *in vacuo*. Purification via flash chromatography using ethyl acetate:petroleum ether (1:20) as the eluant afforded colorless crystals (87 mg, 0.26 mmol, 85% yield) that were a mixture of diastereoisomers (±)-**9a** and (±)-**9b** (~1:1). ^1^H NMR (400 MHz, CDCl_3_) δ 6.07 (s, 1H), 5.28 (t, *J* = 2.9 Hz, 1H), 5.24 (t, *J* = 3.3 Hz, 1H), 4.09–3.91 (m, 2H), 3.74 (s, 3H), 3.74–3.66 (m, 2H), 2.80–2.47 (m, 4H), 2.23–2.07 (m, 2H), 1.74–1.58 (m, 8H).^13^C NMR (101 MHz, CDCl_3_) δ 156.56, 156.52, 151.66, 151.58, 150.82, 101.10, 101.04, 100.37, 100.28, 96.74, 96.72, 96.66, 91.81, 63.09, 62.92, 62.83, 62.52, 55.33, 31.22, 31.18, 31.07, 31.03, 24.57, 24.44, 24.25, 24.14, 23.57, 23.41, 23.33, 23.04, 22.74, 22.69, 22.54. EI-MS *m/z* 332 (M). EI-HR-MS *m/z* found: 332.1621; calcd. for C_19_H_24_O_5_: 332.1618.

#### 3.4.2. Compound **10**

The title compound was obtained as a colorless crystal in 82% yield as a diastereoisomeric mixture of (±)-**10a** and (±)-**10b** (~1:1). ^1^H NMR (400 MHz, CDCl_3_) δ 5.94 (s, 1H), 5.29 (t, *J* = 3.0 Hz, 1H), 5.23 (dd, *J* = 4.7, 2.5 Hz, 1H), 4.54 (s, 2H), 4.25 (q, *J* = 7.1 Hz, 2H), 4.08–3.90 (m, 2H), 3.80–3.60 (m, 2H), 2.83–2.50 (m, 4H), 2.18 (d, *J* = 27.6 Hz, 2H), 1.78–1.59 (m, 8H), 1.30 (t, *J* = 7.1 Hz, 3H). ^13^C NMR (101 MHz, CDCl_3_) δ 168.83, 154.88, 154.83, 151.60, 151.51, 151.12, 151.09, 101.87, 101.81, 101.50, 101.41, 96.83, 96.76, 96.72, 96.66, 92.81, 65.49, 63.06, 62.87, 62.81, 62.52, 61.16, 31.19, 31.15, 31.06, 31.03, 24.56, 24.41, 24.22, 24.10, 23.56, 23.41, 23.32, 23.01, 22.74, 22.58, 14.10. EI-MS *m/z* 404 (M). EI-HR-MS *m/z* found: 404.1833; calcd. for C_22_H_28_O_7_: 404.1830.

#### 3.4.3. Compound **11**

The title compound was obtained as a colorless crystal in 82% yield as a diastereoisomeric mixture of (±)-**11a** and (±)-**11b** (~1:1). ^1^H NMR (400 MHz, CDCl_3_) δ 6.11 (s, *J* = 7.6 Hz, 1H), 5.57–5.41 (m, 1H), 5.30 (t, *J* = 2.8 Hz, 1H), 5.28–5.24 (m, 1H), 4.46 (d, *J* = 6.5 Hz, 2H), 4.12–3.93 (m, 2H), 3.81–3.61 (m, 2H), 2.85–2.52 (m, 4H), 2.24–2.08 (m, 2H), 1.73–1.61 (m, 8H), 1.58 (s, 6H). ^13^C NMR (101 MHz, CDCl_3_) δ 155.88, 155.83, 151.57, 151.50, 150.84, 150.81, 137.13, 120.10, 101.52, 101.46, 100.32, 100.23, 96.79, 96.73, 96.67, 92.98, 65.04, 63.07, 62.92, 62.82, 62.53, 31.29, 31.25, 31.13, 25.70, 24.62, 24.50, 24.30, 24.19, 23.59, 23.43, 23.35, 23.13, 22.84, 22.70, 22.56, 18.16. EI-MS *m/z* 386 (M). EI-HR-MS *m/z* found: 386.2086; calcd. for C_23_H_30_O_5_: 386.2088.

#### 3.4.4. Compound **12**

The title compound was obtained by reaction in a sealed tube as a colorless crystal in 74% yield as a diastereoisomeric mixture of (±)-**12a** and (±)-**12b** (~1:1). ^1^H NMR (400 MHz, CDCl_3_) δ 6.06 (s, *J* = 7.1 Hz, 1H), 6.09–5.97 (m, 1H), 5.40 (ddd, *J* = 17.3, 3.2, 1.6 Hz, 1H), 5.29 (t, *J* = 2.8 Hz, 1H), 5.28–5.22 (m, 1H), 5.24 (t, *J* = 3.0 Hz, 1H), 4.46 (dt, *J* = 5.0, 1.4 Hz, 2H), 4.10–3.91 (m, 2H), 3.81–3.63 (m, 2H), 2.86–2.46 (m, 4H), 2.22–2.08 (m, 2H), 1.80–1.59 (m, 8H).^13^C NMR (101 MHz, CDCl_3_) δ 155.51, 155.47, 151.56, 151.48, 150.89, 150.85, 133.41, 117.03, 101.48, 101.42, 100.56, 100.47, 96.78, 96.71, 96.68, 92.94, 68.67, 63.16, 63.00, 62.85, 62.55, 31.21, 31.17, 31.11, 31.07, 24.60, 24.48, 24.28, 24.16, 23.59, 23.42, 23.39, 23.31, 23.13, 22.84, 22.66, 22.51. EI-MS *m/z* 358 (M). EI-HR-MS *m/z* found: 358.1767; calcd. for C_21_H_26_O_5_: 358.1775.

#### 3.4.5. Compound **13**

The title compound was obtained by reaction in a sealed tube as a colorless crystal in 97% yield as a diastereoisomeric mixture of (±)-**13a** and (±)-**13b** (~1:1). ^1^H NMR (400 MHz, CDCl_3_) δ 6.05 (s, 1H), 5.28 (t, *J* = 2.8 Hz, 1H), 5.24 (t, *J* = 3.1 Hz, 1H), 4.06–3.95 (m, 2H), 3.85 (t, *J* = 6.4 Hz, 2H), 3.77–3.62 (m, 2H), 2.83–2.47 (m, 4H), 2.23–2.07 (m, 2H), 1.84–1.72 (m, 2H), 1.73–1.59 (m, 8H), 1.01 (t, *J* = 7.4 Hz, 3H).^13^C NMR (101 MHz, CDCl_3_) δ 155.93, 155.89, 151.52, 151.45, 150.73, 150.69, 101.29, 101.23, 100.05, 99.96, 96.70, 96.62, 96.56, 92.53, 69.29, 62.96, 62.81, 62.44, 31.19, 31.14, 31.07, 31.03, 24.49, 24.37, 24.23, 24.11, 23.55, 23.38, 23.31, 23.03, 22.72, 22.65, 22.51, 22.45, 10.53. EI-MS *m/z* 360 (M). EI-HR-MS *m/z* found: 360.1935; calcd. for C_21_H_28_O_5_: 360.1931.

#### 3.4.6. Compound **14**

The title compound was obtained as a colorless crystal in 61% yield as a diastereoisomeric mixture of (±)-**14a** and (±)-**14b** (~1:1). ^1^H NMR (400 MHz, CDCl_3_) δ 6.07 (s, 1H), 5.28 (t, *J* = 2.8 Hz, 1H), 5.24 (t, *J* = 2.8 Hz, 1H), 4.11–3.96 (m, 2H), 3.91 (t, *J* = 6.6 Hz, 2H), 3.76–3.64 (m, 2H), 2.78–2.49 (m, 4H), 2.24–2.05 (m, 2H), 1.87–1.73 (m, 1H), 1.74–1.59 (m, 10H), 0.95 (dd, *J* = 6.6, 0.8 Hz, 6H). ^13^C NMR (101 MHz, CDCl_3_) δ 170.61, 161.36, 161.32, 154.30, 154.22, 150.78, 150.68, 141.49, 126.87, 125.28, 123.72, 101.33, 101.24, 98.04, 97.94, 97.50, 97.25, 97.18, 96.48, 62.84, 62.42, 62.34, 62.21, 40.71, 31.01, 30.92, 30.71, 30.63, 29.78, 29.64, 24.10, 23.90, 23.78, 23.62, 23.49, 23.38, 23.33, 22.88, 22.50. EI-MS *m/z* 388 (M). EI-HR-MS *m/z* found: 388.2245; calcd. for C_23_H_32_O_5_: 388.2244.

#### 3.4.7. Compound **15**

The title compound was obtained as a colorless crystal in 75% yield as a diastereoisomeric mixture of (±)-**15a** and (±)-**15b** (~1:1). ^1^H NMR (400 MHz, CDCl_3_) δ 6.04 (s, 1H), 5.28 (t, *J* = 2.7 Hz, 1H), 5.24 (t, *J* = 3.1 Hz, 1H), 4.14 (q, *J* = 7.1 Hz, 2H), 4.08–3.97 (m, 2H), 3.92 (t, *J* = 6.1 Hz, 2H), 3.78–3.65 (m, 2H), 2.82–2.52 (m, 4H), 2.49 (t, *J* = 7.4 Hz, 2H), 2.24–2.05 (m, 4H), 1.65 (m, 8H), 1.26 (t, *J* = 7.1 Hz, 3H).^13^C NMR (101 MHz, CDCl*_3_*) δ 173.13, 155.70, 151.68, 151.61, 150.91, 101.38, 101.33, 100.49, 100.40, 96.80, 96.73, 96.68, 92.65, 66.72, 63.04, 62.89, 62.55, 60.38, 31.28, 31.24, 31.16, 30.98, 24.65, 24.59, 24.47, 24.33, 24.22, 23.62, 23.46, 23.39, 23.10, 22.82, 22.74, 22.61, 14.20. EI-MS *m/z* 432 (M). EI-HR-MS *m/z* found: 432.2151; calcd. for C_24_H_32_O_7_: 432.2143.

#### 3.4.8. Compound **16**

The title compound was obtained as a colorless crystal in 71% yield as a diastereoisomeric mixture of (±)-**16a** and (±)-**16b** (~1:1). ^1^H NMR (400 MHz, CDCl_3_) δ 6.08 (s, 1H), 5.27 (t, *J* = 2.8 Hz, 1H), 5.24 (t, *J* = 3.1 Hz, 1H), 4.05 (t, *J* = 5.8 Hz, 2H), 4.00 (m, 2H), 3.84 (t, *J* = 5.9 Hz, 2H), 3.78–3.62 (m, 2H), 2.82–2.45 (m, 4H), 2.25–2.07 (m, 2H), 2.06–2.01 (m, 2H), 1.76–1.54 (m, 8H). ^13^C NMR (101 MHz, CDCl_3_) δ 155.61, 155.57, 151.68, 151.61, 150.90, 150.85, 145.18, 128.49, 128.08, 101.14, 101.07, 100.67, 100.58, 96.74, 96.72, 96.67, 92.59, 66.06, 63.08, 62.93, 62.61, 60.93, 31.89, 31.20, 31.15, 31.05, 31.01, 29.67, 28.38, 28.27, 24.53, 24.42, 24.31, 24.18, 23.54, 23.41, 23.36, 23.15, 22.85, 22.69, 22.57. EI-MS *m/z* 376 (M). EI-HR-MS *m/z* found: 376.1885; calcd. for C_21_H_28_O_6_: 376.1880.

#### 3.4.9. Compound **17**

The title compound was obtained as a colorless crystal in 72% yield as a diastereoisomeric mixture of (±)-**17a** and (±)-**17b** (~1:1). ^1^H NMR (400 MHz, CDCl_3_) δ 6.05 (s, 1H), 5.28 (t, *J* = 2.8 Hz, 1H), 5.24 (t, *J* = 3.1 Hz, 1H), 4.12–3.95 (m, 2H), 3.88 (t, *J* = 6.5 Hz, 2H), 3.78–3.60 (m, 2H), 2.85–2.48 (m, 4H), 2.14 (m, 2H), 1.75–1.60 (m, 8H), 1.43–1.17 (m, 10H), 0.89 (t, *J* = 6.9 Hz, 3H). ^13^C NMR (101 MHz, CDCl_3_) δ 156.08, 156.05, 151.65, 151.57, 150.85, 150.81, 101.40, 101.34, 100.16, 100.06, 96.80, 96.73, 96.67, 92.67, 67.96, 63.03, 62.88, 62.79, 62.49, 31.77, 31.33, 31.28, 31.22, 31.18, 29.18, 28.99, 26.06, 24.61, 24.48, 24.32, 24.21, 23.66, 23.49, 23.40, 23.18, 22.89, 22.76, 22.58, 14.03. EI-MS *m/z* 416 (M). EI-HR-MS *m/z* found: 416.2547; calcd. for C_25_H_36_O_5_: 416.2557.

#### 3.4.10. Compound **18**

The title compound was obtained as a colorless crystal in 70% yield as a diastereoisomeric mixture of (±)-**18a** and (±)-**18b** (~1:1). ^1^H NMR (400 MHz, CDCl_3_) δ 7.30 (d, *J* = 8.0 Hz, 2H), 7.18 (d, *J* = 7.9 Hz, 2H), 6.15 (s, 1H), 5.29 (t, *J* = 3.0 Hz, 1H), 5.27–5.22 (m, 1H), 4.94 (s, 2H), 4.11–3.93 (m, 2H), 3.80–3.61 (m, 2H), 2.82–2.51 (m, 4H), 2.36 (s, 3H), 2.19–2.08 (m, 2H), 1.77–1.58 (m, 8H). ^13^C NMR (101 MHz, CDCl_3_) δ 155.82, 155.79, 151.67, 151.59, 150.93, 150.90, 137.47, 134.26, 129.13, 127.33, 101.64, 101.59, 100.66, 100.57, 96.84, 96.75, 96.70, 93.19, 69.88, 63.08, 62.93, 62.86, 62.57, 31.29, 31.26, 31.14, 24.62, 24.50, 24.32, 24.21, 23.61, 23.45, 23.38, 23.24, 22.96, 22.75, 22.61, 21.15. EI-MS *m/z* 422 (M). EI-HR-MS *m/z* found: 422.2086; calcd. for C_26_H_30_O_5_: 422.2088.

#### 3.4.11. Compound **19**

The title compound was obtained as a colorless crystal in 66% yield as a diastereoisomeric mixture of (±)-**19a** and (±)-**19b** (~1:1). ^1^H NMR (400 MHz, CDCl_3_) δ 7.64 (d, *J* = 8.1 Hz, 2H), 7.52 (d, *J* = 8.0 Hz, 2H), 6.10 (s, 1H), 5.31 (t, *J* = 2.9 Hz, 1H), 5.24 (t, *J* = 3.3 Hz, 1H), 5.05 (s, 2H), 4.09–3.93 (m, 2H), 3.77–3.64 (m, 2H), 2.80–2.58 (m, 4H), 2.23–2.07 (m, 2H), 1.75–1.60 (m, 8H). ^13^C NMR (101 MHz, CDCl_3_) δ 155.32, 151.73, 151.65, 151.10, 141.37, 127.12, 125.47, 125.44, 101.58, 101.54, 101.16, 101.08, 96.85, 96.78, 96.74, 93.12, 69.03, 63.19, 63.04, 62.91, 62.63, 31.18, 31.12, 29.67, 24.64, 24.53, 24.35, 24.25, 23.55, 23.37, 23.29, 23.21, 22.66. EI-MS *m/z* 476 (M). EI-HR-MS *m/z* found: 476.1809; calcd. for C_26_H_27_O_5_F_3_: 476.1805.

#### 3.4.12. Compound **20**

The title compound was obtained as a colorless crystal in 71% yield as a diastereoisomeric mixture of (±)-**20a** and (±)-**20b** (~1:1). ^1^H NMR (400 MHz, CDCl_3_) δ 7.40 (s, 1H), 7.32–7.26 (m, 3H), 6.10 (s, 1H), 5.31 (t, *J* = 2.9 Hz, 1H), 5.28–5.20 (m, 1H), 4.96 (s, 2H), 4.06–3.94 (m, 2H), 3.80–3.63 (m, 2H), 2.84–2.52 (m, 4H), 2.24–2.08 (m, 2H), 1.80–1.59 (m, 8H). ^13^C NMR (101 MHz, CDCl_3_) δ 155.36, 155.33, 151.62, 151.54, 151.01, 150.98, 139.33, 134.40, 129.77, 127.89, 127.09, 125.08, 101.57, 101.51, 100.99, 100.90, 96.81, 96.75, 96.69, 93.06, 69.02, 63.18, 63.02, 62.86, 62.56, 31.17, 31.13, 31.07, 31.04, 24.60, 24.47, 24.25, 24.14, 23.59, 23.43, 23.37, 23.28, 22.96, 22.66, 22.51. EI-MS *m/z* 442 (M). EI-HR-MS *m/z* found: 442.1545; calcd. for C_25_H_27_O_5_Cl_1_: 442.1542. 

### 3.5. Typical Method to Prepare **21**–**23**, Using **21** as an Example

#### 3.5.1. Compound **21**

A solution of compound **7** (0.1 g, 0.27 mmol) and ammonia (5 mL) in acetone (10 mL) was stirred in a sealed tube at 60 °C for 5 h and monitored via thin layer chromatography. The resultant mixture was then poured into water (50 mL) and extracted with ethyl acetate (3 × 30 mL). The combined organic layers were washed with saturated brine (50 mL), dried over anhydrous magnesium sulfate and concentrated *in vacuo*. Purification via flash chromatography using petroleum ethyl acetate: ether (3:2) as the eluant afforded the title compound (80 mg, 0.22 mmol, 82%) as a colorless crystal consisting of a diastereoisomeric mixture of (±)-**21a** and (±)-**21b** (~1:1). ^1^H NMR (400 MHz, CDCl_3_) δ 14.11 (s, 1H), 14.11 (s, 1H), 8.17 (s, 2H), 5.71 (s, 2H), 5.54–5.38 (m, 2H), 5.38–5.17 (m, 2H), 4.09–3.85 (m, 4H), 3.85–3.63 (m, 4H), 2.85–2.51 (m, 8H), 2.29–2.08 (m, 4H), 1.80–1.54 (m, 16H). ^13^C NMR (101 MHz, CDCl_3_) δ 172.74, 161.84, 161.80, 155.03, 154.96, 151.21, 151.11, 101.30, 101.21, 98.21, 98.12, 97.64, 97.36, 97.29, 96.00, 62.82, 62.42, 62.35, 62.24, 31.02, 30.94, 30.82, 30.74, 24.09, 23.97, 23.90, 23.64, 23.51, 23.45, 22.87, 22.51. EI-MS *m/z* 361 (M). EI-HR-MS *m/z* found: 361.1520; calcd. for C_19_H_23_O_6_N_1_: 361.1520.

#### 3.5.2. Compound **22**

The title compound was obtained with compound **7** similar to the method for synthesizing compound **21** by replacing ammonia with methylamine as a colorless crystal in 90% yield as a diastereoisomeric mixture of (±)-**22a** and (±)-**22b** (~1:1). ^1^H NMR (400 MHz, CDCl_3_) δ 14.39 (s, 1H), 14.38 (s, 1H), 8.33 (s, 2H), 5.47–5.38 (m, 2H), 5.38–5.26 (m, 2H), 4.04–3.87 (m, 4H), 3.84–3.64 (m, 4H), 2.97 (s, 3H), 2.95 (s, 3H), 2.85–2.51 (m, 8H), 2.23–2.09 (m, 4H), 1.76–1.59 (m, 16H). ^13^C NMR (101 MHz, CDCl_3_) δ 171.22, 161.26, 161.22, 154.18, 154.12, 150.70, 150.60, 101.40, 101.31, 98.01, 97.92, 97.60, 97.29, 97.22, 96.64, 62.85, 62.59, 62.47, 60.33, 31.07, 30.99, 30.79, 30.71, 26.03, 24.14, 23.97, 23.80, 23.73, 23.63, 23.60, 23.43, 23.30, 23.25, 22.89, 22.54, 14.16. EI-MS *m/z* 375 (M). EI-HR-MS *m/z* found: 375.1666; calcd. for C_20_H_25_O_6_N_1_: 375.1676.

#### 3.5.3. Compound **23**

The title compound was obtained with compound **7** similar to the method for synthesizing compound **21** by replacing ammonia with butylamine as a colorless crystal in 81% yield as a diastereoisomeric mixture of (±)-**23a** and (±)-**23b** (~1:1). ^1^H NMR (400 MHz, CDCl_3_) δ 14.43 (s, 1H), 8.43 (s, 1H), 5.44–5.38 (m, 1H), 5.34–5.27 (m, 1H), 4.04–3.85 (m, 2H), 3.84–3.63 (m, 2H), 3.51–3.27 (m, 2H), 2.90–2.47 (m, 4H), 2.31–2.07 (m, 2H), 1.77–1.51 (m, 10H), 1.49–1.38 (m, 2H), 0.95 (t, *J* = 7.3 Hz, 3H). ^13^C NMR (101 MHz, CDCl_3_) δ 170.43, 161.33, 161.29, 154.16, 154.09, 150.86, 150.77, 101.36, 101.26, 97.87, 97.78, 97.57, 97.27, 97.20, 96.63, 96.61, 62.84, 62.43, 61.99, 61.91, 39.36, 38.88, 31.34, 31.24, 31.08, 30.98, 30.89, 30.80, 24.13, 23.94, 23.78, 23.64, 23.50, 23.42, 23.37, 22.91, 22.52, 20.15, 19.94, 13.67, 13.57. EI-MS *m/z* 417 (M). EI-HR-MS *m/z* found: 417.2141; calcd. for C_23_H_31_O_6_N_1_: 417.2146.

### 3.6. Typical Method to Prepare **24**–**39**, Using **24** as an Example

#### 3.6.1. Compound **24**

To a solution of compound **6** (0.1 g, 0.28 mmol), Ala-OMe.HCl (70 mg, 0.56 mmol) and BOP (0.18 g, 0.42 mmol) in dichloromethane (5 mL) was added DIEA (0.5 g, 3.9 mmol). This solution was stirred at room temperature and monitored by thin layer chromatography. The resultant mixture was then diluted with 100 mL of ethyl acetate and washed with 1 M HCl (50 mL) and saturated brine (50 mL) before drying over anhydrous magnesium sulfate and concentrating *in vacuo*. Purification by flash chromatography using ethyl acetate:petroleum ether (1:10) as the eluant afforded the title compound (0.11 g, 0.25 mmol, 90% yield) as a colorless crystal consisting of a diastereoisomeric mixture of (±)-**24a** and (±)-**24b** (~1:1). ^1^H NMR (400 MHz, CDCl_3_) δ 14.06 (s, 1H), 9.01 (s, 1H), 5.53–5.42 (m, 1H), 5.38–5.28 (m, 1H), 4.89–4.62 (m, 1H), 4.14–4.03 (m, 1H), 4.02–3.90 (m, 1H), 3.88–3.80 (m, 1H), 3.78 (s, 3H), 3.75–3.66 (m, 1H), 2.80–2.58 (m, 4H), 2.25–2.12 (m, 2H), 1.84–1.56 (m, 8H), 1.51 (d, *J* = 7.1 Hz, 3H). ^13^C NMR (101 MHz, CDCl_3_) δ 173.34, 169.88, 161.34, 154.61, 151.22, 101.08, 97.98, 97.70, 97.17, 96.21, 62.39, 61.74, 52.42, 48.14, 30.97, 30.84, 29.68, 24.29, 24.07, 23.83, 23.64, 23.17, 22.90, 18.53. EI-MS *m/z* 447 (M). EI-HR-MS *m/z* found: 447.1886; calcd. for C_23_H_29_O_8_N_1_: 447.1888.

#### 3.6.2. Compound **25**

The title compound was obtained as a colorless crystal in 54% yield as a diastereoisomeric mixture of (±)-**25a** and (±)-**25b** (~1:1). ^1^H NMR (400 MHz, CDCl_3_) δ 12.93 (s, 1H), 5.44 (dd, *J* = 6.0, 2.5 Hz, 1H), 5.34 (dd, *J* = 5.1, 2.7 Hz, 1H), 4.21–3.88 (m, 2H), 3.84–3.68 (m, 2H), 2.94 (t, *J* = 7.3 Hz, 2H), 2.76–2.50 (m, 4H), 2.24–2.10 (m, 2H), 1.72–1.59 (m, 8H), 1.56–1.30 (m, 4H), 0.94 (t, *J* = 7.3 Hz, 3H). EI-MS *m/z* 434 (M). EI-HR-MS *m/z* found: 434.1759; calcd. for C_23_H_30_O_6_S_1_: 434.1758.

#### 3.6.3. Compound **26**

The title compound was obtained as a light yellow crystal in 98% yield as a diastereoisomeric mixture of (±)-**26a** and (±)-**26b** (~1:1). ^1^H NMR (400 MHz, CDCl_3_) δ 14.36 (s, 1H), 8.53 (s, 1H), 7.14 (dd, *J* = 5.1, 1.2 Hz, 1H), 6.92 (dd, *J* = 5.1, 3.4 Hz, 1H), 6.88 (dd, *J* = 3.4, 0.9 Hz, 1H), 5.38–5.27 (m, 2H), 4.03–3.90 (m, 1H), 3.81 (m, 1H), 3.76–3.56 (m, 4H), 3.30–3.01 (m, 2H), 2.83–2.51 (m, 4H), 2.24–2.08 (m, 2H), 1.85–1.57 (m, 8H). ^13^C NMR (101 MHz, CDCl_3_) δ 170.59, 161.31, 154.27, 154.18, 150.74, 150.64, 141.46, 126.87, 125.31, 123.75, 101.30, 101.20, 98.03, 97.93, 97.47, 97.22, 97.14, 96.43, 62.42, 62.34, 62.20, 40.70, 30.95, 30.86, 30.65, 30.57, 29.76, 29.65, 24.06, 23.85, 23.76, 23.59, 23.44, 23.32, 22.87, 22.46. EI-MS *m/z* 471 (M). EI-HR-MS *m/z* found: 471.1712; calcd. for C_25_H_29_O_6_N_1_S_1_: 471.1710.

#### 3.6.4. Compound **27**

The title compound was obtained as a light yellow crystal in 70% yield as a diastereoisomeric mixture of (±)-**27a** and (±)-**27b** (~1:1). ^1^H NMR (400 MHz, CDCl_3_) δ 14.12 (s, 1H), 8.73 (s, 1H), 7.31–7.19 (m, 4H), 5.36 (s, 1H), 5.30 (s, 1H), 4.63 (dd, *J* = 15.2, 5.9 Hz, 1H), 4.48 (dd, *J* = 15.1, 4.8 Hz, 1H), 4.00–3.85 (m, 1H), 3.75–3.56 (m, 3H), 2.79–2.50 (m, 4H), 2.21–2.05 (m, 2H), 1.76–1.53 (m, 8H). ^13^C NMR (101 MHz, CDCl_3_) δ 170.52, 161.40, 154.62, 154.54, 154.49, 150.90, 150.81, 136.89, 133.13, 128.85, 128.78, 101.49, 101.39, 98.15, 98.07, 97.66, 97.34, 97.27, 96.43, 62.85, 62.48, 62.16, 62.07, 42.58, 31.05, 30.97, 30.81, 30.73, 24.13, 23.98, 23.64, 23.59, 23.43, 22.91, 22.57. EI-MS *m/z* 485 (M). EI-HR-MS *m/z* found: 485.1606; calcd. for C_26_H_28_O_6_N_1_Cl_1_: 485.1600.

#### 3.6.5. Compound **28**

The title compound was obtained as a colorless crystal in 100% yield as a diastereoisomeric mixture of (±)-**28a** and (±)-**28b** (~1:1). ^1^H NMR (400 MHz, CDCl_3_) δ 14.59 (s, 1H), 8.41 (s, 1H), 5.44–5.37 (m, 1H), 5.37–5.27 (m, 1H), 4.00–3.88 (m, 2H), 3.83–3.75 (m, 1H), 3.75–3.65 (m, 1H), 2.82–2.53 (m, 4H), 2.23–2.14 (m, 2H), 2.14–2.07 (m, 9H), 1.80–1.69 (m, 8H), 1.68–1.57 (m, 6H). ^13^C NMR (101 MHz, CDCl_3_) δ 169.93, 161.59, 161.54, 153.84, 153.76, 150.68, 150.59, 101.41, 101.29, 97.56, 97.26, 97.15, 62.85, 62.37, 61.69, 52.21, 41.69, 36.47, 31.13, 31.01, 30.97, 30.86, 29.68, 29.51, 24.37, 24.21, 24.13, 23.90, 23.72, 23.45, 23.30, 23.17, 23.01, 22.56, 14.18. EI-MS *m/z* 495 (M). EI-HR-MS *m/z* found: 495.2612; calcd. for C_29_H_37_O_6_N_1_: 495.2615.

#### 3.6.6. Compound **29**

The title compound was obtained as a colorless crystal in 42% yield as a diastereoisomeric mixture of (±)-**29a** and (±)-**29b** (~1:1). ^1^H NMR (300 MHz, CDCl_3_) δ 13.89 (s, 1H), 10.60 (s, 1H), 8.17 (s, 1H), 7.68 (d, *J* = 8.0 Hz, 1H), 7.60 (d, *J* = 7.7 Hz, 1H), 7.44 (t, *J* = 7.8 Hz, 1H), 5.54 (t, *J* = 3.2 Hz, 1H), 5.45–5.33 (m, 1H), 4.05–3.73 (m, 4H), 2.69–2.63 (m, 4H), 2.34–2.14 (m, 2H), 1.75–1.60 (m, 8H). EI-MS *m/z* 480 (M).

#### 3.6.7. Compound **30**

The title compound was obtained as a colorless crystal in 71% yield as a diastereoisomeric mixture of (±)-**30a** and (±)-**30b** (~1:1). ^1^H NMR (400 MHz, CDCl_3_) δ 14.42 (s, 1H), 8.41 (s, 1H), 7.09 (d, *J* = 8.4 Hz, 2H), 6.75 (d, *J* = 8.5 Hz, 1H), 5.35–5.30 (m, 1H), 5.30–5.25 (m, 1H), 4.13–3.85 (m, 2H), 3.78–3.58 (m, 4H), 2.90–2.77 (m, 2H), 2.74–2.58 (m, 4H), 2.19–2.11 (m, 2H), 1.74–1.62 (m, 8H). ^13^C NMR (101 MHz, CDCl_3_) δ 170.54, 161.34, 161.29, 154.33, 154.15, 150.77, 150.66, 131.05, 129.91, 115.37, 101.36, 98.06, 97.50, 97.27, 97.20, 97.07, 97.01, 96.56, 62.92, 62.35, 40.76, 34.55, 31.03, 30.94, 30.71, 30.65, 29.68, 26.90, 24.13, 23.91, 23.64, 23.49, 23.38, 23.27, 22.90, 22.63, 22.48. EI-MS *m/z* 481 (M). EI-HR-MS *m/z* found: 481.2087; calcd. for C_27_H_31_O_7_N_1_: 481.2095.

#### 3.6.8. Compound **31**

The title compound was obtained as a colorless crystal in 51% yield as a diastereoisomeric mixture of (±)-**31a** and (±)-**31b** (~1:1). ^1^H NMR (400 MHz, CDCl_3_) δ 14.02 (s, 1H), 8.87 (s, 1H), 5.46 (s, 1H), 5.34 (s, 1H), 4.93–4.62 (m, 1H), 4.12–3.89 (m, 2H), 3.90–3.39 (m, 5H), 2.87–2.49 (m, 4H), 2.28–2.07 (m, 2H), 1.85–1.45 (m, 12H), 0.97 (d, *J* = 5.6 Hz, 6H). ^13^C NMR (101 MHz, CDCl3) δ 173.31, 170.16, 161.39, 154.64, 154.59, 151.26, 151.17, 101.27, 101.17, 98.05, 97.97, 97.76, 97.30, 97.22, 96.29, 62.79, 62.40, 61.61, 52.17, 50.81, 41.61, 31.03, 30.93, 25.03, 24.40, 24.25, 24.17, 24.05, 23.90, 23.67, 23.46, 23.28, 23.16, 22.93, 22.79, 22.57, 22.08. EI-MS *m/z* 489 (M). EI-HR-MS *m/z* found: 489.2351; calcd. for C_26_H_35_O_8_N_1_: 489.2357.

#### 3.6.9. Compound **32**

The title compound was obtained as a light yellow crystal in 80% yield as a diastereoisomeric mixture of (±)-**32a** and (±)-**32b** (~1:1). ^1^H NMR (400 MHz, CDCl_3_) δ 13.97–13.74 (m, 1H), 9.28–9.06 (m, 1H), 5.51–5.40 (m, 1H), 5.40–5.28 (m, 1H), 5.11–4.91 (m, 1H), 4.13–3.88 (m, 2H), 3.82–3.59 (m, 5H), 3.51–3.04 (m, 2H), 2.84–2.47 (m, 4H), 2.27–2.09 (m, 2H), 1.77–1.54 (m, 8H). EI-MS *m/z* 479 (M). EI-HR-MS *m/z* found: 479.1605; calcd. for C_23_H_29_O_8_N_1_S_1_: 479.1608.

#### 3.6.10. Compound **33**

The title compound was obtained as a light yellow crystal in 69% yield as a diastereoisomeric mixture of (±)-**33a** and (±)-**33b** (~1:1). ^1^H NMR (400 MHz, CDCl_3_) δ 14.00 (s, 1H), 10.45 (s, 1H), 7.67–7.45 (m, 2H), 7.13–6.94 (m, 2H), 5.51 (d, *J* = 2.6 Hz, 1H), 5.34 (d, *J* = 2.6 Hz, 1H), 4.10–3.61 (m, 4H), 2.92–2.49 (m, 4H), 2.33–2.11 (m, 2H), 1.89–1.56 (m, 8H). ^13^C NMR (101 MHz, CDCl_3_) δ 168.80, 161.71, 160.63, 158.21, 154.78, 150.48, 133.82, 122.58, 122.50, 115.71, 115.49, 101.74, 98.33, 97.97, 97.35, 96.55, 62.93, 62.09, 30.82, 24.09, 23.88, 23.38, 23.33, 22.42. EI-MS *m/z* 455 (M). EI-HR-MS *m/z* Found: 455.1741; calcd. for C_25_H_26_O_6_N_1_ F_1_: 455.1739.

#### 3.6.11. Compound **34**

The title compound was obtained as a light yellow crystal in 72% yield as a diastereoisomeric mixture of (±)-**34a** and (±)-**34b** (~1:1). ^1^H NMR (400 MHz, CDCl_3_) δ 13.94 (s, 1H), 10.52 (s, 1H), 7.55 (d, *J* = 8.8 Hz, 2H), 7.30 (d, *J* = 8.8 Hz, 2H), 5.58–5.47 (m, 1H), 5.39–5.30 (m, 1H), 4.06–3.62 (m, 4H), 2.84–2.56 (m, 4H), 2.31–2.12 (m, 2H), 1.90–1.53 (m, 8H). ^13^C NMR (101 MHz, CDCl3) δ 168.88, 161.82, 161.77, 154.99, 154.91, 150.57, 150.48, 136.48, 129.24, 129.00, 128.86, 122.22, 121.96, 101.79, 101.68, 98.39, 98.30, 98.03, 97.38, 97.31, 96.83, 96.75, 96.60, 62.95, 62.50, 62.09, 62.01, 60.36, 30.95, 30.84, 30.73, 24.12, 23.93, 23.59, 23.37, 23.24, 22.85, 22.43, 14.16. EI-MS *m/z* 471 (M). EI-HR-MS *m/z* found: 471.1441; calcd. for C_25_H_26_O_6_N_1_Cl_1_: 471.1443.

#### 3.6.12. Compound **35**

The title compound was obtained as a colorless crystal in 64% yield as a diastereoisomeric mixture of (±)-**35a** and (±)-**35b** (~1:1). ^1^H NMR (400 MHz, CDCl_3_) δ 14.11 (s, 1H), 8.77 (s, 1H), 5.46–5.38 (m, 1H), 5.39–5.30 (m, 1H), 4.02–3.89 (m, 2H), 3.86–3.80 (m, 2H), 3.79–3.50 (m, 4H), 2.78–2.55 (m, 4H), 2.45 (s, 1H), 2.25–2.11 (m, 2H), 1.81–1.60 (m, 8H). ^13^C NMR (101 MHz, CDCl_3_) δ 171.48, 161.28, 154.49, 150.92, 150.82, 101.45, 98.20, 97.69, 97.34, 97.27, 96.47, 62.85, 62.56, 62.46, 62.36, 62.26, 42.20, 31.07, 30.99, 30.84, 30.76, 24.12, 23.95, 23.88, 23.61, 23.53, 23.47, 22.57, 14.18. EI-MS *m/z* 405 (M). EI-HR-MS *m/z* found: 405.1780; calcd. for C_21_H_27_O_7_N_1_: 405.1782.

#### 3.6.13. Compound **36**

The title compound was obtained as a colorless crystal in 77% yield as a diastereoisomeric mixture of (±)-**36a** and (±)-**36b** (~1:1). ^1^H NMR (400 MHz, CDCl_3_) δ 7.25–7.11 (m, 5H), 5.41–5.06 (m, 2H), 4.05–3.85 (m, 2H), 3.72 (s, 2H), 3.71–3.53 (m, 2H), 2.91–2.75 (m, 1H), 2.75–2.42 (m, 4H), 2.25–2.00 (m, 2H), 1.81–1.33 (m, 8H), 1.04 (d, *J* = 6.3 Hz, 6H). EI-MS *m/z* 493 (M). EI-HR-MS *m/z* found: 493.2453; calcd. for C_29_H_35_O_6_N_1_: 493.2459.

#### 3.6.14. Compound **37**

The title compound was obtained as a colorless crystal in 60% yield as a diastereoisomeric mixture of (±)-**37a** and (±)-**37b** (~1:1). ^1^H NMR (400 MHz, CDCl_3_) δ 14.43 (s, 1H), 5.42–5.33 (m, 1H), 5.32–5.25 (m, 1H), 4.04–3.85 (m, 2H), 3.79–3.64 (m, 2H), 3.57–3.27 (m, 2H), 2.77–2.42 (m, 6H), 2.24 (s, 6H), 2.17–2.07 (m, 2H), 1.70–1.52 (m, 8H). ^13^C NMR (101 MHz, CDCl_3_) δ 170.28, 161.19, 161.15, 154.09, 154.02, 153.96, 150.95, 150.87, 150.73, 150.65, 101.21, 101.11, 101.05, 100.95, 97.79, 97.75, 97.71, 97.67, 97.44, 97.13, 97.06, 96.58, 96.45, 77.32, 77.00, 76.68, 62.74, 62.31, 61.93, 61.86, 61.71, 61.64, 57.47, 44.98, 39.10, 36.91, 31.77, 30.94, 30.89, 30.85, 30.80, 30.75, 30.66, 29.48, 29.45, 29.19, 26.94, 24.16, 24.12, 24.02, 23.82, 23.62, 23.53, 23.40, 23.28, 22.78, 22.53, 22.38, 13.96. EI-MS *m/z* 432 (M). EI-HR-MS *m/z* found: 432.2262; calcd. for C_23_H_32_O_6_N_2_: 432.2255.

#### 3.6.15. Compound **38**

The title compound was obtained as a colorless crystal in 71% yield as a diastereoisomeric mixture of (±)-**38a** and (±)-**38b** (~1:1). ^1^H NMR (400 MHz, CDCl_3_) δ 14.46 (s, 1H), 8.44 (s, 1H), 5.41 (d, *J* = 2.6 Hz, 1H), 5.31 (d, *J* = 2.5 Hz, 1H), 4.03–3.85 (m, 2H), 3.84–3.62 (m, 2H), 3.55–3.22 (m, 2H), 2.91–2.47 (m, 4H), 2.25–2.09 (m, 2H), 1.77–1.54 (m, 10H), 1.40–1.21 (m, 18H), 0.87 (t, *J* = 6.8 Hz, 3H). ^13^C NMR (101 MHz, CDCl_3_) δ 170.72, 161.59, 154.45, 154.38, 151.16, 151.07, 102.95, 101.66, 101.56, 100.31, 98.17, 97.87, 97.57, 97.50, 96.93, 67.56, 63.18, 62.84, 62.75, 62.35, 62.28, 39.65, 39.54, 35.12, 32.20, 31.36, 31.27, 31.18, 31.09, 29.91, 29.88, 29.62, 28.81, 27.37, 25.67, 24.44, 24.41, 24.30, 24.24, 24.09, 23.80, 23.72, 23.22, 23.13, 22.96, 22.82, 14.38. EI-MS *m/z* 529 (M). EI-HR-MS *m/z* found: 529.3404; calcd. for C_31_H_47_O_6_N_1_: 529.3398.

#### 3.6.16. Compound **39**

The title compound was obtained as a colorless crystal in 61% yield as a diastereoisomeric mixture of (±)-**39a** and (±)-**39b** (~1:1). ^1^H NMR (400 MHz, CDCl_3_) δ 14.21 (s, 1H), 8.74 (s, 1H), 5.49–5.38 (m, 1H), 5.38–5.27 (m, 1H), 4.06–3.88 (m, 2H), 3.84–3.64 (m, 3H), 3.62–3.48 (m, 1H), 2.86–2.51 (m, 6H), 2.25–2.11 (m, 2H), 2.04 (s, 1H), 1.82–1.57 (m, 8H). ^13^C NMR (101 MHz, CDCl_3_) δ 170.70, 161.42, 161.38, 154.51, 154.45, 150.96, 150.87, 101.44, 101.35, 98.16, 98.07, 97.73, 97.33, 97.27, 96.49, 62.84, 62.45, 62.36, 42.37, 31.07, 30.99, 30.88, 30.80, 24.52, 24.12, 24.02, 23.97, 23.67, 23.60, 23.53, 23.47, 22.92, 22.59. EI-MS *m/z* 421 (M). EI-HR-MS *m/z* found: 421.1551; calcd. for C_21_H_27_O_6_N_1_S_1_: 421.1554.

### 3.7. Biological Evaluation

#### 3.7.1. Respiratory Burst Assay

The test compounds, PMA and H_2_DCFDA, were prepared as stock solutions in DMSO at concentrations of 200 mM, 1 mg/mL and 10 mg/mL, respectively, and stored at −20 °C in the dark. The *AB* strain of zebrafish was used for the respiratory burst assay. These zebrafish were bred and maintained in an Aquatic Habitats system at pH 7.6 with a flow rate of 75 L/d.

The *AB* strain of zebrafish was bred to yield embryos. The embryos were collected, rinsed in egg water, transferred to Petri dishes and held at 28 °C for 72 h post-fertilization (hpf). ROS production was measured in whole zebrafish embryos at 72 hpf. A 96-well microplate was used to measure the respiratory burst, and each well contained one embryo in 100 µL of egg water. To each embryo, 100 µL of a 100 μM solution of test compound in 0.1% DMSO or 0.1% DMSO as a control was added. After incubating the plate for 30 min at 28 °C, 50 µL of 2 µg/mL H2DCFDA in 0.2% DMSO and 800 ng/mL PMA in 0.1% DMSO were added to each well for a final concentrations of 50 μM for each compound, 500 ng/mL for H_2_DCFDA, 0.15% for DMSO and 200 ng/mL for PMA. Light was avoided when PMA and H2DCFDA were used. All solutions were diluted using egg water. The fluorescence was measured using a fluorescence microplate reader at 28 °C with the excitation and emission filters set to 485 and 530 nm, respectively. The microplates were incubated for 150 min at 28 °C without light after adding all solutions, and the fluorescence of each well was measured. The data from six individual embryos per experiment were averaged, and three independent experiments were performed. The fluorescence of the control was treated as 100%, and the measurements of the test compounds are reported relative to the control.

#### 3.7.2. Worm Strains and Maintenance

All worm strains were obtained from the Caenorhabditis Genetics Center (CGC, 321 Church Street S.E. Minneapolis, MN, USA). Nematodes were cultured in a standard nematode growth medium (NGM) agar in 60-mm Petri plates and maintained at 20 °C in a temperature-controlled incubator. Living *Escherichia coli* bacteria (*OP50*) provided the food source, and 200 μL of bacteria were added to the surface of the NGM plates. The wild-type strain (*N2*) was used for the longevity-extending assay. The transgenic strain, *BZ555* [*Pdat-1::GFP*], containing the *Pdat-1::GFP*-linked reporter was used to establish the neuroprotective model and to visualize DA neuron expression.

To obtain age-synchronized worms, plates containing worm-laid eggs were washed with M9 buffer and treated using a standard synchronizing protocol of 2% sodium hypochlorite and 5 M sodium hydroxide to dissolve the worms. The eggs were then collected via centrifugation, rinsed thrice with M9 buffer, transferred to the surface of the NGM plates and allowed to hatch in the incubator at 20 °C.

#### 3.7.3. Longevity-Extending Assay

The worms that hatched 48 h after synchronization were transferred to standard NGM plates for pretreatment, and the test compounds were diluted in *OP50* and added to the surface at a final concentration of 300 µM before incubating overnight at 35 °C. For the control, plates were pretreated with the same amount of DMSO as that used for the drug groups. A total of 30 worms were added to each plate, with 2 plates for each group. After 24 h, the worms were transferred to new plates pretreated with the test compounds and incubated for 24 h. A total of 50 worms from each group were hatched and transferred to standard NGM plates containing juglone in the agar at a final concentration of 500 µM. Then, the number of dead worms was recorded every hour; each experiment was repeated three times.

#### 3.7.4. Activities of Anti-Parkinson’s Disease with the *C. elegans* Model

The synchronized eggs were allowed to hatch overnight at 20 °C and reach the L1 stage. Neuroprotective activity was investigated using 96-well plates. To each well, 30 µL of water containing 10% *OP50* (v/v) and either a test compound, DMSO blank or vehicle control were added. Next, 10 µL of water containing 20 L1 stage worms were added to each well followed by 10 µL of 5 mM MPP+ for a total volume of 50 µL. The final concentration for the test compounds was 100 µM in 0.1% DMSO, with 1 mM of MPP+. The vehicle control contained 10 µL of water instead of the MPP+ solution. Approximately 48 h after exposure to MPP+, worm viability and DA neuron degeneration were observed using a microscope. The survival rate of the DMSO group was considered to be 100%, and the other groups were normalized as the percent of the vehicle control. The desired MPP+ concentration was set by trial and error, such that the survival rate of worms incubated with only MPP+ was approximately 50% of vehicle control. To visualize the DA neurons, the worms were placed on 2% agarose pads and immobilized with 3 mM levamisole before examining under a fluorescent microscope. The results were obtained from three independent experiments of six replicates each.

#### 3.7.5. Anti-PD Activity Using a Mouse Model

Three- to four-month-old male *C57BL/6* mice weighing 25–27 g each were maintained under a constant 12 h light-dark cycle and allowed free access to food and water in a room at 23 °C with a relative humidity of 45%–55%. MPTP was diluted in physiological saline, and compounds **1** and **8** were diluted in less than 0.3% DMSO in saline. All of the injection solutions were freshly prepared before use.

The animals were randomly assigned to 6 groups (each containing 6 mice) consisting of the model group, the control group, two groups for compound **1** (40 mg/kg and 10 mg/kg) and two groups for compound **8** (40 mg/kg and 10 mg/kg). The model group was injected with a 30 mg/kg dose of MPTP daily. The first test groups received intraperitoneal injections of the relevant compounds over 5 days, and the model group received saline. The model and compound-treated groups received subcutaneous MPTP injections for 1 day, intraperitoneal injection for 4 days and another subcutaneous injection on the last day. All of the model and compound-treated groups were injected with saline and the pertinent compounds, respectively, 1 hour before injecting with MPTP. During the treatment schedule, the control group received a saline injection of the same volume. Approximately 12 h after the last injection, the mice were transcardially perfused with 200 mL of a 10% polyformic solution under anesthesia. The brain tissues were immediately collected and maintained in a 10% polyformic solution for 12 h. These brain tissues were dehydrated in a 20% sucrose solution for 24 h and a 30% sucrose solution for another 24 h. The brain tissue slices were then prepared. For the immunofluorescent staining, the SN tissue slices were incubated with primary antibodies for SN (mouse anti-tyrosine hydroxylase) overnight at 48 °C and in daylight 594-conjugated secondary antibodies (goat anti-mouse *IgG*[*H* + *L*] secondary antibody). The images were captured using a fluorescent microscope equipped with a digital camera and connected to a computer. The number of DA neurons was counted using ImageJ software.

#### 3.7.6. Statistics

All of the statistics were obtained using SPSS Statistics 18 software. The survival analysis used the Kaplan-Meier method. A one-way analysis of variance (ANOVA) followed by a post hoc test was performed to evaluate the effects of different groups.

## 4. Conclusions

In conclusion, the neuroprotective activity of 39 novel xyloketal derivatives and Xyl-B were evaluated using respiratory burst and longevity-extending assays. The results indicated that many of the derivatives had better activity than Xyl-B. The SAR analysis indicated that the benzopyrano pyran derivatives had excellent antioxidative and neuroprotective activities. Because of the importance of oxidative stress to PD, the neuroprotective activities of 15 selected compounds were investigated using an MPP+-induced *C. elegans* PD model. Compounds **1** and **8** showed the highest neuroprotective activity based on the worm survival rates of 77% and 81%, respectively, for these compounds. In the mouse PD model, compounds **1** and **8** further demonstrated notable protective actions. In addition, these compounds were easily synthesized in excellent yield. These exciting results provide a new perspective on the neuroprotective activity of xyloketals.

## Abbreviations

PD: Parkinson’s disease
DA: dopaminergic
*C. elegans*: *Caenorhabditis elegans*
ROS: reactive oxygen species
PMA: phorbol myristate acetate
DCF: dichlorofluorescein
DMSO: dimethyl sulfoxide
H_2_DCFDA: 2′,7′-dihydrodichlorofluorescein diacetate
DCM: dichloromethane
MPP+: 1-methyl-4-phenylpyridinium
MPTP: 1-methyl-4-phenyl-1,2,3,6-tetrahydropyridine
hpf: hours post-fertilization
BOP: (benzotriazol-1-yloxy)tris(dimethylamino)phosphonium hexafluorophosphate
DIEA: *N*,*N*-diisopropylethylamine
GFP: green fluorescent protein
NADPH: triphosphopyridine nucleotide
ex.: excitation
em.: emission
ADE: anterior deirid
CEP: cephalic
LSD: Fisher’s least significant difference
EI: electron ionization
HR: high resolution
calcd.: calculated
CCD: charge-coupled-device

## Supplementary Files

Supplementary File 1 Supplementary Information (PDF, 4553 KB)
