# Supplementary material for: Synthesis and Neuroprotective Action of Xyloketal Derivatives in Parkinson’s Disease Models
Source: Mar Drugs. 2013 Dec 18;11(12):5159–89. doi: 10.3390/md11125159 (PMC3877910; doi:10.3390/md11125159)
Supplement: Supplementary File 1 — Supplementary Information (PDF, 4553 KB) [file marinedrugs-11-05159-s001.pdf]

Supplementary Information

Figure S1. ^{1}H NMR ($CDCl_3$, 400 MHz) of **6**.
Figure S2. ^{13}C NMR ($CDCl_3$, 101 MHz) of **6**.
Figure S3. ^{1}H NMR ($CDCl_3$, 400 MHz) of (±)-**6a**.
Figure S4. ^{13}C NMR ($CDCl_3$, 101 MHz) of (±)-**6a**.
Figure S5. ^{13}C NMR and DEPT spectra ($CDCl_3$, 101 MHz) of (±)-**6a**.
Figure S6. H-H COSY of compound (±)-**6a**.
Figure S7. HSQC spectrum of compound (±)-**6a**.
Figure S8. HMBC spectrum of compound (±)-**6a**.
Figure S9. NOE spectrum of compound (±)-**6a**.
Figure S10. ^{1}H NMR ($CDCl_3$, 400 MHz) of (±)-**6b**.
Figure S11. ^{13}C NMR ($CDCl_3$, 101 MHz) of (±)-**6b**.
Figure S12. ^{13}C NMR and DEPT spectra ($CDCl_3$, 101 MHz) of (±)-**6b**.
Figure S13. H-H COSY of (±)-**6b**.
Figure S14. HSQC spectrum of (±)-**6b**.
Figure S15. HMBC spectrum of (±)-**6b**.
Figure S16. NOE spectrum of (±)-**6b**.
Figure S17. ^{1}H NMR ($CDCl_3$, 400 MHz) of **1**.
Figure S18. ^{13}C NMR ($CDCl_3$, 101 MHz) of **1**.
Figure S19. H-H COSY of **1**.
Figure S20. HSQC spectrum of **1**.
Figure S21. HMBC spectrum of **1**.
Figure S22. ^{1}H NMR ($CDCl_3$, 400 MHz) of **2**.
Figure S23. ^{13}C NMR ($CDCl_3$, 101 MHz) of **2**.
Figure S24. ^{1}H NMR ($CDCl_3$, 400 MHz) of **3**.
Figure S25. ^{13}C NMR ($CDCl_3$, 101 MHz) of **3**.
Figure S26. ^{1}H NMR ($CDCl_3$, 400 MHz) of **4**.
Figure S27. ^{13}C NMR ($CDCl_3$, 101 MHz) of **4**.
Figure S28. ^{1}H NMR ($CDCl_3$, 400 MHz) of **5**.
Figure S29. ^{13}C NMR ($CDCl_3$, 101 MHz) of **5**.
Figure S30. ^{1}H NMR ($CDCl_3$, 400 MHz) of **7**.
Figure S31. ^{13}C NMR ($CDCl_3$, 101 MHz) of **7**.
Figure S32. ^{1}H NMR ($CDCl_3$, 400 MHz) of **8**.
Figure S33. ^{1}H NMR ($CDCl_3$, 400 MHz) of **9**.
Figure S34. ^{13}C NMR ($CDCl_3$, 101 MHz) of **9**.
Figure S35. ^{1}H NMR ($CDCl_3$, 400 MHz) of **20**.
Figure S36. ^{13}C NMR ($CDCl_3$, 101 MHz) of **20**.
Figure S37. ^{1}H NMR ($CDCl_3$, 400 MHz) of **21**.
Figure S38. ^{13}C NMR ($CDCl_3$, 101 MHz) of **21**.
Figure S39. ^{1}H NMR ($CDCl_3$, 400 MHz) of **26**.
Figure S40. ^{13}C NMR ($CDCl_3$, 101 MHz) of **26**.
Figure S41. ^{1}H NMR ($CDCl_3$, 400 MHz) of **31**.

Figure S42. ^{13}C NMR ($CDCl_3$, 101 MHz) of **31**.
Figure S43. ^{1}H NMR ($CDCl_3$, 400 MHz) of **39**.
Figure S44. ^{13}C NMR ($CDCl_3$, 101 MHz) of **39**.
Figure S45. HPLC chromatography and elution conditions of representative compounds.
Figure S46. LC-MS of compound **6** under elution condition A (0.1% acetic acid was added).
Figure S47. LC-MS of compound **23** using a gradient from 50% to 20% H_2O over 60 min at 1 mL/min.
Figure S48. X-ray diffraction data for (±)-**6a**.
Table S1. NMR spectral data for (±)-**6a** and (±)-**6b** ($CDCl_3$) (δ in ppm, *J* in Hz).
Table S2. Purity and retention times of all tested compounds.
Table S3. Crystal data and structure refinement for (±)-**6a**.
Table S4. Atomic coordinates ($\times 10^4$) and equivalent isotropic displacement parameters ($Å^2 \times 10^3$) for (±)-**6a**.
Table S5. Bond lengths (Å) and angles (deg.) for (±)-**6a**.
Table S6. Anisotropic displacement parameters ($Å^2 \times 10^3$) for (±)-**6a**.
Table S7. Hydrogen coordinates ($\times 10^4$) and isotropic displacement parameters ($Å^2 \times 10^3$) for (±)-**6a**.
Table S8. Torsion angles (deg.) for (±)-**6a**.
Table S9. Hydrogen bonds for (±)-**6a** (Å and deg.).

Figure S1. ^{1}H NMR ($CDCl_3$, 400 MHz) of **6**.

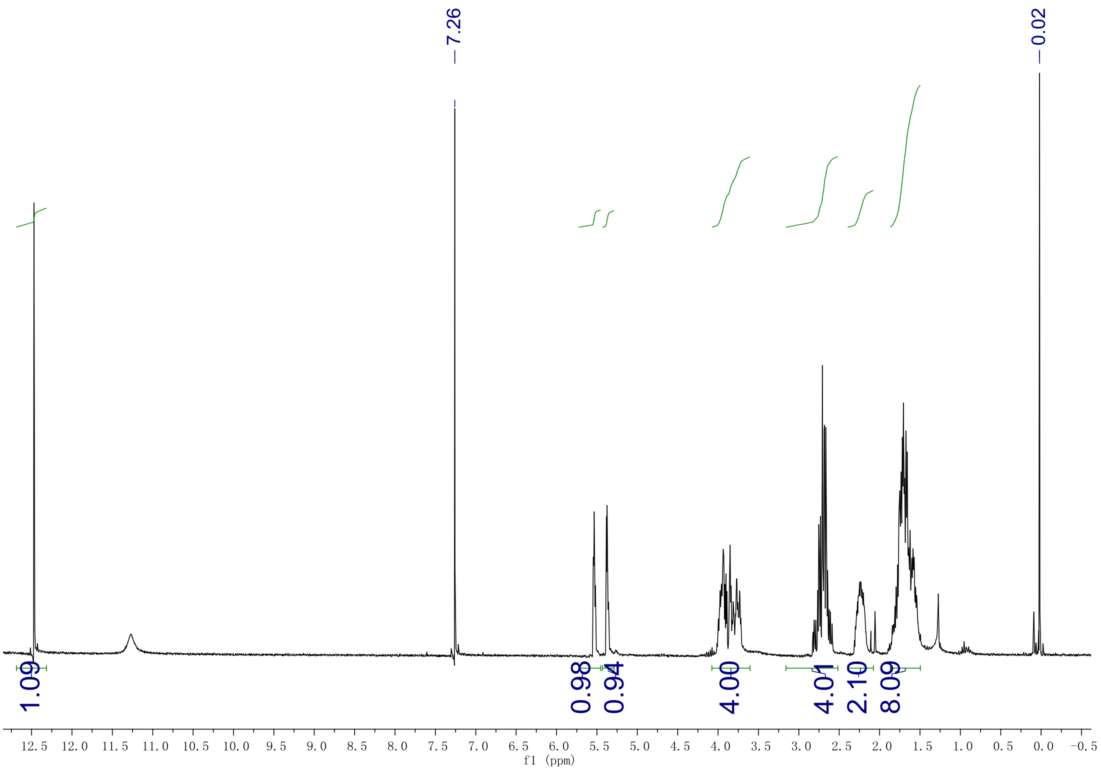

Figure S2. ^{13}C NMR ($CDCl_3$, 101 MHz) of **6**.

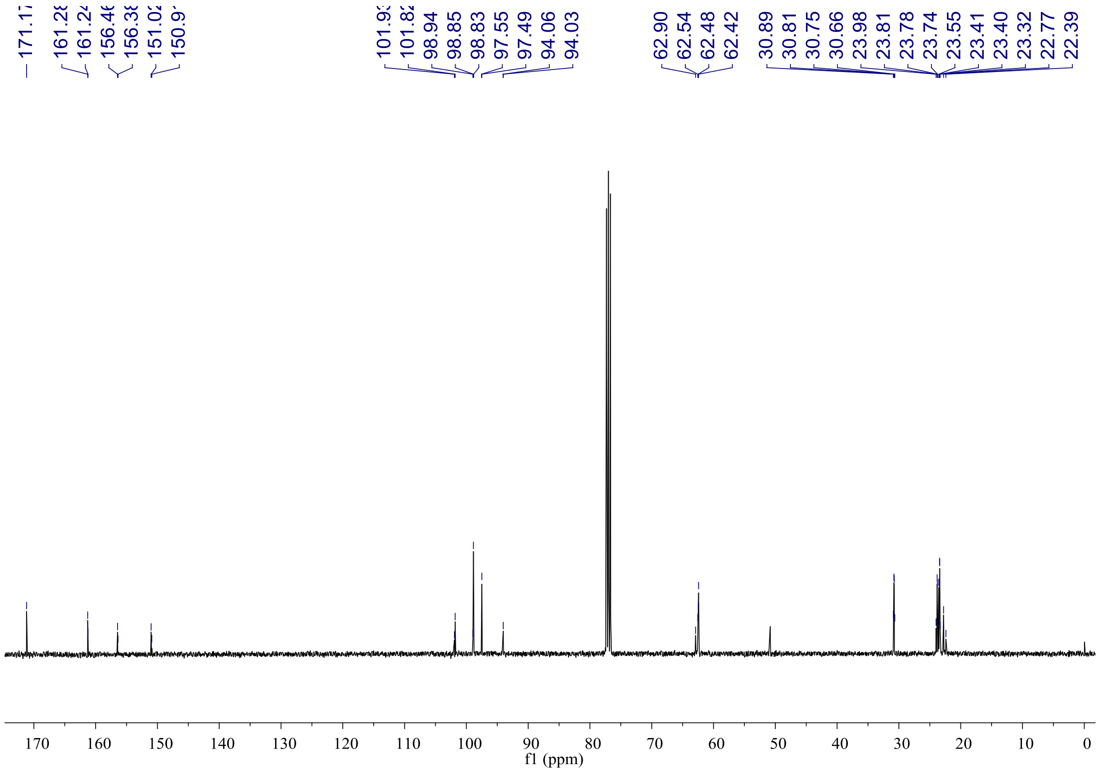

Figure S3. ^{1}H NMR ($CDCl_3$, 400 MHz) of (±)-**6a**.

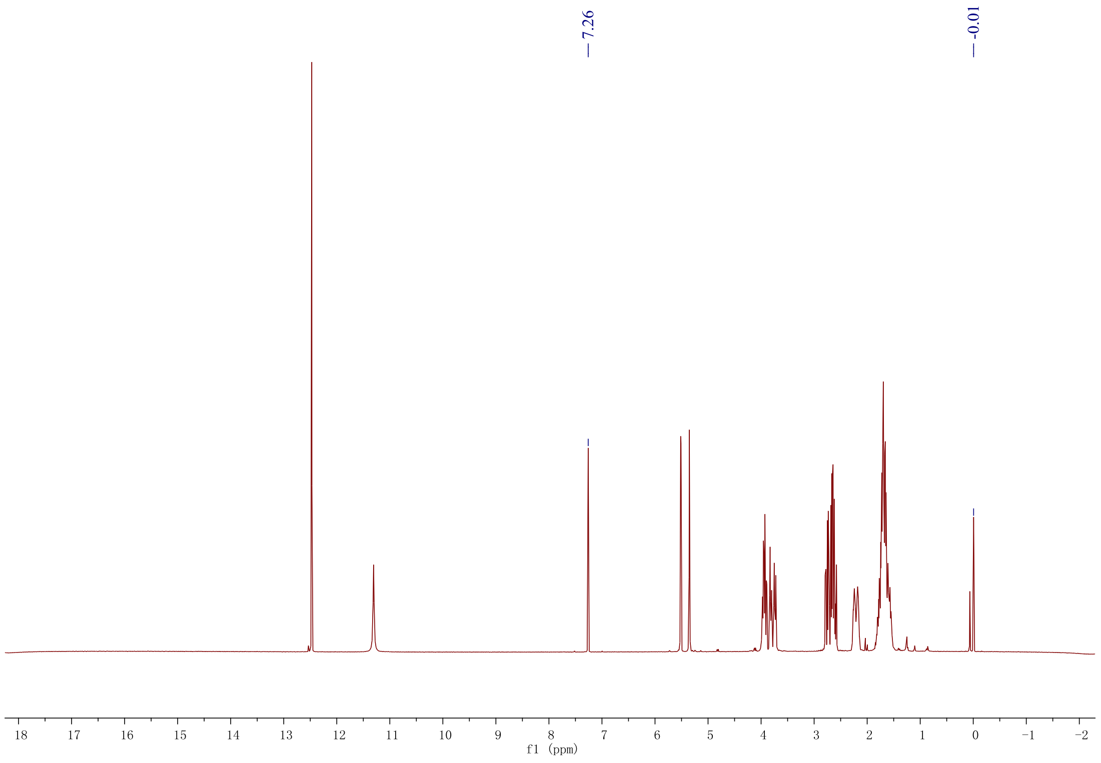

Figure S4. ^{13}C NMR ($CDCl_3$, 101 MHz) of (±)-**6a**.

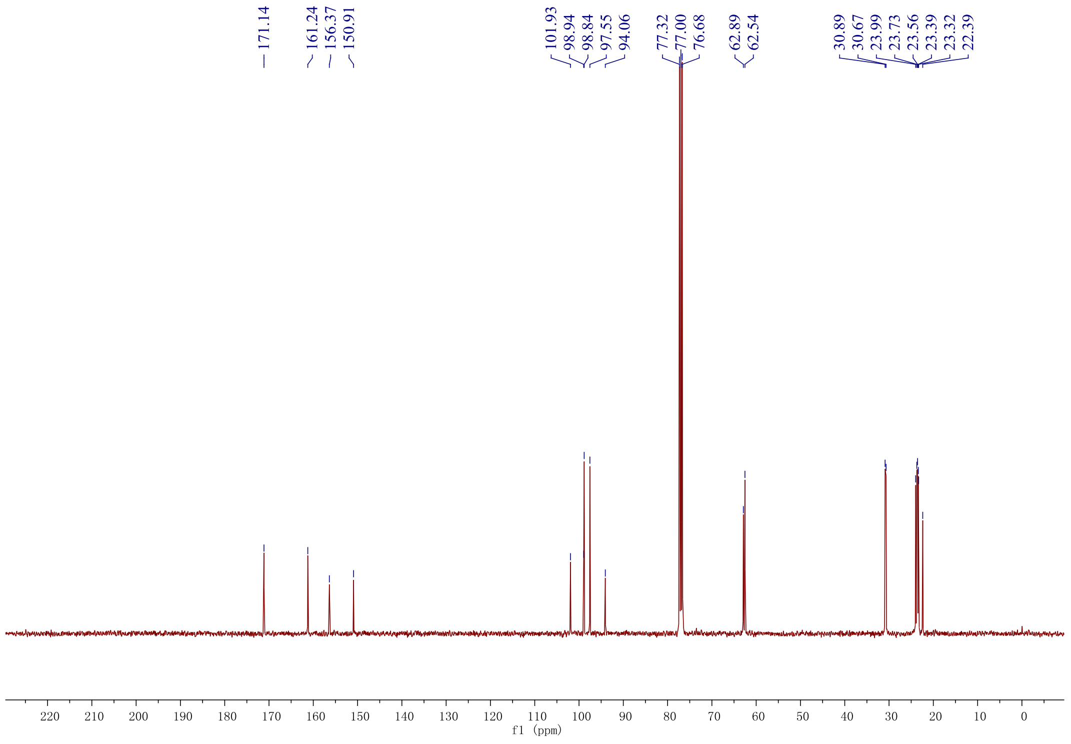

Figure S5. ^{13}C NMR and DEPT spectra ($CDCl_3$, 101 MHz) of (±)-**6a**.

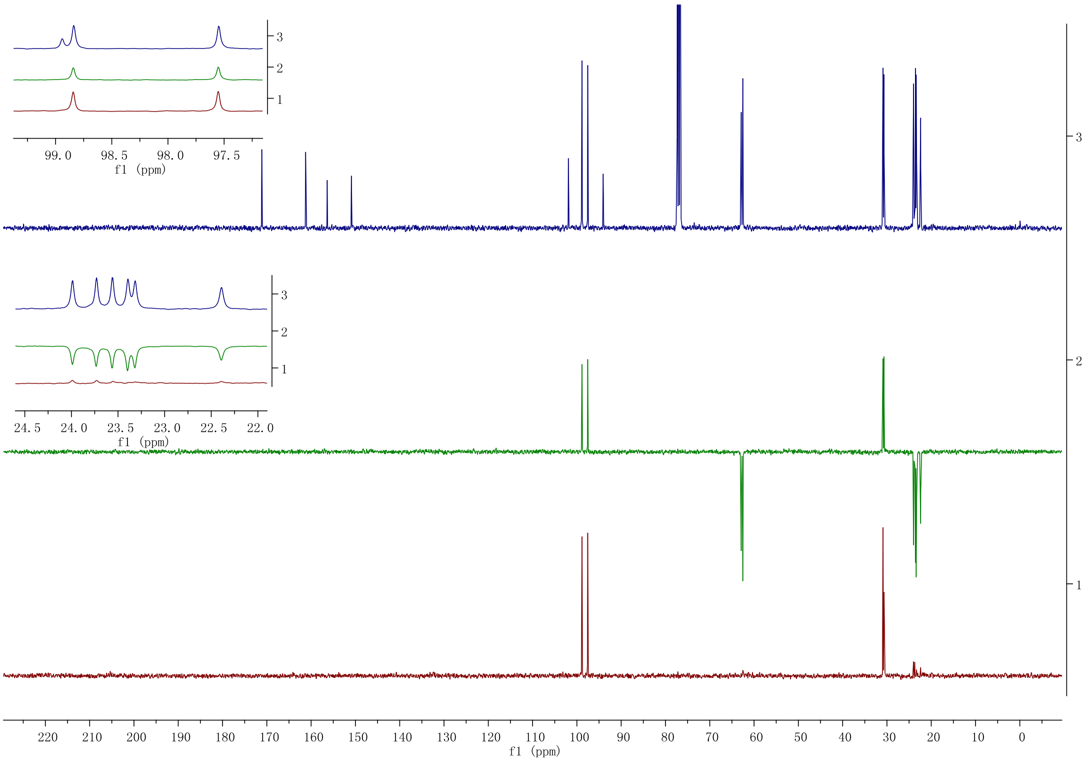

Figure S6. H-H COSY of compound (±)-**6a**.

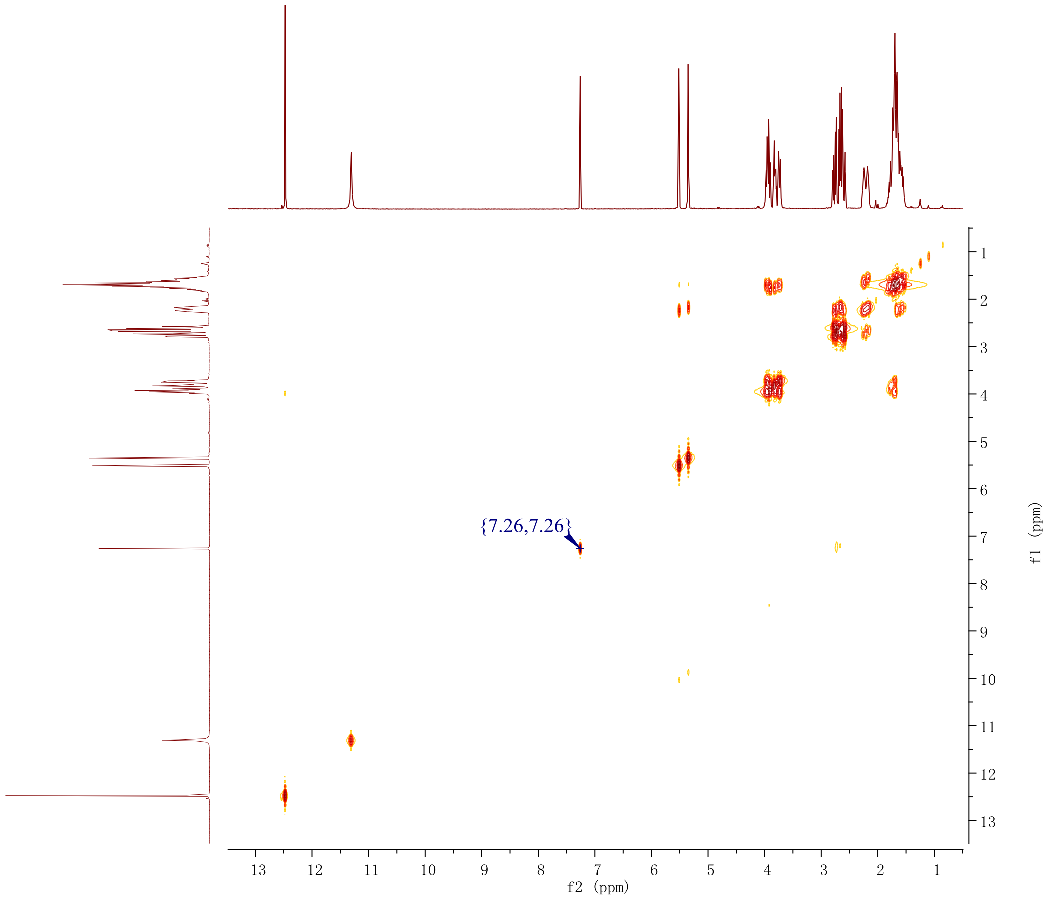

Figure S7. HSQC spectrum of compound (±)-**6a**.

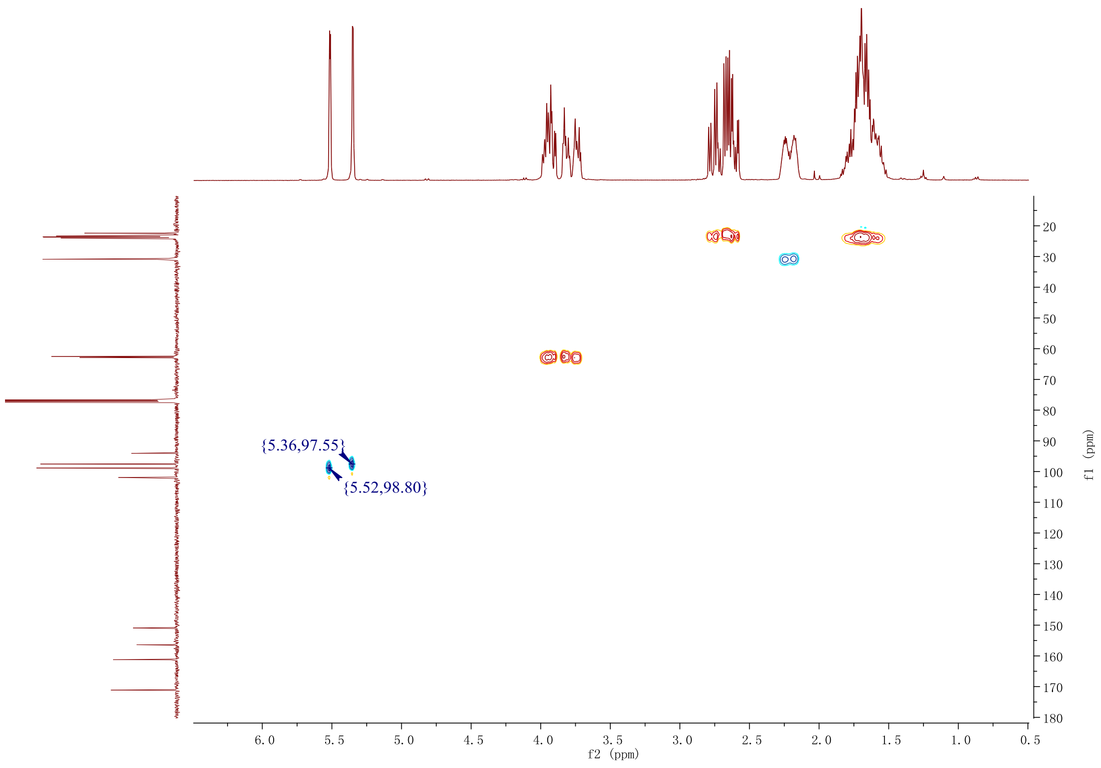

Figure S8. HMBC spectrum of compound (±)-**6a**.

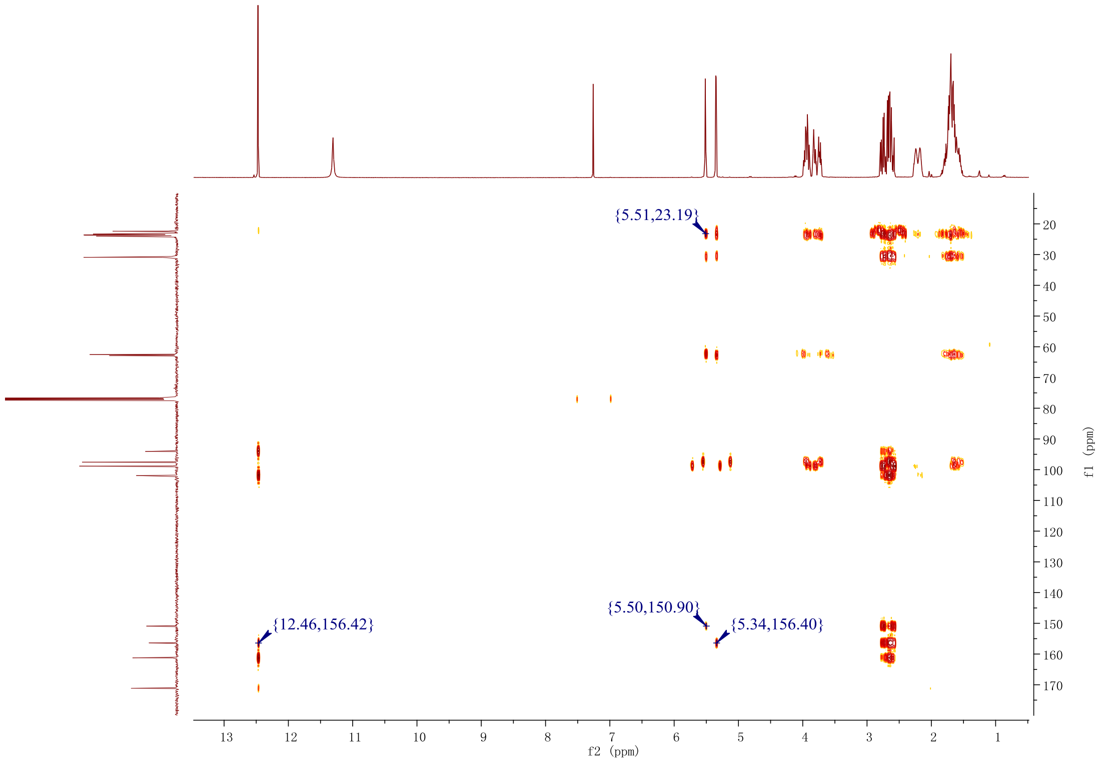

Figure S9. NOE spectrum of compound (±)-**6a**.

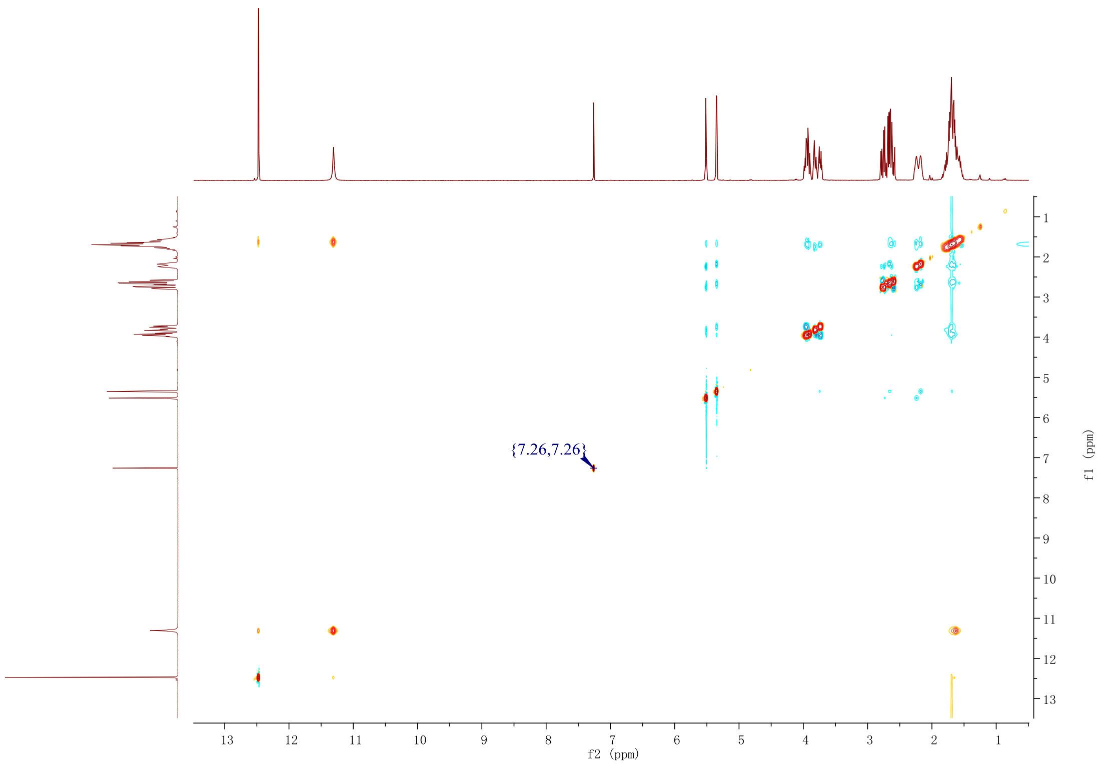

Figure S10. ^{1}H NMR ($CDCl_3$, 400 MHz) of (±)-**6b**.

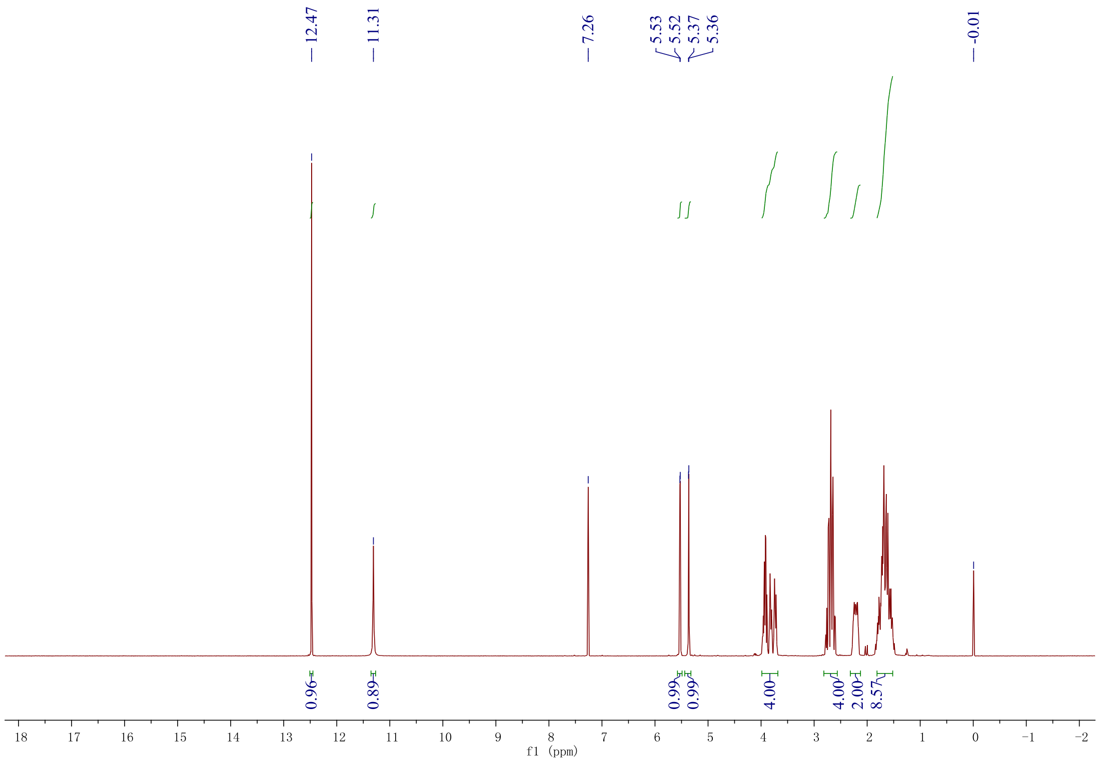

Figure S11. ^{13}C NMR ($CDCl_3$, 101 MHz) of (±)-**6b**.

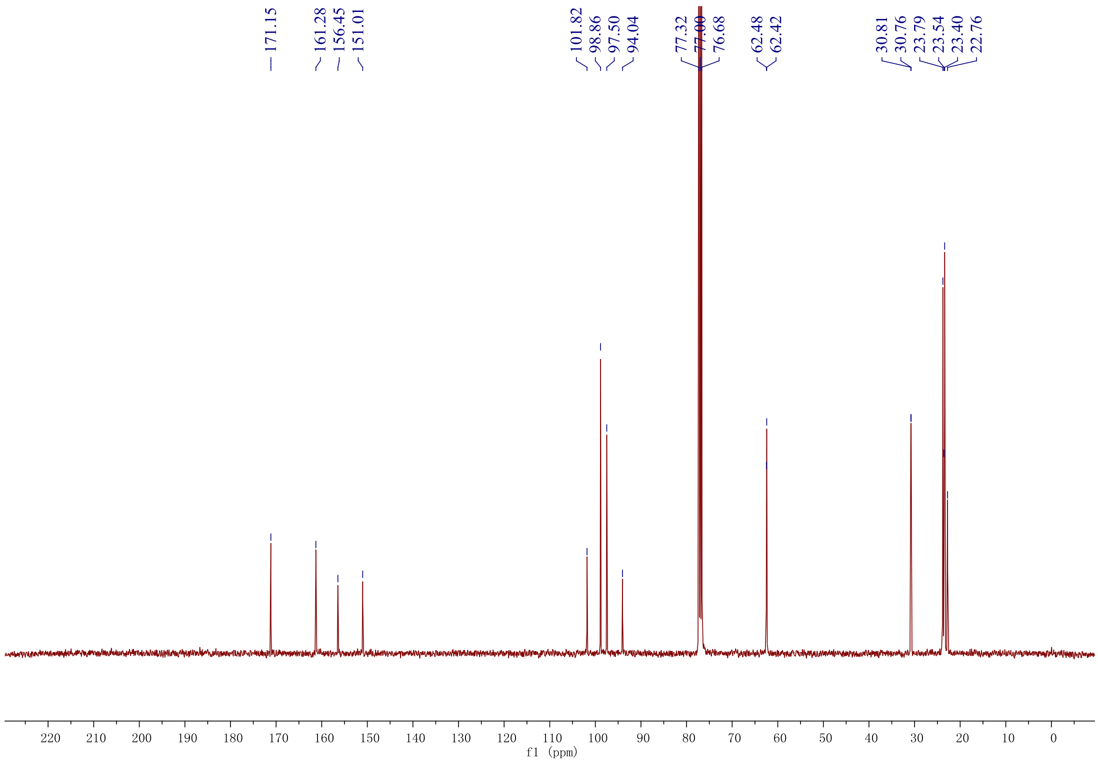

Figure S12. ^{13}C NMR and DEPT spectra ($CDCl_3$, 101 MHz) of (±)-**6b**.

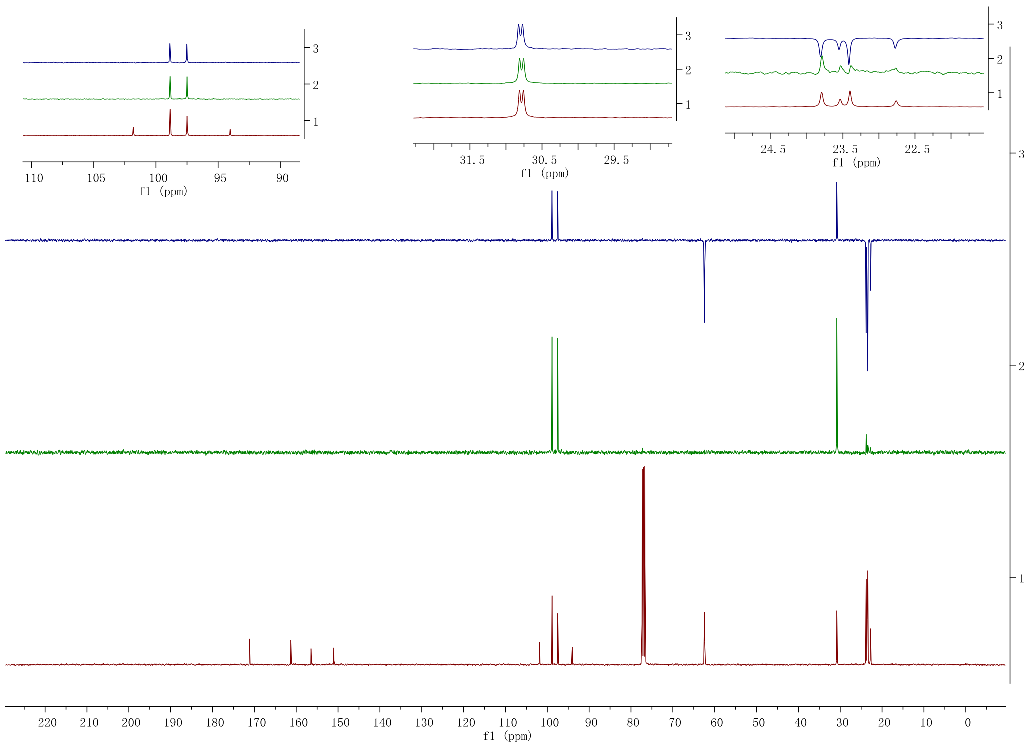

Figure S13. H-H COSY of (±)-**6b**.

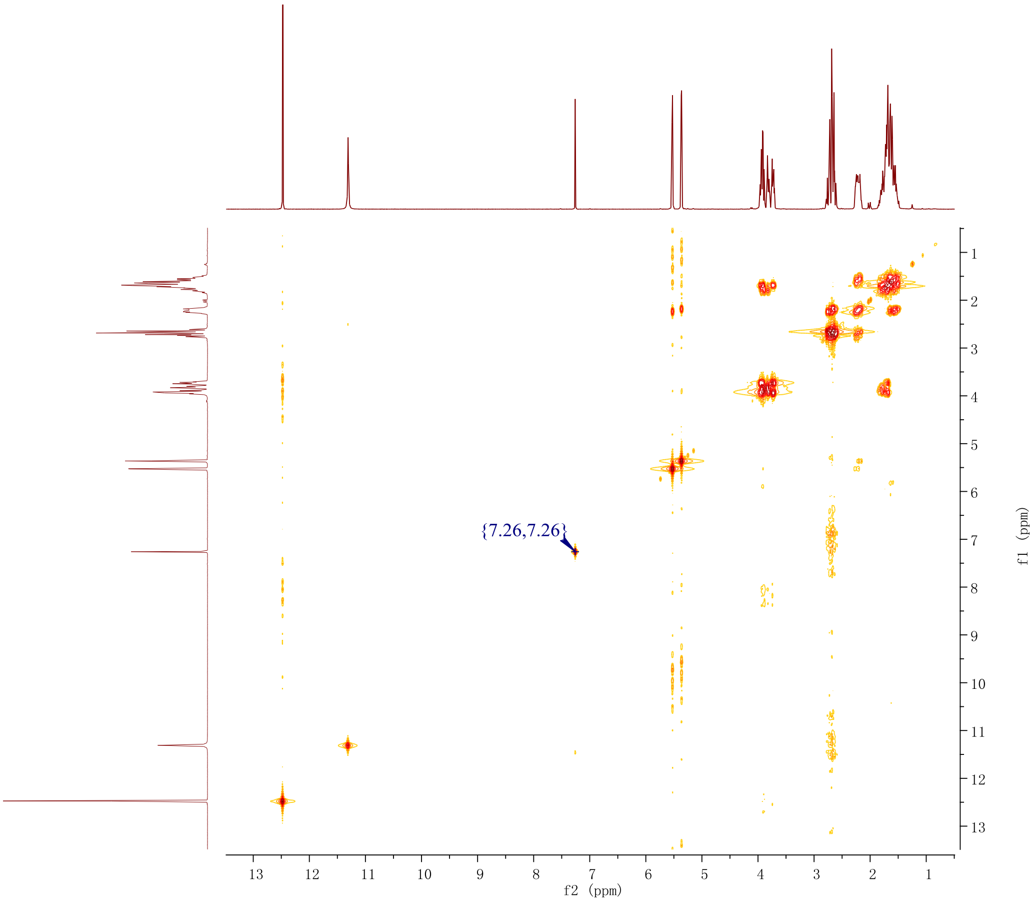

Figure S14. HSQC spectrum of (±)-**6b**.

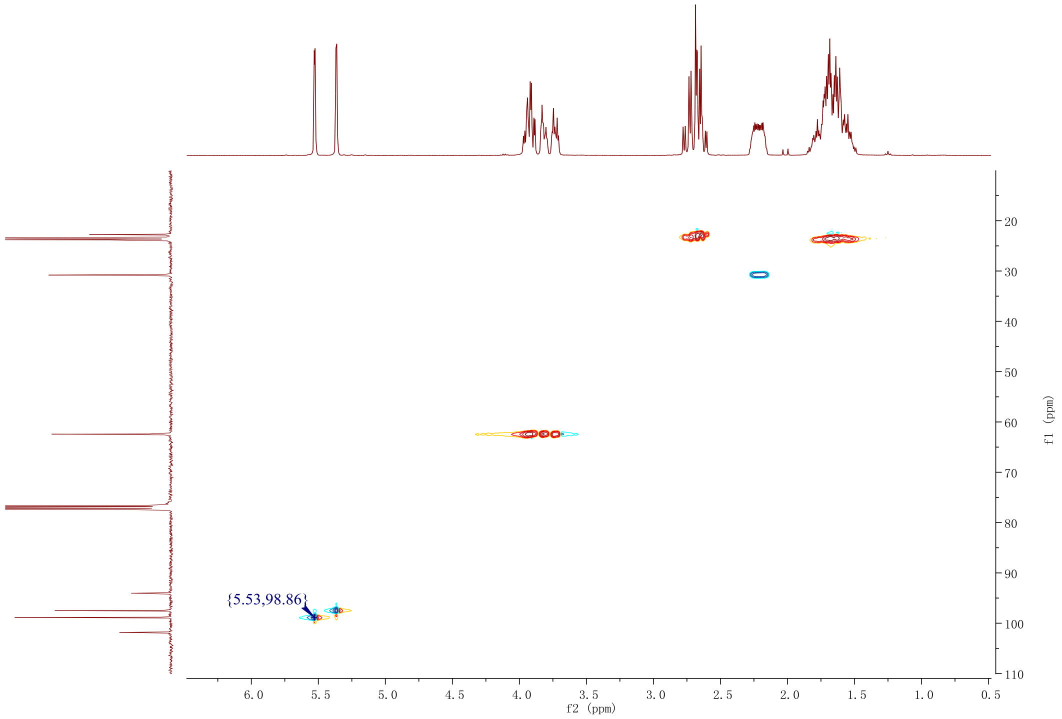

Figure S15. HMBC spectrum of (±)-**6b**.

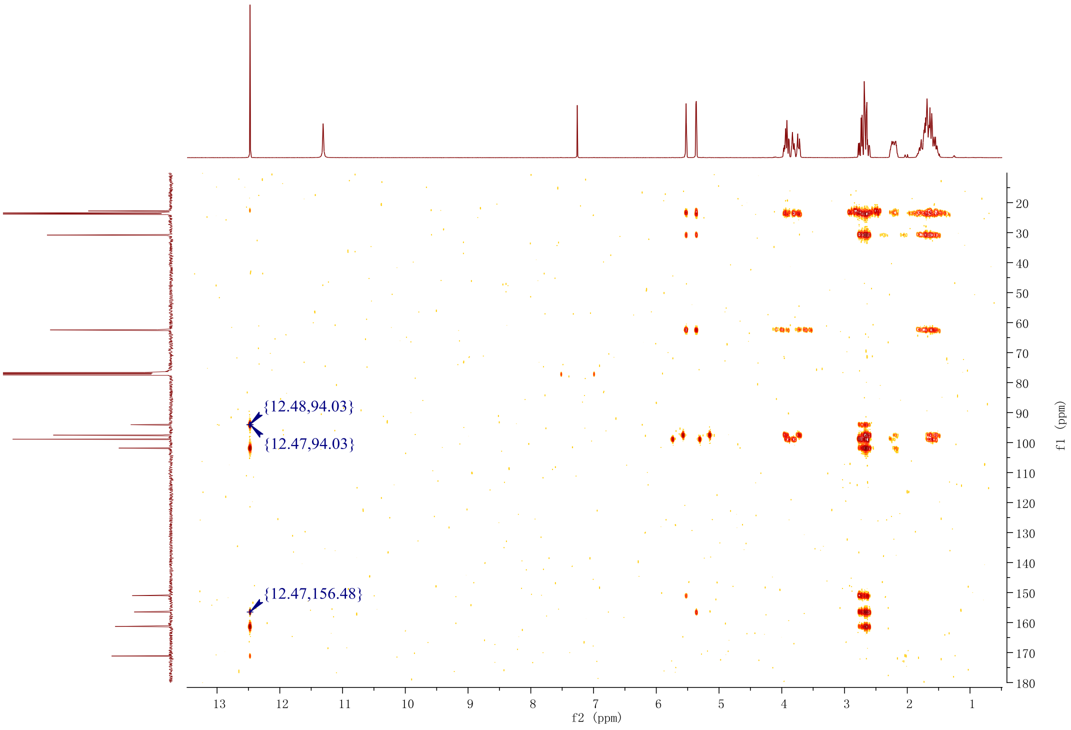

Figure S16. NOE spectrum of (±)-**6b**.

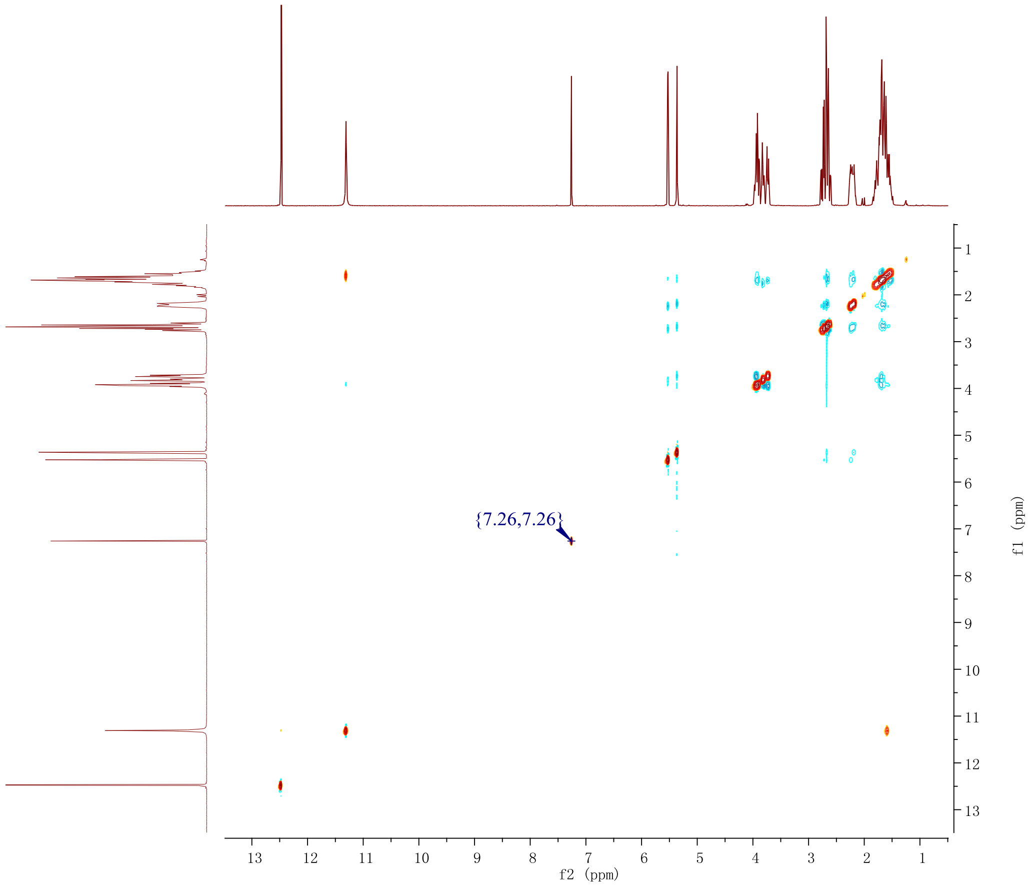

Figure S17. ^{1}H NMR ($CDCl_3$, 400 MHz) of **1**.

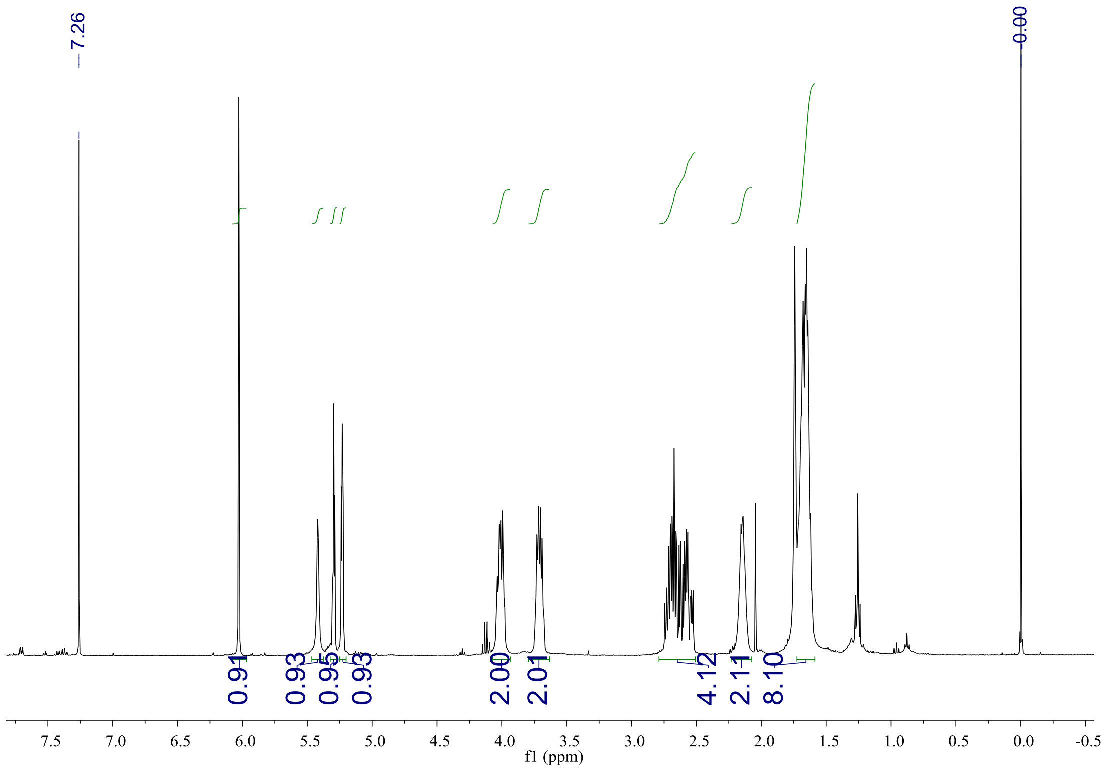

Figure S18. ^{13}C NMR ($CDCl_3$, 101 MHz) of **1**.

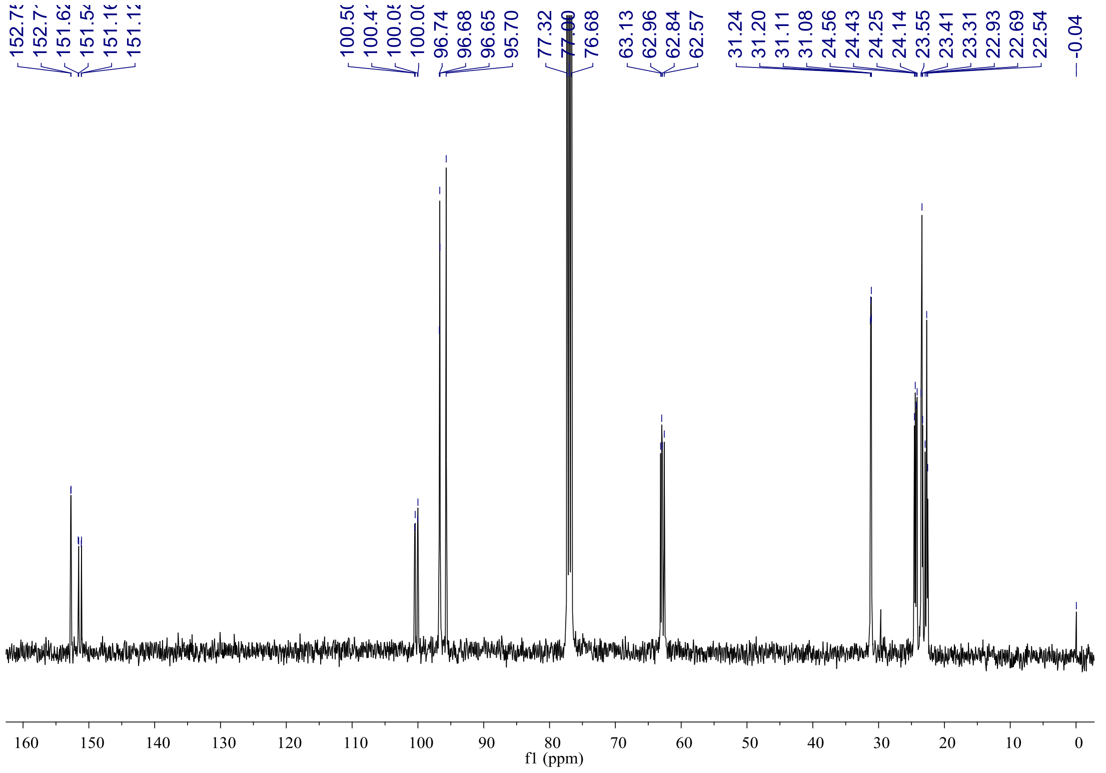

Figure S19. H-H COSY of **1**.

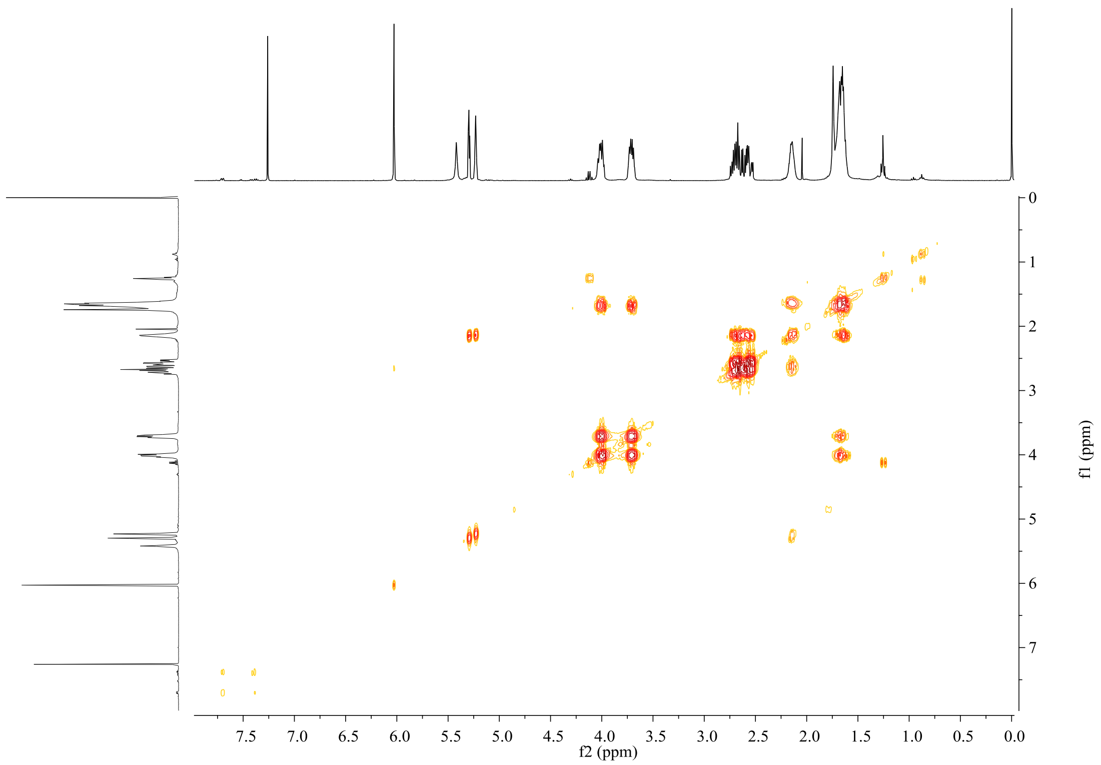

Figure S20. HSQC spectrum of **1**.

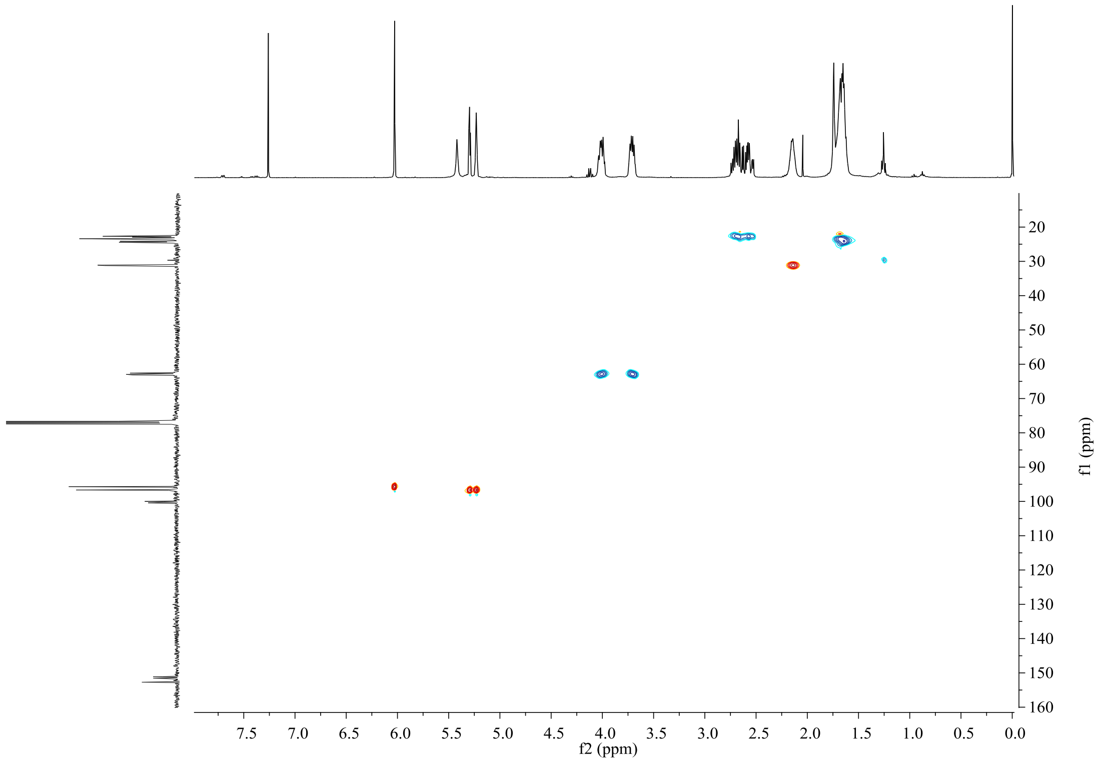

Figure S21. HMBC spectrum of **1**.

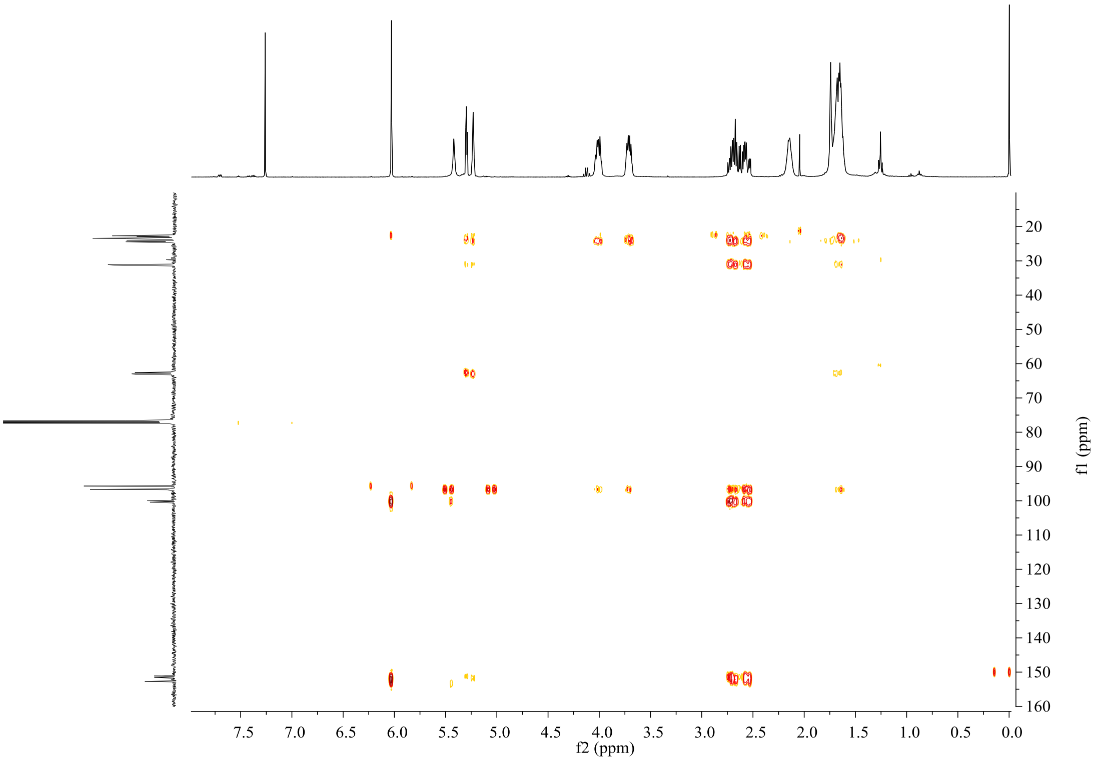

Figure S22. ^{1}H NMR ($CDCl_3$, 400 MHz) of **2**.

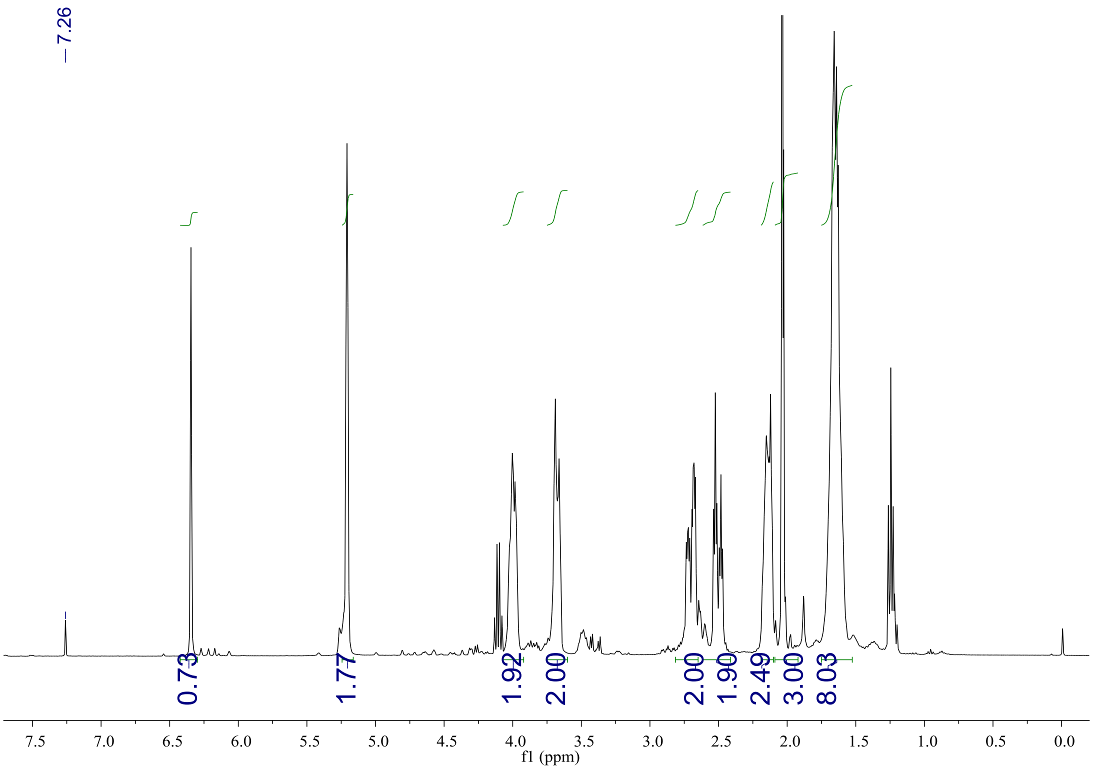

Figure S23. ^{13}C NMR ($CDCl_3$, 101 MHz) of **2**.

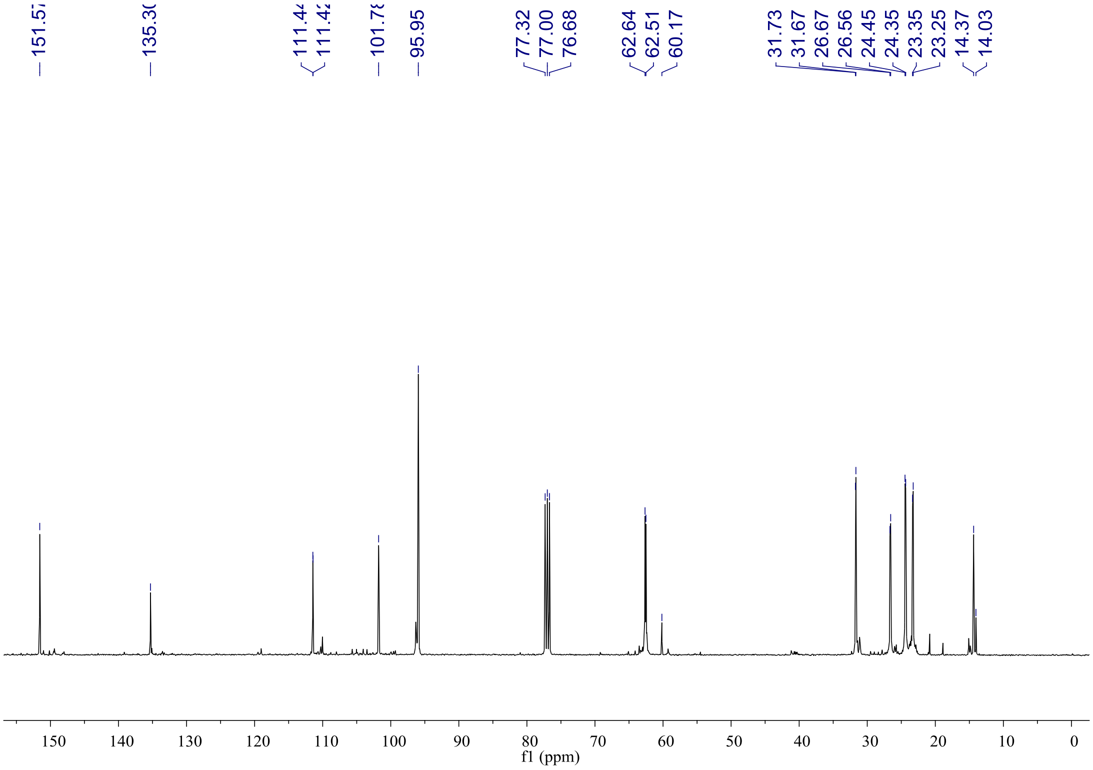

Figure S24. ^{1}H NMR ($CDCl_3$, 400 MHz) of **3**.

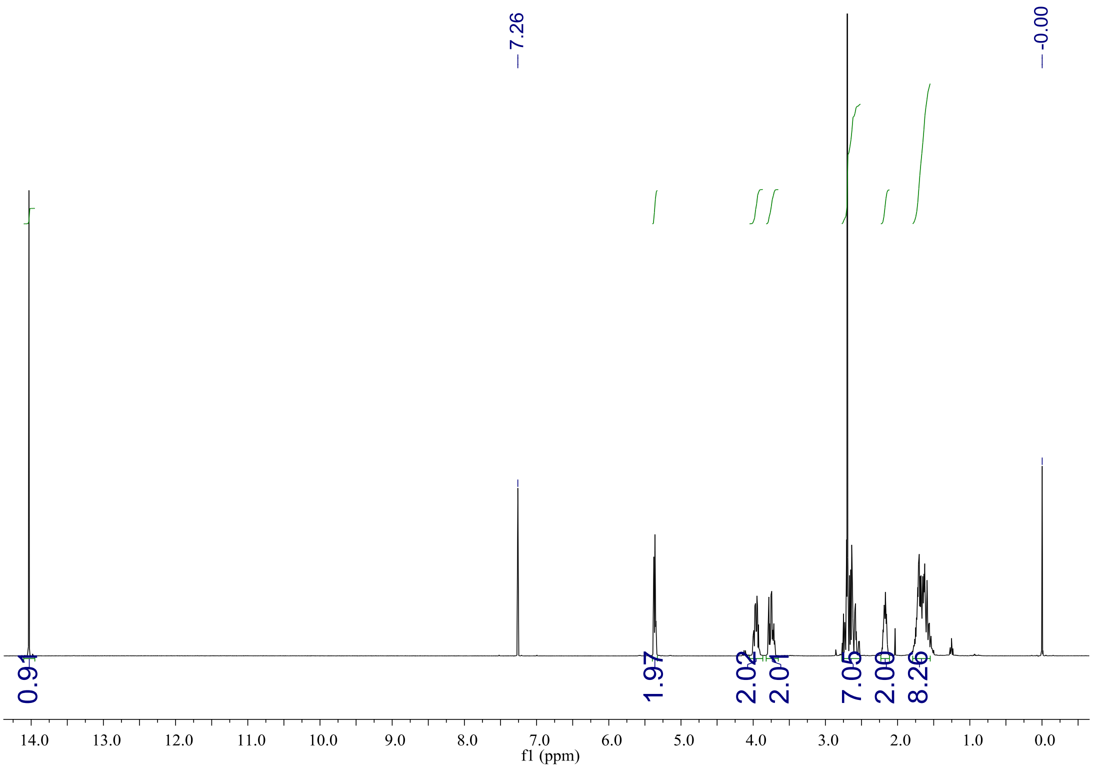

Figure S25. ^{13}C NMR ($CDCl_3$, 101 MHz) of **3**.

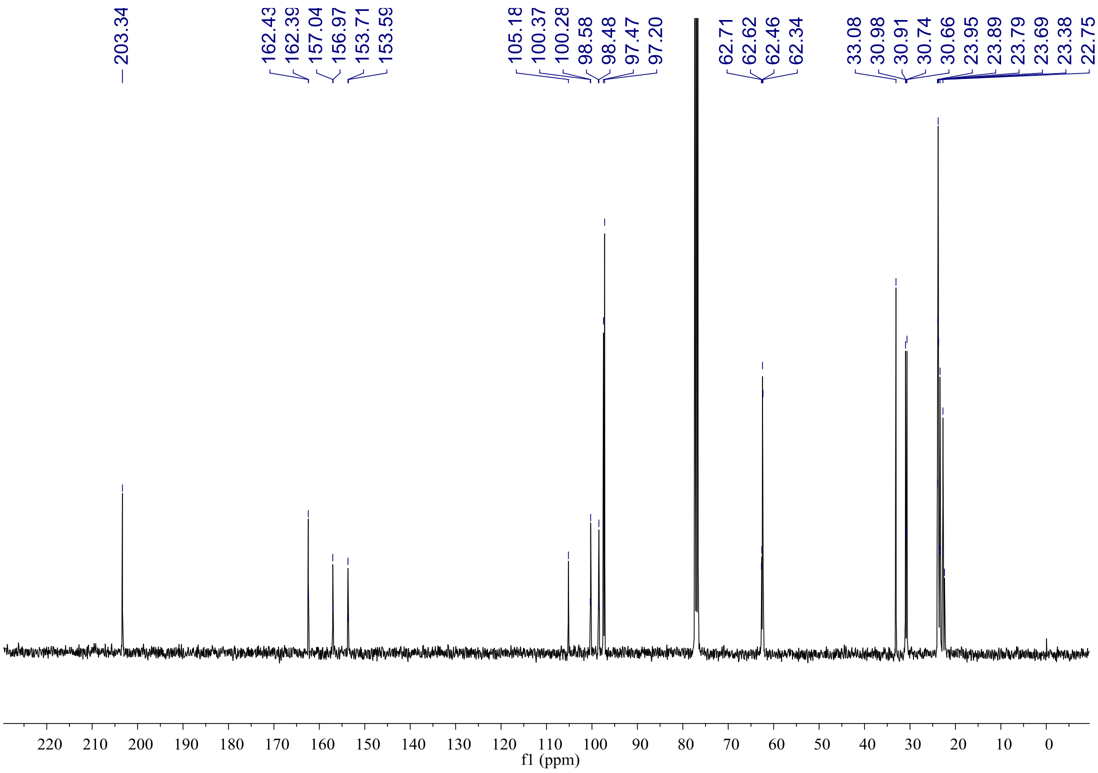

Figure S26. ^{1}H NMR ($CDCl_3$, 400 MHz) of **4**.

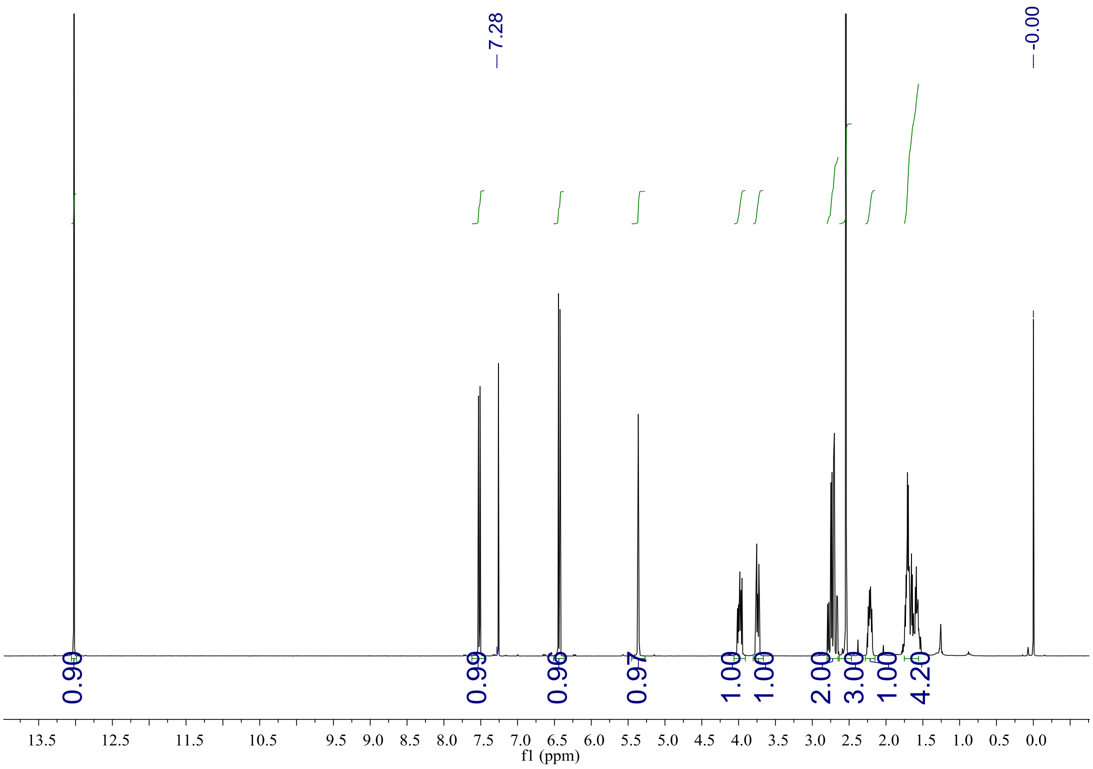

Figure S27. ^{13}C NMR ($CDCl_3$, 101 MHz) of **4**.

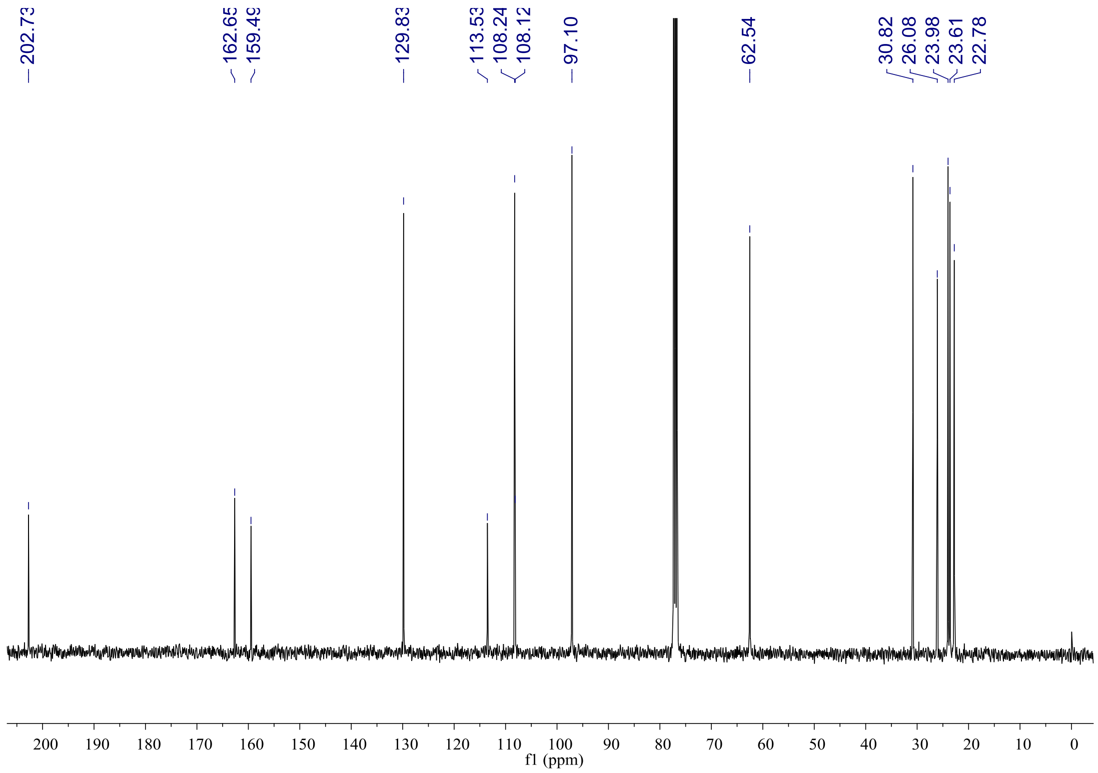

Figure S28. ^{1}H NMR ($CDCl_3$, 400 MHz) of **5**.

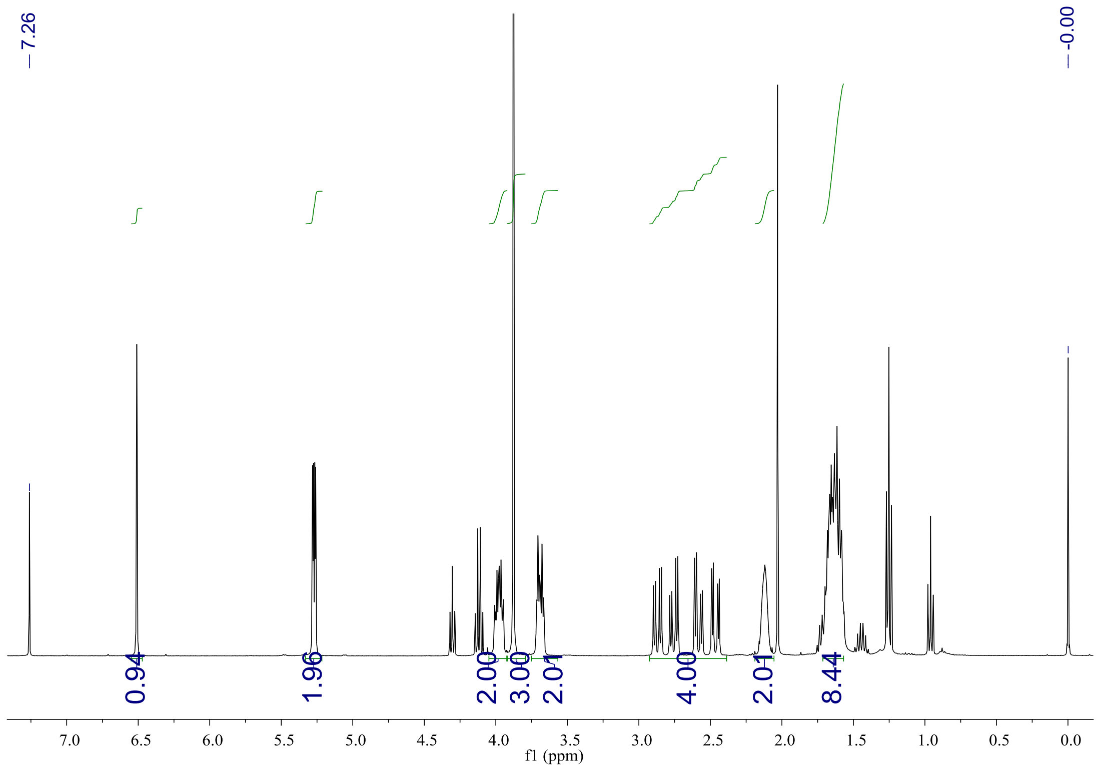

Figure S29. ^{13}C NMR ($CDCl_3$, 101 MHz) of **5**.

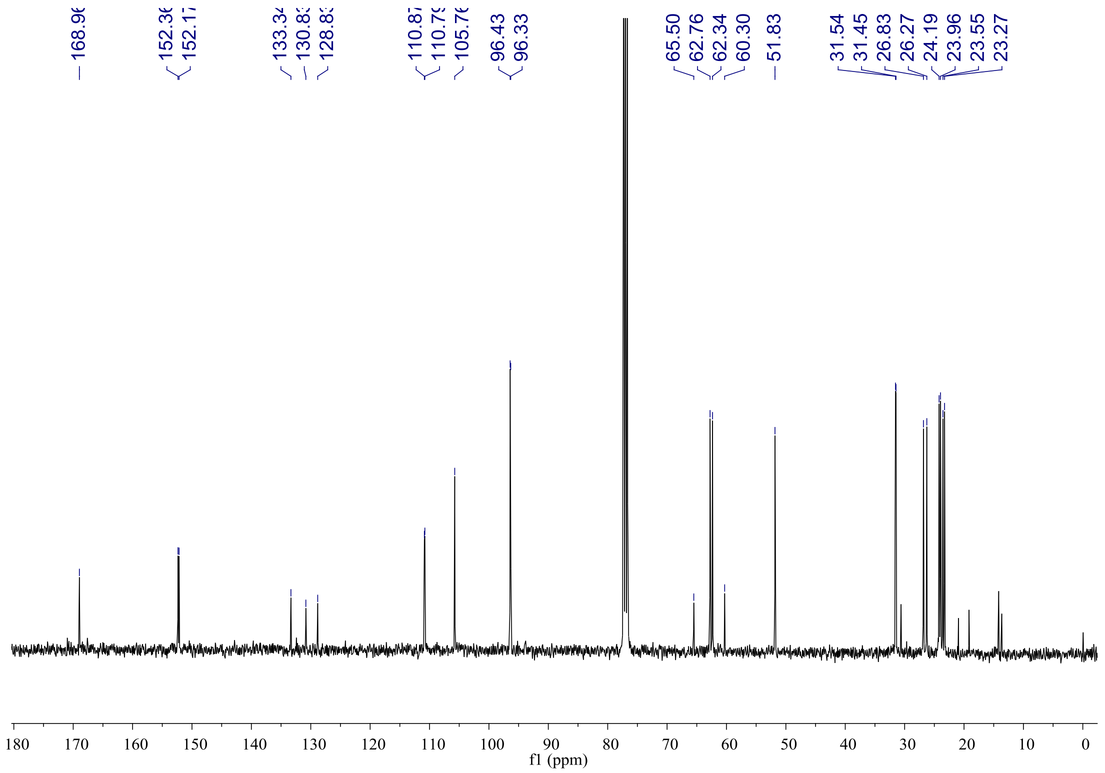

Figure S30. ^{1}H NMR ($CDCl_3$, 400 MHz) of **7**.

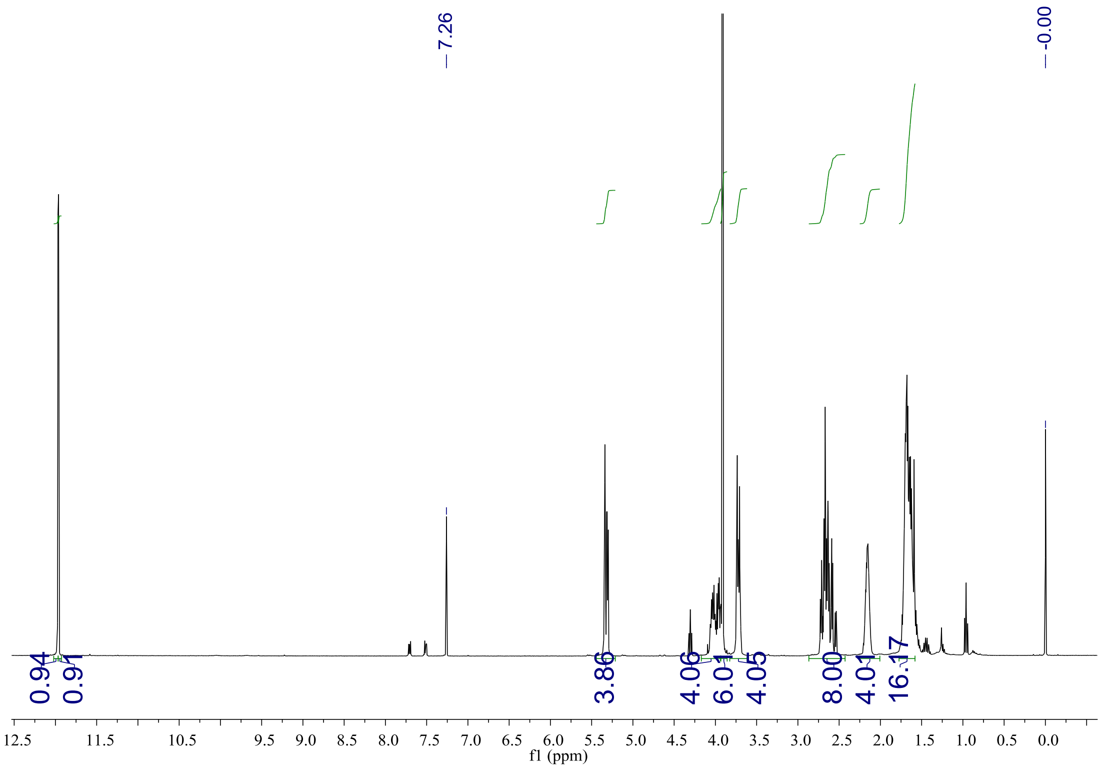

Figure S31. ^{13}C NMR ($CDCl_3$, 101 MHz) of **7**.

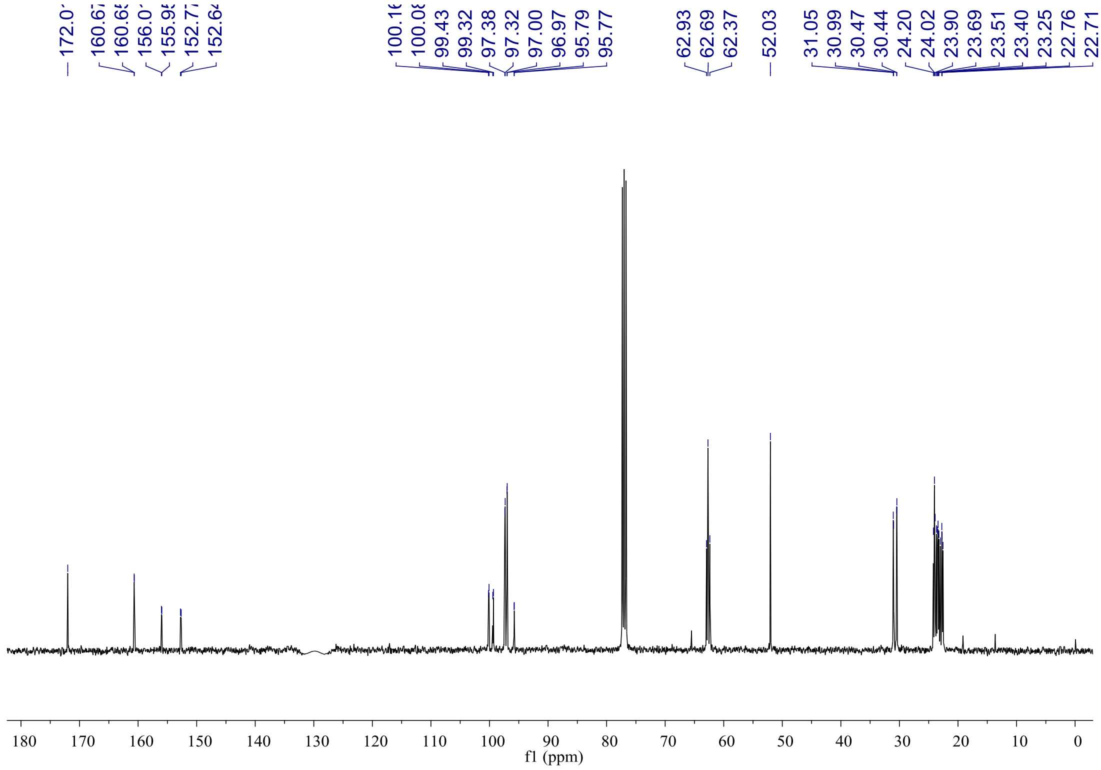

Figure S32. ^{1}H NMR ($CDCl_3$, 400 MHz) of **8**.

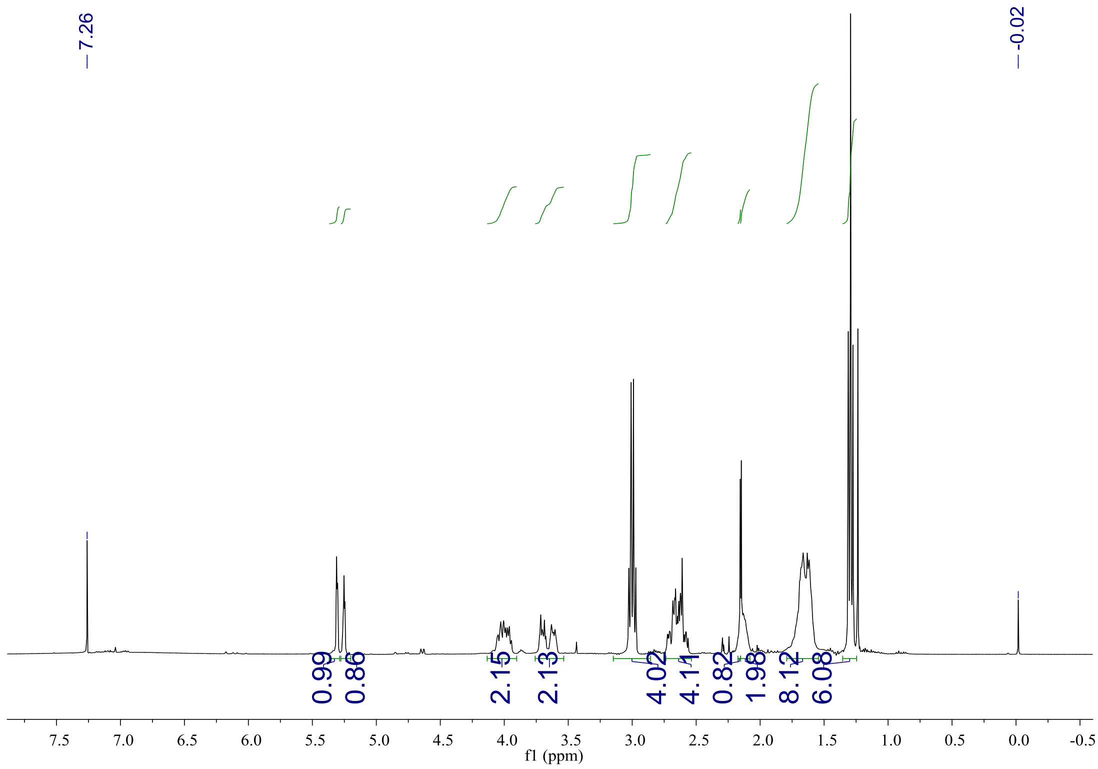

Figure S33. ^{1}H NMR ($CDCl_3$, 400 MHz) of **9**.

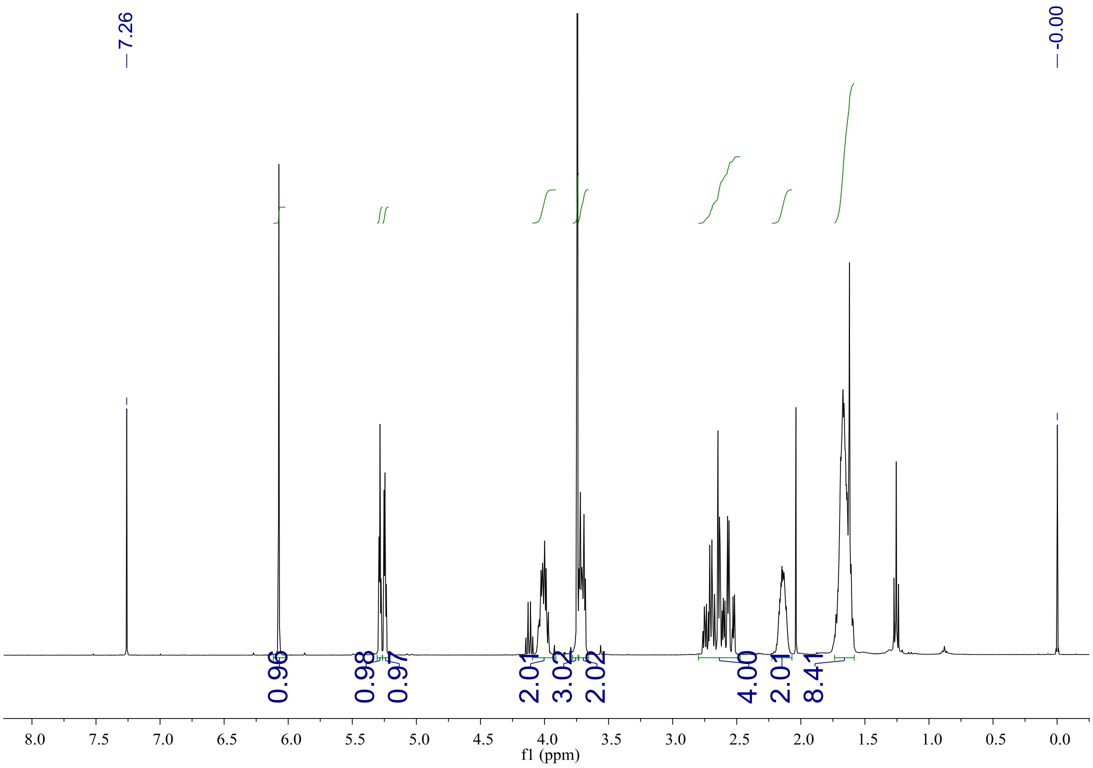

Figure S34. ^{13}C NMR ($CDCl_3$, 101 MHz) of **9**.

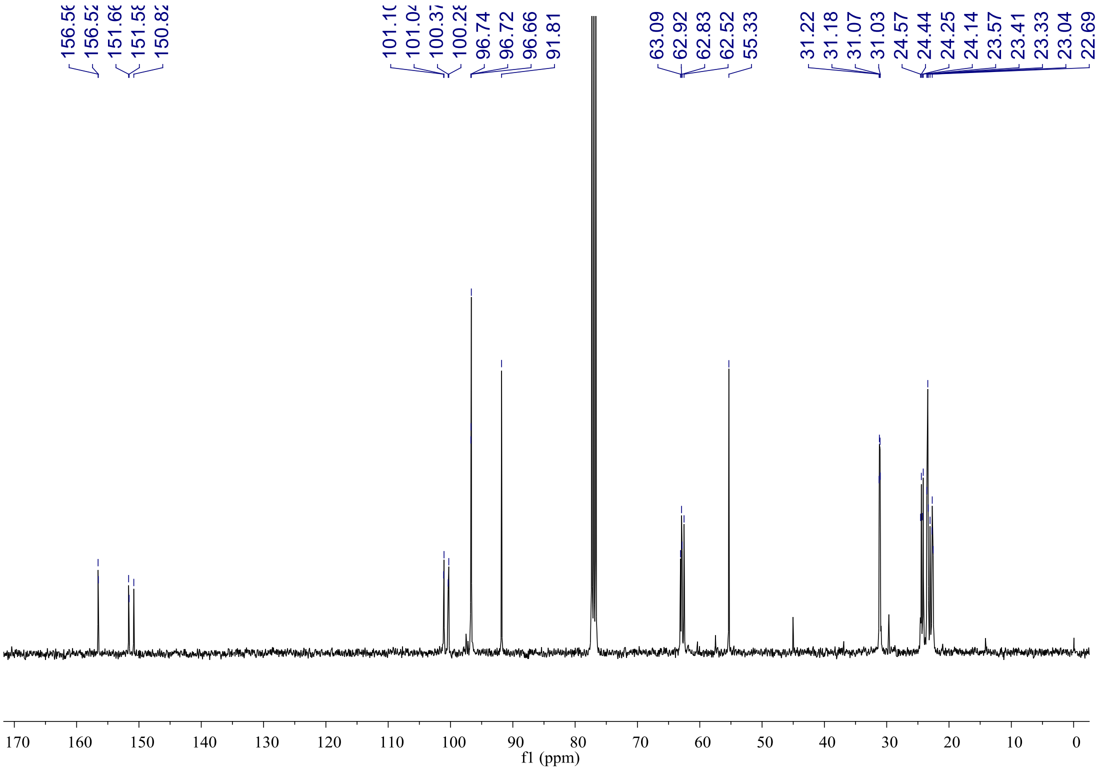

Figure S35. ^{1}H NMR ($CDCl_3$, 400 MHz) of **20**.

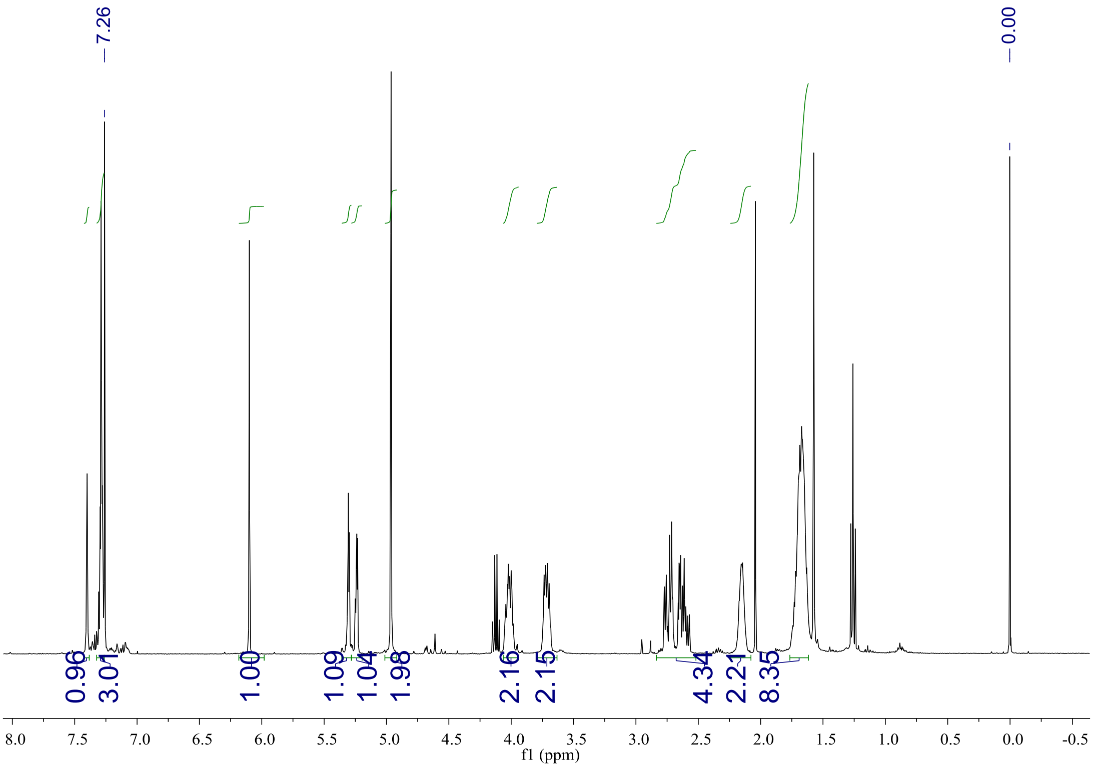

Figure S36. ^{13}C NMR ($CDCl_3$, 101 MHz) of **20**.

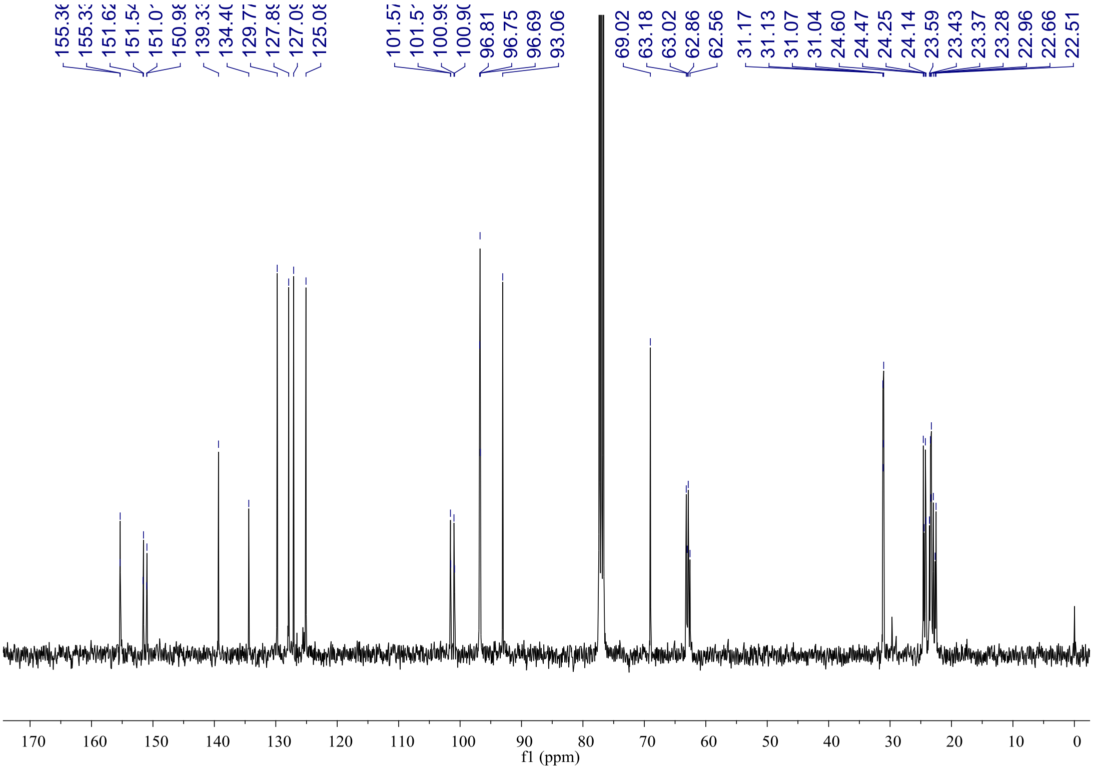

Figure S37. ^{1}H NMR ($CDCl_3$, 400 MHz) of **21**.

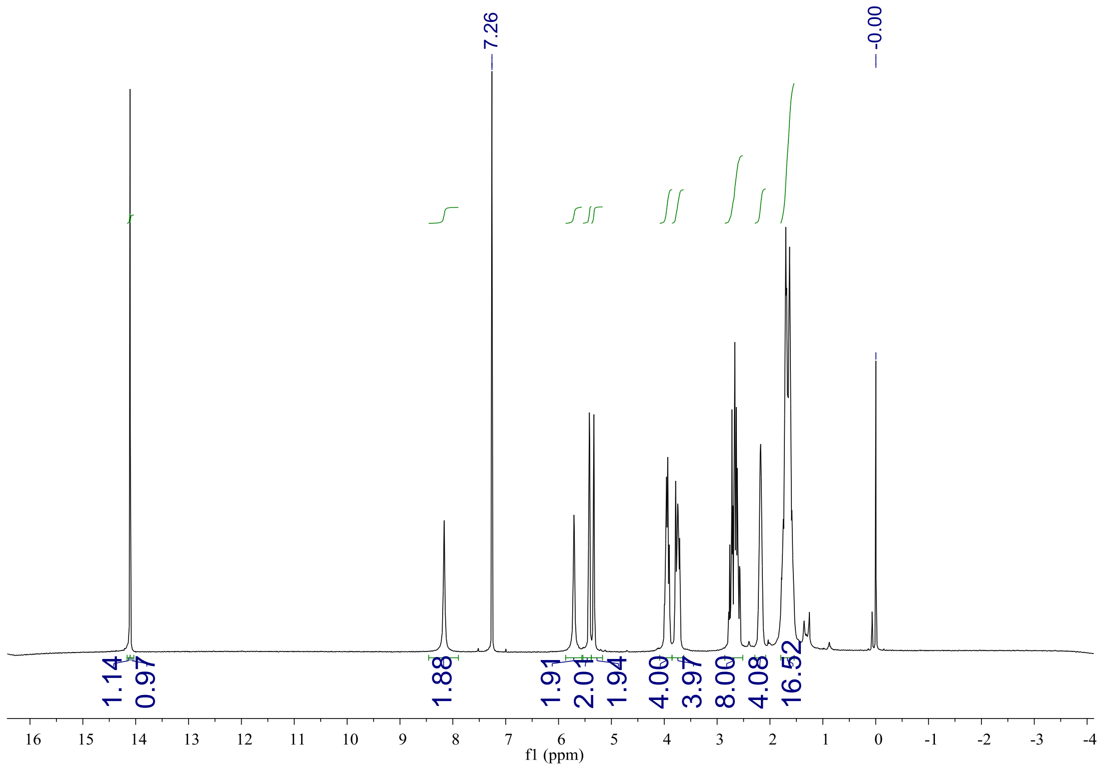

Figure S38. ^{13}C NMR ($CDCl_3$, 101 MHz) of **21**.

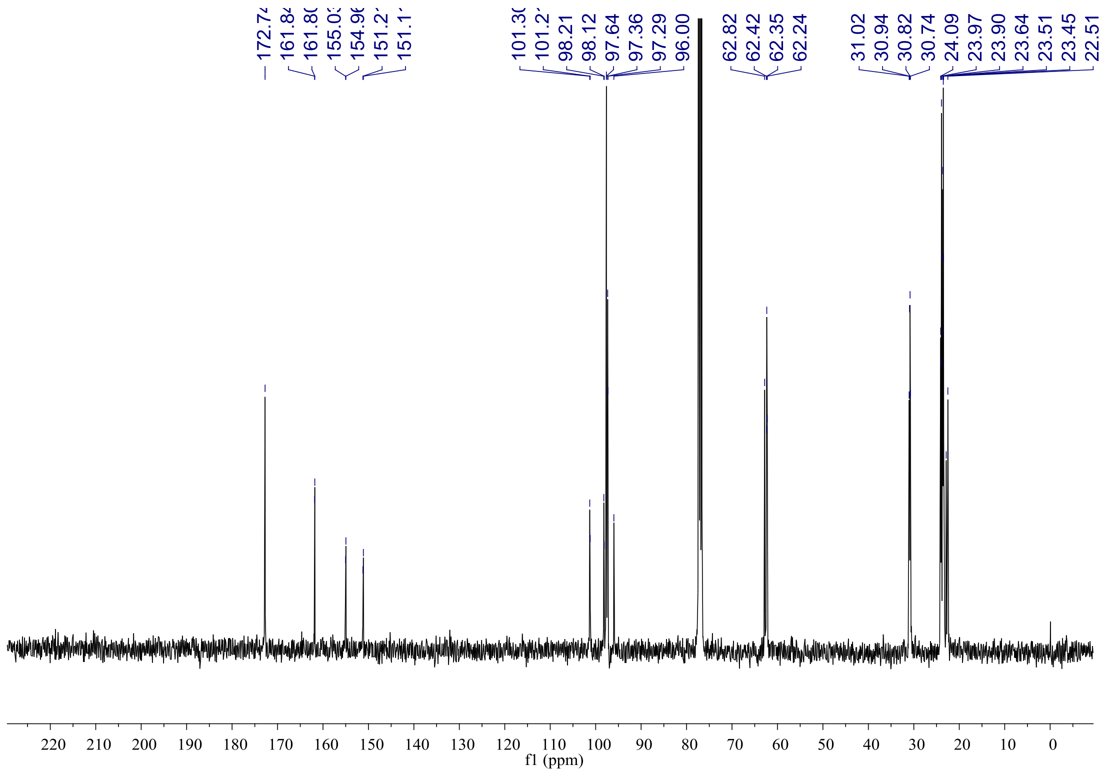

Figure S39. ^{1}H NMR ($CDCl_3$, 400 MHz) of **26**.

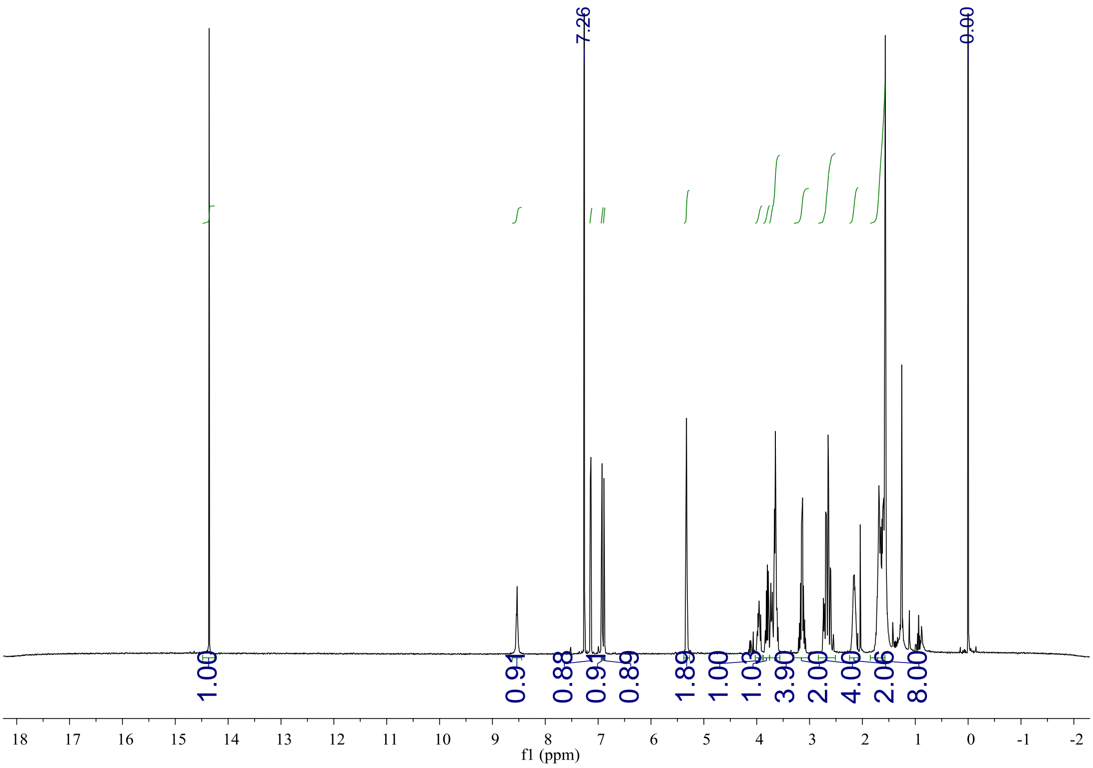

Figure S40. ^{13}C NMR ($CDCl_3$, 101 MHz) of **26**.

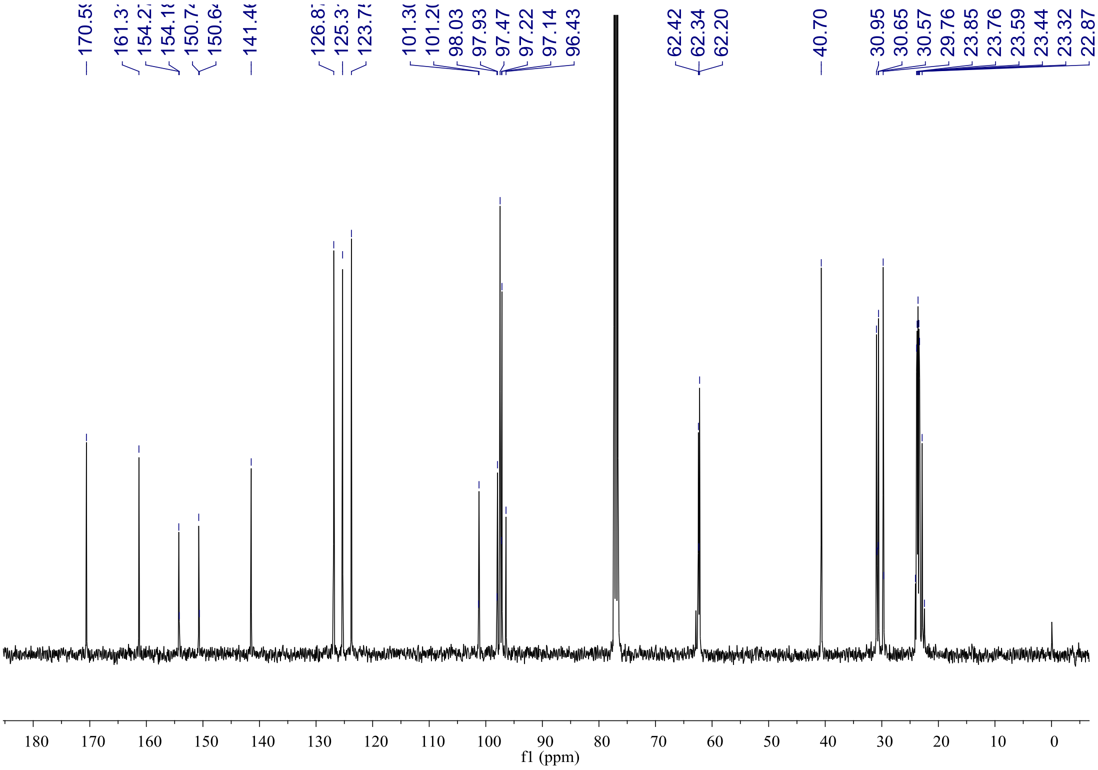

Figure S41. ^{1}H NMR ($CDCl_3$, 400 MHz) of **31**.

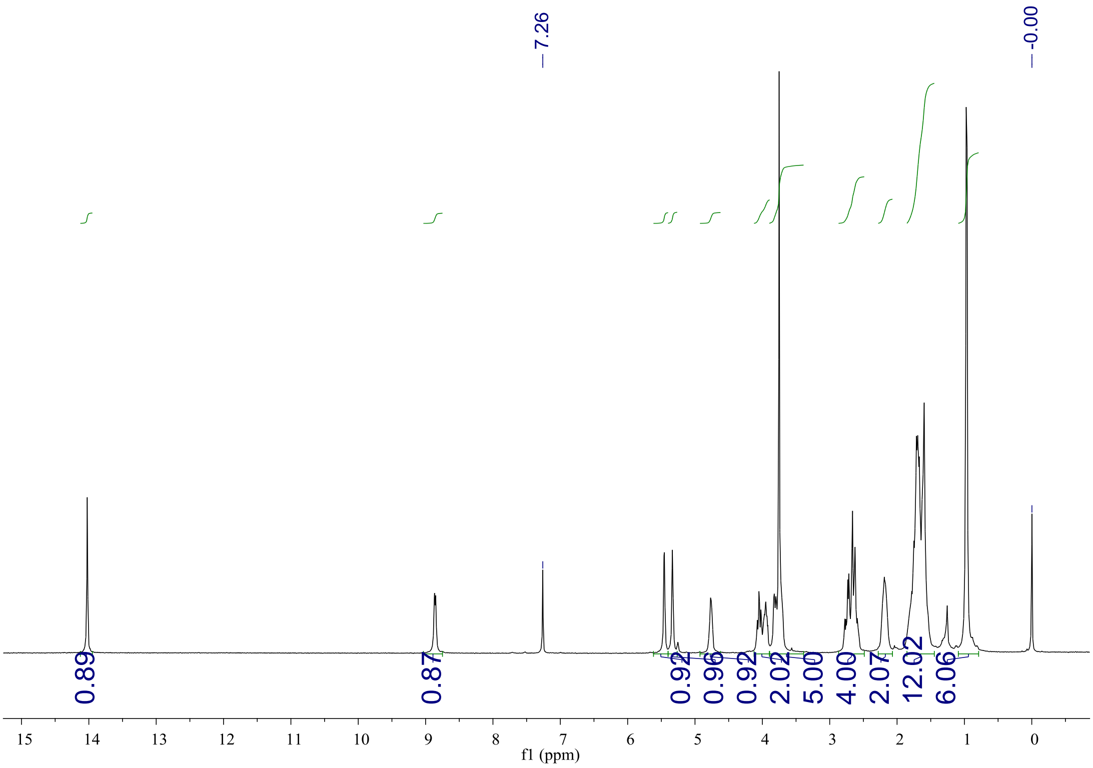

Figure S42. ^{13}C NMR ($CDCl_3$, 101 MHz) of **31**.

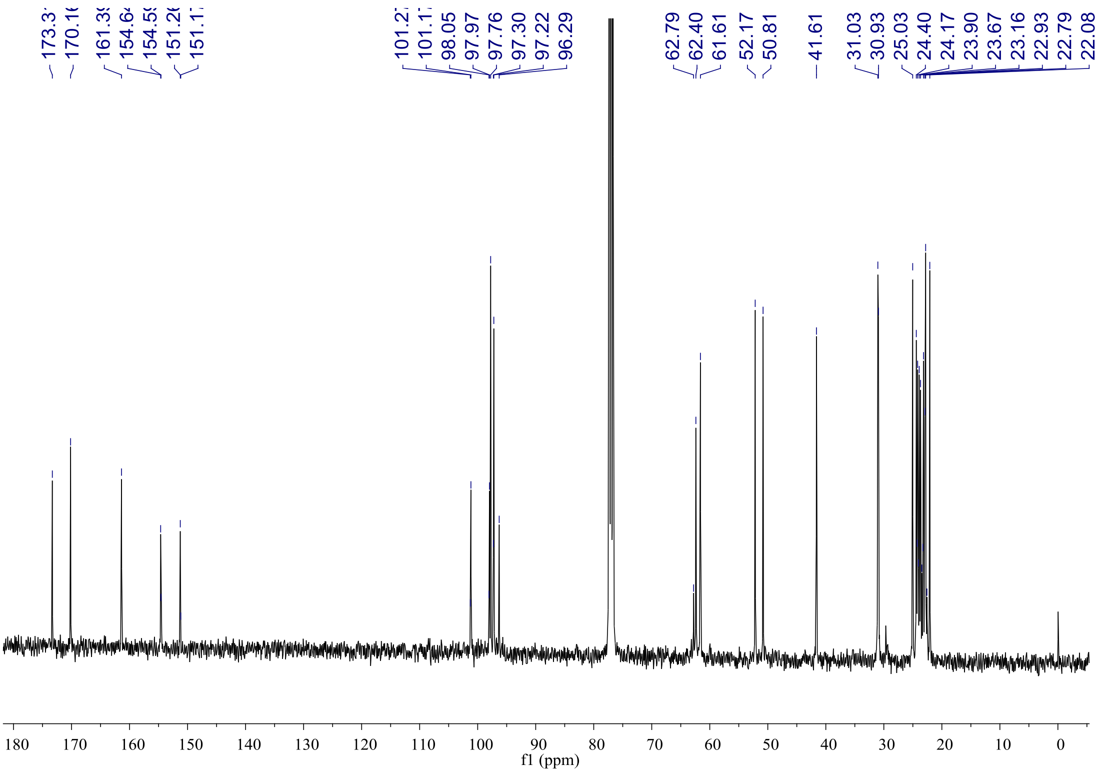

Figure S43. ^{1}H NMR ($CDCl_3$, 400 MHz) of **39**.

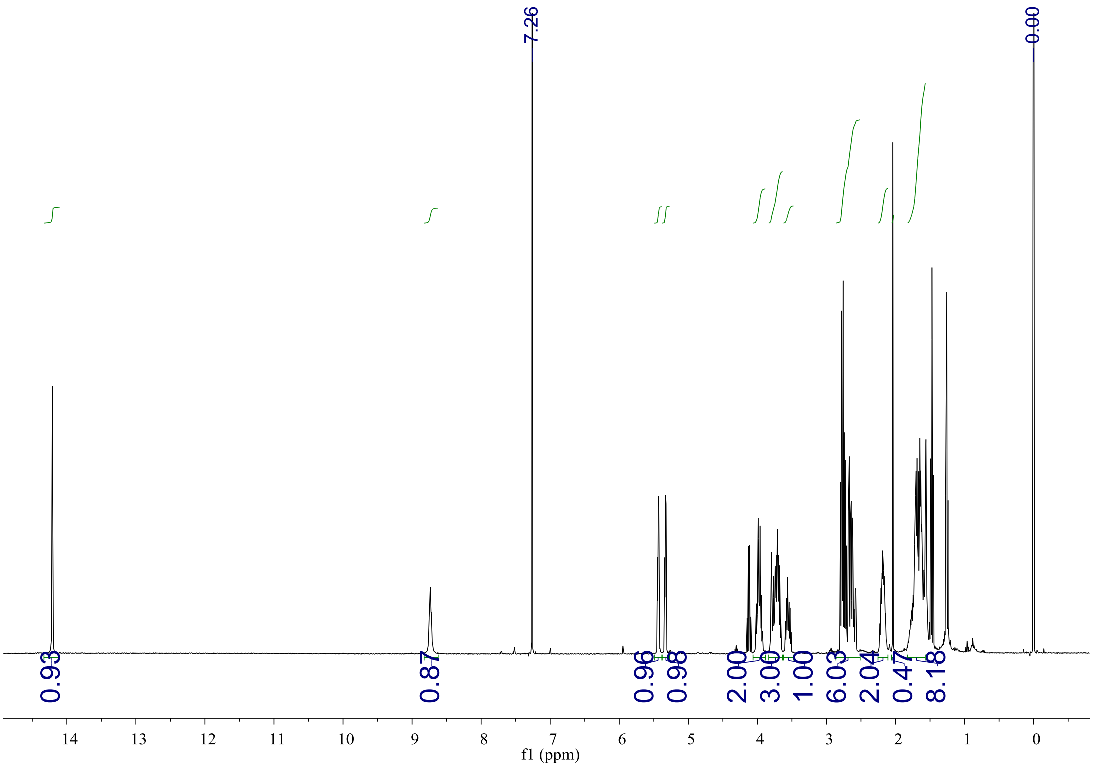

Figure S44. ^{13}C NMR ($CDCl_3$, 101 MHz) of **39**.

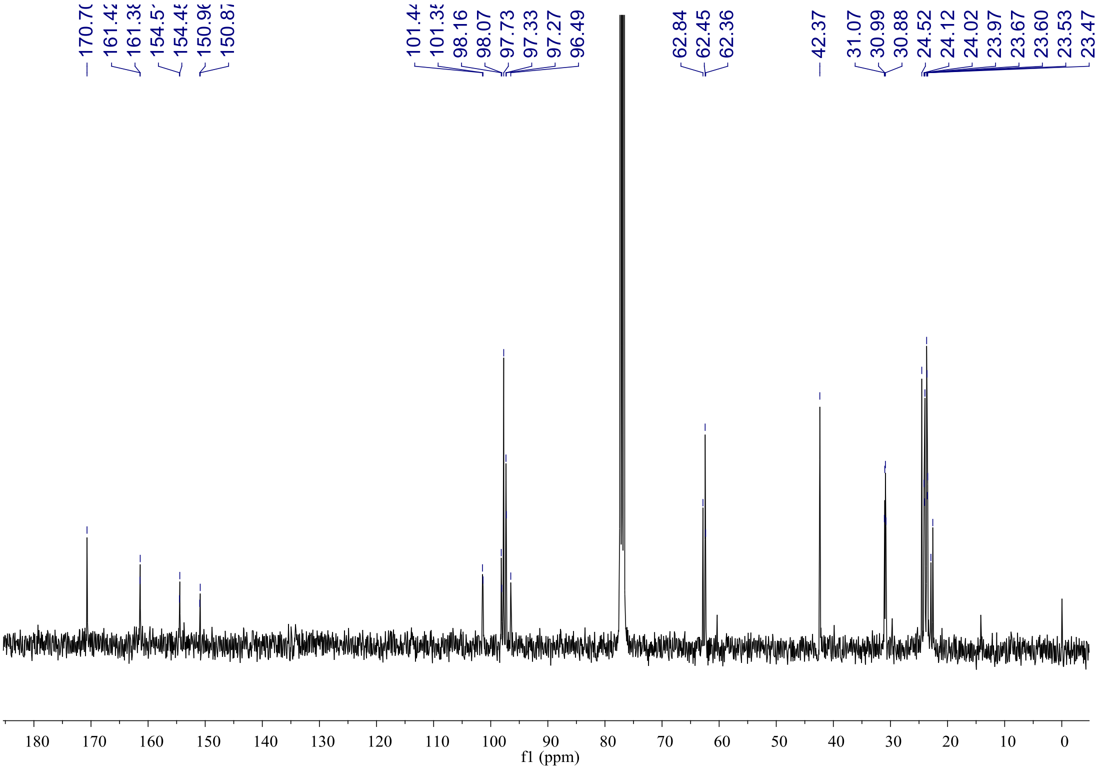

Figure S45. HPLC chromatography and elution conditions of representative compounds. (**A**) The HPLC of compound **1** with elution condition A. (**B**) The HPLC of compound **1** with elution condition B.(**C**) The HPLC of compound **6** with elution condition A (0.1% acetic acid was added). (**D**) The HPLC of compound **6** with elution condition B (0.1% acetic acid was added). (**E**) The HPLC of compound **9** with elution condition A. (**F**) The HPLC of compound **9** with elution condition B. (**G**) The HPLC of compound **23** with elution condition A. (**H**) The HPLC of compound **23** with elution condition B.

A

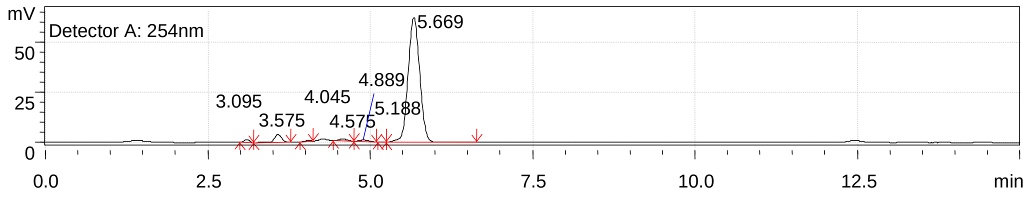

B

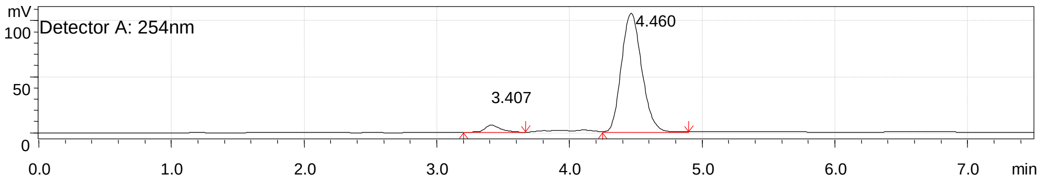

C

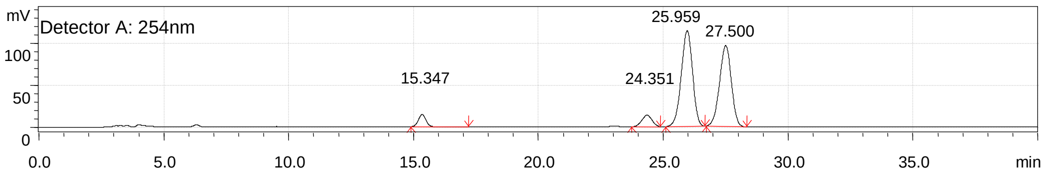

D

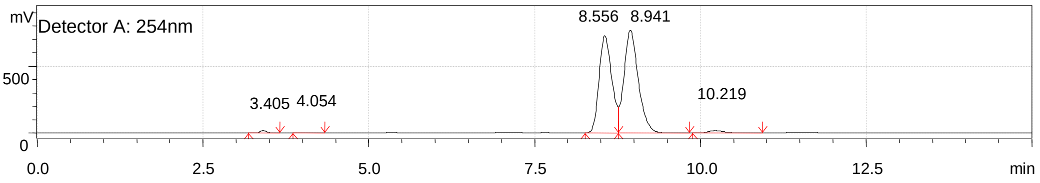

E

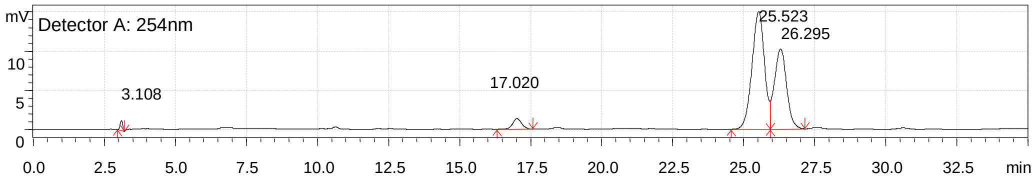

F

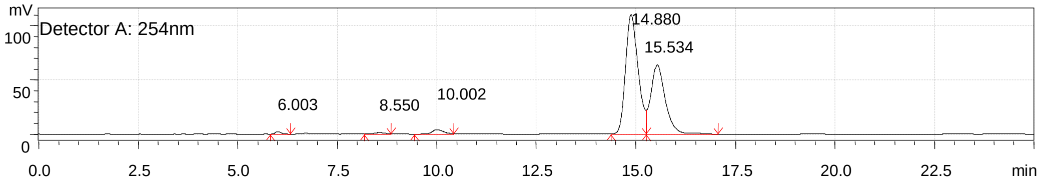

Figure S45. *Cont.*

G

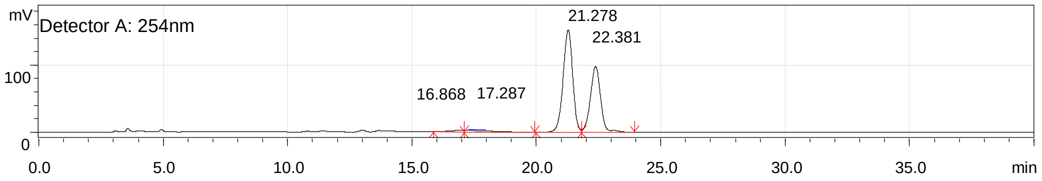

H

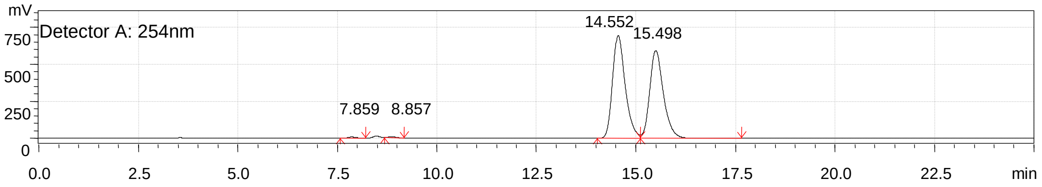

Figure S46. LC-MS of compound **6** under elution condition A (0.1% acetic acid was added).

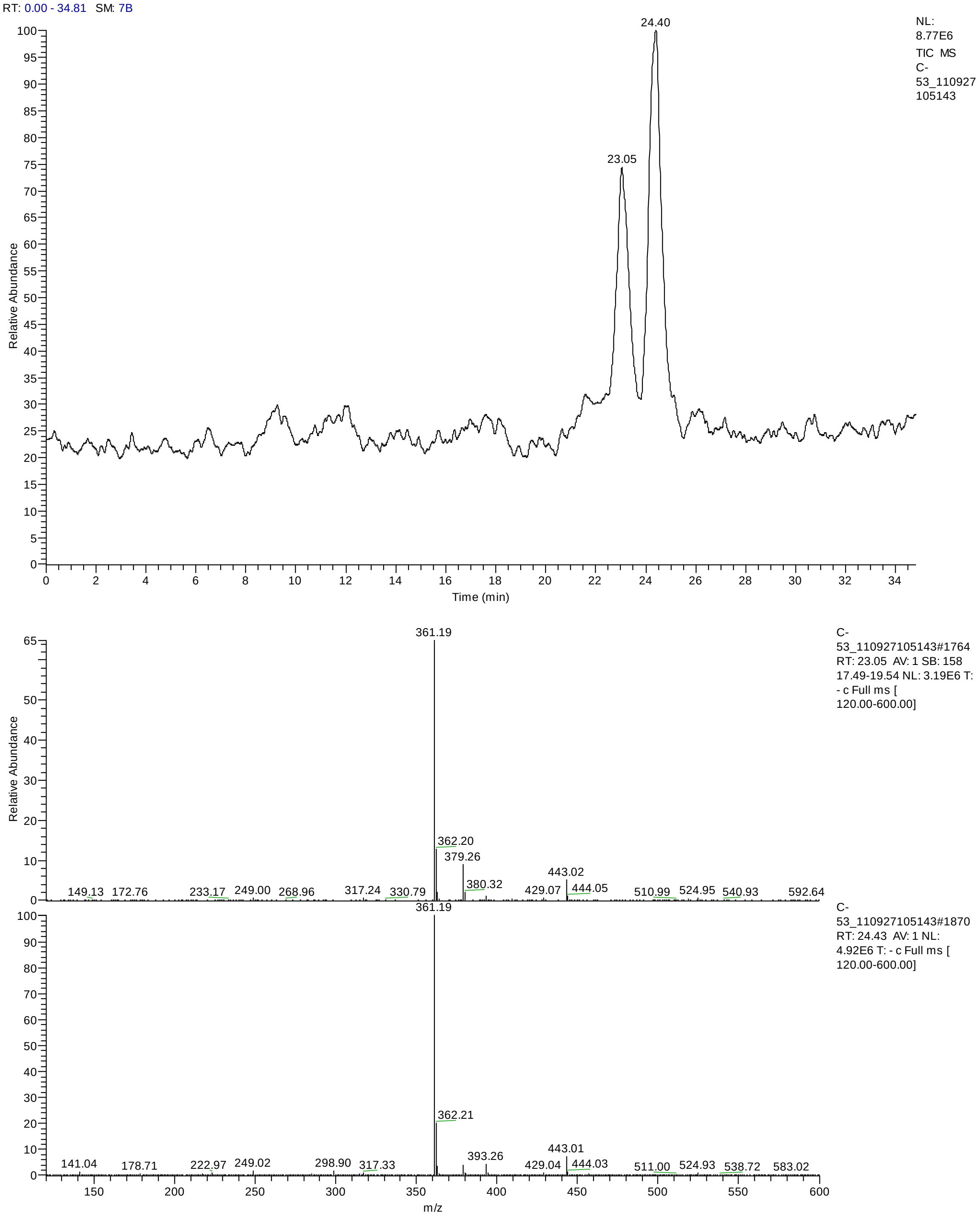

Figure S47. LC-MS of compound **23** using a gradient from 50% to 20% H_2O over 60 min at 1 mL/min.

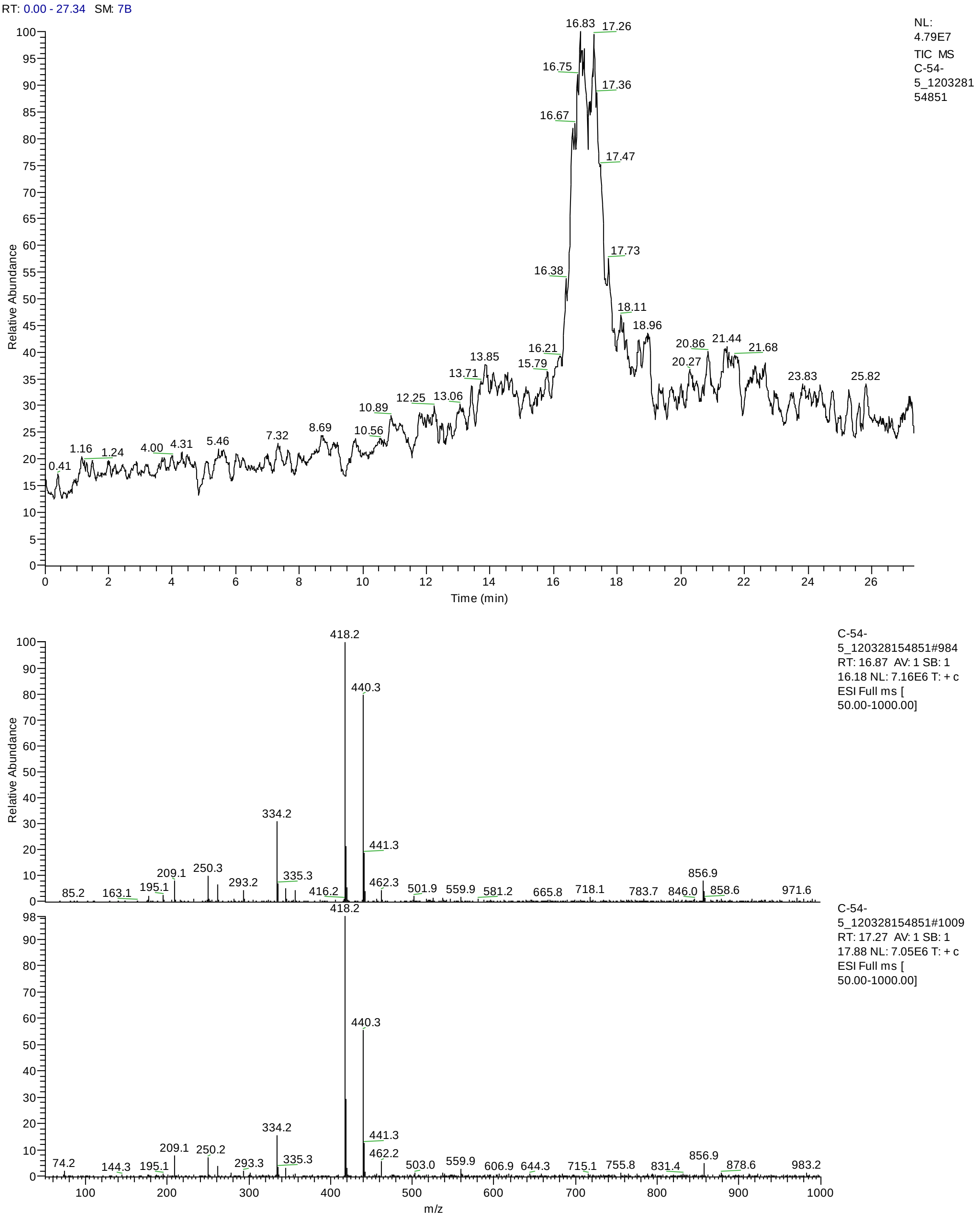

Figure S48. X-ray diffraction data for (±)-**6a**.

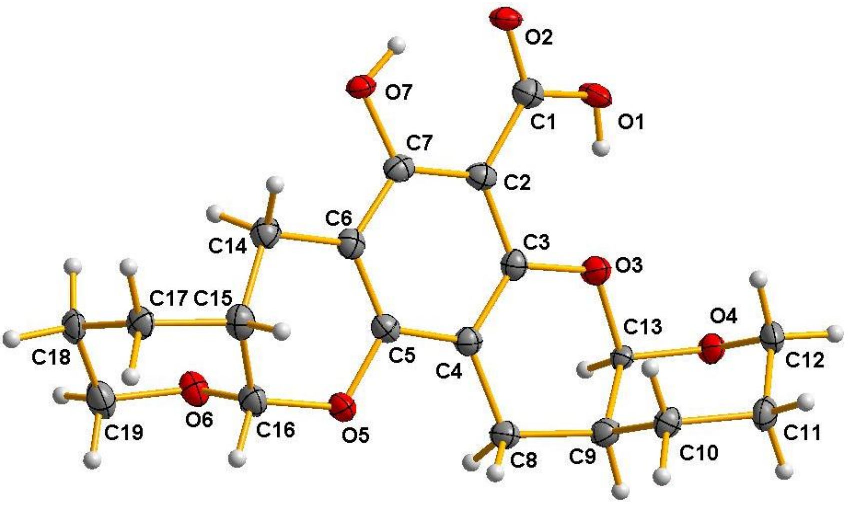

Table S1. NMR spectral data for (±)-**6a** and (±)-**6b** ($CDCl_3$) (δ in ppm, *J* in Hz).

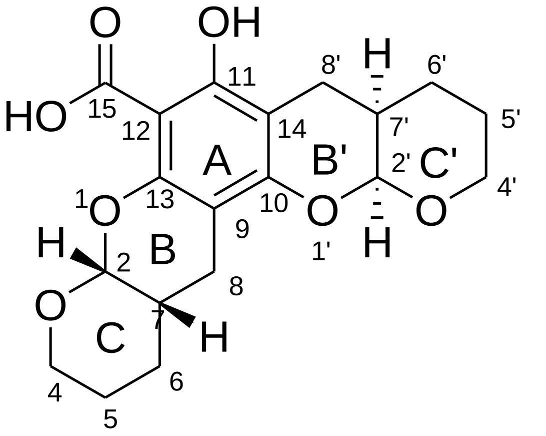

(±)-6a

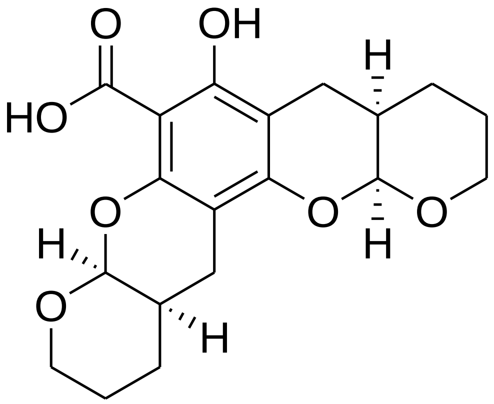

(±)-6b

No.	(±)-6a		(±)-6b	
	^{13}C	^{1}H	^{13}C	^{1}H
15	171.13		171.15	
11	161.24		161.28	
13	156.37		156.45	
10	150.90		151.01	
14	101.92		101.82	
2′	98.94	5.51 (d, *J* = 2.4 Hz)	98.86	5.53 (d, *J* = 2.4 Hz)
9	98.84		98.86	
2	97.54	5.35 (d, *J* = 2.8Hz)	97.50	5.37 (d, *J* = 2.4Hz)
12	94.06		94.04	
4	62.89	3.99 (dd, *J* = 10.4, 4.4 Hz)	62.48	3.96 (dd, *J* = 11.2, 4.0 Hz)
		3.76 (dt, *J* = 11.2, 4.4 Hz)		3.74 (dt, *J* = 11.2, 4.4 Hz)
4′	62.53	3.93 (dd, *J* = 10.4, 3.6 Hz)	62.42	3.91 (dd, *J* = 11.2, 3.6 Hz)
		3.84 (dt, *J* = 11.6, 3.6 Hz)		3.82 (dt, *J* = 11.2, 3.2 Hz)
7′	30.89	2.26–2.21 (m)	30.81	2.27–2.21 (m)
7	30.67	2.21–2.17 (m)	30.76	2.21–2.16 (m)
5′	23.98	1.87–1.54 (m)	23.79	1.80–1.50 (m)
6	23.73	1.87–1.54 (m)	23.79	1.80–1.50 (m)
6′	23.56	1.87–1.54 (m)	23.54	1.80–1.50 (m)
5	23.39	1.87–1.54 (m)	23.40	1.80–1.50 (m)
8′	23.31	2.79 (dd, *J* = 17.2, 6.4 Hz)	23.40	2.76 (dd, *J* = 17.2, 6.4 Hz)
		2.63 (dd, *J* = 17.2, 3.2 Hz)		2.67 (dd, *J* = 17.2, 4.0 Hz)
8	22.39	2.72 (dd, *J* = 17.2, 6.0 Hz)	22.76	2.71 (dd, *J* = 17.2, 3.6 Hz)
		2.65 (dd, *J* = 16.8, 3.2 Hz)		2.63 (dd, *J* = 16.8, 4.0 Hz)
OH		12.49 (s)		12.48 (s)
COOH		11.33 (s)		11.32 (s)

Table S2. Purity and retention times of all tested compounds.

	Elution condition A		Elution condition B	
**Compound**	**Purity, %**	**Retention Time/min**	**Purity, %**	**Retention Time/min**
1	95.70	5.669	95.04	4.460
2	99.28	10.063 *	98.69	7.343 *
3	99.06	7.099	99.16	5.818
4	97.93	13.656, 14.251	98.46	9.504, 10.003
5	97.64	6.968	99.61	5.688
6	95.7	26.959, 27.500 *	97.50	8.556, 8.941 *
7	98.91	12.717, 13.345	98.86	6.258, 6.478
9	98.63	10.466, 10.715	98.21	7.829, 8.096
10	98.71	6.689	96.75	6.909, 7.050
11	96.12	25.523, 26.295	96.59	14.880, 15.534
12	96.49	15.319, 15.741	98.93	9.915, 10.289
13	96.14	19.473, 20.129	96.44	13.215, 13.822
14	95.12	37.503, 38.887	95.48	22.721, 23.901
15	95.60	14.066, 14.448	98.56	9.360, 9.700
16	95.24	6.897	95.39	5.059
17	95.91	45.311, 49.089	96.61	45.612, 48.194
18	98.46	16.108	96.00	8.302
19	99.38	32.389	95.28	17.504
20	97.77	38.718, 39.817	99.02	17.980, 18.758
21	98.14	6.870, 7.066	99.91	5.273, 5.426
22	99.35	6.910, 7.110	96.80	6.835, 7.098
23	96.05	21.278, 22.381	99.29	14.552, 15.498
24	95.81	12.766	99.48	8.380, 8.848
25	96.44	13.215, 13.822	96.85	4.413
26	99.01	21.773, 22.822	98.92	12.376, 13.039
27	99.90	31.753, 33.393	99.61	15.318, 16.174
28	97.95	4.139 *	98.10	3.902 *
29	98.07	3.269 *	99.58	6.437, 6.665 *
30	95.39	14.248	95.53	3.132
31	98.31	26.740, 28.358	99.62	14.890, 16.312
32	95.64	3.477 *	98.31	3.235 *
33	95.02	5.710 *	95.08	17.355
34	99.94	5.052 *	99.58	4.000 *
35	98.99	6.686	99.63	4.664
36	96.24	10.840	96.39	6.506
37	95.68	4.212 *	96.21	3.915 *
38	95.32	25.539 *	95.48	13.575 *
39	98.51	12.571, 13.101	98.47	8.578, 8.979

The purities of all test compounds are at least 95% as determined by an HPLC equipped with a UV detector at 254 nm and a C18 column (5 µm, 4.6 × 250 mm) at 25 °C. All of the purchased HPLC grade solvents were ultrasonically degassed for 30 min before use. Purity analysis using the elution conditions A and B was performed. Elution condition A included a water:methanol ratio of 20:80 (v/v) and a flow rate = 1 mL/min. Elution condition B included a water:acetonitrile ratio of 20:80 (v/v) and a flow rate = 1 mL/min. Table S2 summarizes the purities and retention times for all of the test compounds.* 0.1% acetic acid was added.

Table S3. Crystal data and structure refinement for (±)-**6a**.

**Crystal Data and Structure Refinement for (±)-6a**		
Identification code	q1	
Empirical formula	C19 H22 O7	
Formula weight	362.37	
Temperature	150(2) K	
Wavelength	1.54178 Å	
Crystal system, space group	Monoclinic, C2/c	
Unit cell dimensions	a = 25.4092(3) Å	alpha = 90 °
	b = 12.9938(2) Å	beta = 111.4390(10) °
	c = 10.67030(10) Å	gamma = 90 °
Volume	3279.17(7) Å^3	
Z, Calculated density	8, 1.468 Mg/m^3	
Absorption coefficient	0.939 mm^−1	
F(000)	1536	
Crystal size	0.42 × 0.40 × 0.37 mm	
Theta range for data collection	3.88 to 66.88 °	
Limiting indices	$-29 \leq h \leq 30, -15 \leq k \leq 15, -12 \leq l \leq 12$	
Reflections collected/unique	22,987/2914 (R(int) = 0.0279)	
Completeness to theta = 66.88	99.7%	
Absorption correction	Semi-empirical from equivalents	
Maximum and minimum transmission	0.7225 and 0.6937	
Refinement method	Full-matrix least-squares on F^2	
Data/restraints/parameters	2914/0/238	
Goodness-of-fit on F^2	1.073	
Final R indices (I > 2 sigma(I))	R1 = 0.0322, wR2 = 0.0791	
R indices (all data)	R1 = 0.0353, wR2 = 0.0813	
Extinction coefficient	0.00116(7)	
Largest diffrction peak and hole	0.216 and −0.186 e. Å^−3	

Table S4. Atomic coordinates (×10^4) and equivalent isotropic displacement parameters ($Å^2$ × 10^3) for (±)-**6a**.

	**X**	**Y**	**Z**	**U(eq)** [1]
C(1)	3614(1)	2903(1)	3197(1)	23(1)
C(2)	3118(1)	2282(1)	2412(1)	20(1)
C(3)	3100(1)	1600(1)	1358(1)	19(1)
C(4)	2626(1)	1025(1)	660(1)	19(1)
C(5)	2154(1)	1138(1)	1034(1)	19(1)
C(6)	2149(1)	1754(1)	2090(1)	20(1)
C(7)	2632(1)	2337(1)	2753(1)	20(1)
C(8)	2617(1)	293(1)	−447(1)	22(1)
C(9)	3213(1)	44(1)	−386(1)	21(1)
C(10)	3522(1)	−754(1)	676(1)	29(1)
C(11)	4125(1)	−875(1)	717(2)	39(1)
C(12)	4416(1)	161(1)	937(2)	38(1)
C(13)	3551(1)	1029(1)	−167(1)	23(1)
C(14)	1649(1)	1777(1)	2535(1)	22(1)
C(15)	1252(1)	875(1)	1945(1)	24(1)
C(16)	1179(1)	730(1)	482(1)	23(1)
C(17)	663(1)	1038(1)	2015(2)	30(1)
C(18)	334(1)	1884(1)	1051(2)	32(1)
C(19)	355(1)	1732(1)	−342(2)	31(1)
O(1)	4042(1)	3031(1)	2793(1)	36(1)
O(2)	3647(1)	3334(1)	4250(1)	30(1)
O(3)	3587(1)	1524(1)	1089(1)	24(1)
O(4)	4107(1)	895(1)	−76(1)	31(1)
O(5)	1694(1)	565(1)	277(1)	24(1)
O(6)	930(1)	1622(1)	−268(1)	25(1)
O(7)	2604(1)	2976(1)	3724(1)	27(1)

[1] U(eq) is defined as one-third of the trace of the orthogonalized Uij tensor.

Table S5. Bond lengths (Å) and angles (deg.) for (±)-**6a**.

Bond	Lengths (Angles)	Bond	Lengths (Angles)
C(1)-O(2)	1.2302(16)	C(4)-C(8)-H(8B)	109.3
C(1)-O(1)	1.3182(16)	C(9)-C(8)-H(8B)	109.3
C(1)-C(2)	1.4725(17)	H(8A)-C(8)-H(8B)	108
C(2)-C(7)	1.4099(18)	C(13)-C(9)-C(10)	110.61(11)
C(2)-C(3)	1.4190(17)	C(13)-C(9)-C(8)	109.05(10)
C(3)-O(3)	1.3708(15)	C(10)-C(9)-C(8)	114.12(11)
C(3)-C(4)	1.3815(17)	C(13)-C(9)-H(9)	107.6
C(4)-C(5)	1.4028(17)	C(10)-C(9)-H(9)	107.6
C(4)-C(8)	1.5100(17)	C(8)-C(9)-H(9)	107.6
C(5)-O(5)	1.3721(15)	C(11)-C(10)-C(9)	109.27(12)
C(5)-C(6)	1.3871(18)	C(11)-C(10)-H(10A)	109.8
C(6)-C(7)	1.3964(18)	C(9)-C(10)-H(10A)	109.8
C(6)-C(14)	1.5087(17)	C(11)-C(10)-H(10B)	109.8
C(7)-O(7)	1.3503(15)	C(9)-C(10)-H(10B)	109.8
C(8)-C(9)	1.5260(17)	H(10A)-C(10)-H(10B)	108.3
C(8)-H(8A)	0.99	C(12)-C(11)-C(10)	109.98(12)
C(8)-H(8B)	0.99	C(12)-C(11)-H(11A)	109.7
C(9)-C(13)	1.5116(18)	C(10)-C(11)-H(11A)	109.7
C(9)-C(10)	1.5254(18)	C(12)-C(11)-H(11B)	109.7
C(9)-H(9)	1	C(10)-C(11)-H(11B)	109.7
C(10)-C(11)	1.525(2)	H(11A)-C(11)-H(11B)	108.2
C(10)-H(10A)	0.99	O(4)-C(12)-C(11)	111.86(12)
C(10)-H(10B)	0.99	O(4)-C(12)-H(12A)	109.2
C(11)-C(12)	1.512(2)	C(11)-C(12)-H(12A)	109.2
C(11)-H(11A)	0.99	O(4)-C(12)-H(12B)	109.2
C(11)-H(11B)	0.99	C(11)-C(12)-H(12B)	109.2
C(12)-O(4)	1.4399(19)	H(12A)-C(12)-H(12B)	107.9
C(12)-H(12A)	0.99	O(4)-C(13)-O(3)	105.74(10)
C(12)-H(12B)	0.99	O(4)-C(13)-C(9)	114.19(11)
C(13)-O(4)	1.3908(15)	O(3)-C(13)-C(9)	110.85(10)
C(13)-O(3)	1.4587(15)	O(4)-C(13)-H(13)	108.6
C(13)-H(13)	1	O(3)-C(13)-H(13)	108.6
C(14)-C(15)	1.5248(18)	C(9)-C(13)-H(13)	108.6
C(14)-H(14A)	0.99	C(6)-C(14)-C(15)	111.19(10)
C(14)-H(14B)	0.99	C(6)-C(14)-H(14A)	109.4
C(15)-C(16)	1.5158(18)	C(15)-C(14)-H(14A)	109.4
C(15)-C(17)	1.5384(18)	C(6)-C(14)-H(14B)	109.4
C(15)-H(15)	1	C(15)-C(14)-H(14B)	109.4
C(16)-O(6)	1.4188(16)	H(14A)-C(14)-H(14B)	108
C(16)-O(5)	1.4198(15)	C(16)-C(15)-C(14)	109.47(10)
C(16)-H(16)	1	C(16)-C(15)-C(17)	108.18(11)
C(17)-C(18)	1.529(2)	C(14)-C(15)-C(17)	112.54(11)
C(17)-H(17A)	0.99	C(16)-C(15)-H(15)	108.9
C(17)-H(17B)	0.99	C(14)-C(15)-H(15)	108.9
C(18)-C(19)	1.519(2)	C(17)-C(15)-H(15)	108.9

Table S5. *Cont.*

C(18)-H(18A)	0.99	O(6)-C(16)-O(5)	106.41(10)
C(18)-H(18B)	0.99	O(6)-C(16)-C(15)	109.91(10)
C(19)-O(6)	1.4411(16)	O(5)-C(16)-C(15)	113.92(10)
C(19)-H(19A)	0.99	O(6)-C(16)-H(16)	108.8
C(19)-H(19B)	0.99	O(5)-C(16)-H(16)	108.8
O(1)-H(1)	0.84	C(15)-C(16)-H(16)	108.8
O(7)-H(7)	0.84	C(18)-C(17)-C(15)	111.68(11)
O(2)-C(1)-O(1)	116.87(11)	C(18)-C(17)-H(17A)	109.3
O(2)-C(1)-C(2)	121.83(12)	C(15)-C(17)-H(17A)	109.3
O(1)-C(1)-C(2)	121.30(11)	C(18)-C(17)-H(17B)	109.3
C(7)-C(2)-C(3)	117.31(11)	C(15)-C(17)-H(17B)	109.3
C(7)-C(2)-C(1)	118.23(11)	H(17A)-C(17)-H(17B)	107.9
C(3)-C(2)-C(1)	124.43(11)	C(19)-C(18)-C(17)	110.98(12)
O(3)-C(3)-C(4)	121.50(11)	C(19)-C(18)-H(18A)	109.4
O(3)-C(3)-C(2)	116.20(11)	C(17)-C(18)-H(18A)	109.4
C(4)-C(3)-C(2)	122.28(11)	C(19)-C(18)-H(18B)	109.4
C(3)-C(4)-C(5)	117.22(11)	C(17)-C(18)-H(18B)	109.4
C(3)-C(4)-C(8)	121.60(11)	H(18A)-C(18)-H(18B)	108
C(5)-C(4)-C(8)	121.18(11)	O(6)-C(19)-C(18)	110.92(11)
O(5)-C(5)-C(6)	122.07(11)	O(6)-C(19)-H(19A)	109.5
O(5)-C(5)-C(4)	114.17(11)	C(18)-C(19)-H(19A)	109.5
C(6)-C(5)-C(4)	123.74(11)	O(6)-C(19)-H(19B)	109.5
C(5)-C(6)-C(7)	117.11(11)	C(18)-C(19)-H(19B)	109.5
C(5)-C(6)-C(14)	121.82(11)	H(19A)-C(19)-H(19B)	108
C(7)-C(6)-C(14)	121.06(11)	C(1)-O(1)-H(1)	109.5
O(7)-C(7)-C(6)	116.03(11)	C(3)-O(3)-C(13)	117.84(9)
O(7)-C(7)-C(2)	121.72(11)	C(13)-O(4)-C(12)	113.32(10)
C(6)-C(7)-C(2)	122.23(11)	C(5)-O(5)-C(16)	117.60(10)
C(4)-C(8)-C(9)	111.63(10)	C(16)-O(6)-C(19)	110.29(10)
C(4)-C(8)-H(8A)	109.3	C(7)-O(7)-H(7)	109.5
C(9)-C(8)-H(8A)	109.3		

Table S6. Anisotropic displacement parameters (Å^2 × 10^3) for (±)-**6a** [1].

	**U11**	**U22**	**U33**	**U23**	**U13**	**U12**
C(1)	23(1)	23(1)	22(1)	−1(1)	6(1)	−1(1)
C(2)	21(1)	20(1)	17(1)	0(1)	5(1)	−1(1)
C(3)	19(1)	19(1)	21(1)	2(1)	8(1)	1(1)
C(4)	20(1)	19(1)	19(1)	0(1)	7(1)	0(1)
C(5)	17(1)	20(1)	20(1)	1(1)	5(1)	−1(1)
C(6)	20(1)	21(1)	18(1)	2(1)	7(1)	2(1)
C(7)	24(1)	19(1)	15(1)	1(1)	6(1)	2(1)
C(8)	21(1)	23(1)	22(1)	−4(1)	8(1)	−3(1)
C(9)	22(1)	22(1)	21(1)	−3(1)	10(1)	−1(1)
C(10)	32(1)	25(1)	32(1)	3(1)	13(1)	4(1)
C(11)	33(1)	39(1)	43(1)	4(1)	12(1)	13(1)
C(12)	20(1)	54(1)	38(1)	−9(1)	8(1)	5(1)
C(13)	23(1)	26(1)	23(1)	−2(1)	12(1)	−1(1)
C(14)	22(1)	25(1)	21(1)	0(1)	9(1)	2(1)
C(15)	22(1)	23(1)	26(1)	3(1)	10(1)	1(1)
C(16)	17(1)	24(1)	29(1)	−2(1)	9(1)	−2(1)
C(17)	26(1)	33(1)	36(1)	0(1)	18(1)	−4(1)
C(18)	20(1)	36(1)	42(1)	−1(1)	15(1)	1(1)
C(19)	18(1)	35(1)	37(1)	1(1)	7(1)	0(1)
O(1)	25(1)	54(1)	29(1)	−16(1)	11(1)	−16(1)
O(2)	32(1)	33(1)	25(1)	−11(1)	9(1)	−8(1)
O(3)	20(1)	27(1)	28(1)	−8(1)	12(1)	−5(1)
O(4)	25(1)	36(1)	38(1)	−7(1)	19(1)	−5(1)
O(5)	16(1)	29(1)	28(1)	−9(1)	8(1)	−4(1)
O(6)	18(1)	30(1)	25(1)	2(1)	7(1)	0(1)
O(7)	29(1)	31(1)	23(1)	−10(1)	11(1)	−4(1)

[1] The anisotropic displacement factor exponent takes the form: −2 pi^2 [h^2 a*^2 U11 + ... + 2 h k a* b* U12].

Table S7. Hydrogen coordinates (×10^4) and isotropic displacement parameters (Å^2 × 10^3) for (±)-**6a**.

	**X**	**Y**	**Z**	**U(eq)**
H(8A)	2398	605	−1331	26
H(8B)	2425	−352	−364	26
H(9)	3180	−238	−1284	26
H(10A)	3528	−531	1569	35
H(10B)	3322	−1422	454	35
H(11A)	4337	−1348	1456	47
H(11B)	4118	−1175	−141	47
H(12A)	4453	427	1835	45
H(12B)	4800	78	920	45
H(13)	3358	1509	−930	27
H(14A)	1441	2431	2241	27
H(14B)	1785	1748	3530	27
H(15)	1422	237	2456	28
H(16)	924	129	110	28
H(17A)	447	386	1784	36
H(17B)	706	1226	2946	36
H(18A)	−65	1876	987	38
H(18B)	497	2563	1407	38
H(19A)	137	1110	−760	37
H(19B)	179	2331	−916	37
H(1)	3962	2770	2025	53
H(7)	2921	3254	4113	41

Table S8. Torsion angles (deg.) for (±)-**6a**.

Bond	Torsion Angles
O(2)-C(1)-C(2)-C(7)	11.36(19)
O(1)-C(1)-C(2)-C(7)	−167.91(12)
O(2)-C(1)-C(2)-C(3)	−166.46(12)
O(1)-C(1)-C(2)-C(3)	14.3(2)
C(7)-C(2)-C(3)-O(3)	−177.07(10)
C(1)-C(2)-C(3)-O(3)	0.78(18)
C(7)-C(2)-C(3)-C(4)	1.43(18)
C(1)-C(2)-C(3)-C(4)	179.27(12)
O(3)-C(3)-C(4)-C(5)	178.41(11)
C(2)-C(3)-C(4)-C(5)	0.00(18)
O(3)-C(3)-C(4)-C(8)	−0.80(18)
C(2)-C(3)-C(4)-C(8)	−179.21(11)
C(3)-C(4)-C(5)-O(5)	178.37(10)
C(8)-C(4)-C(5)-O(5)	−2.41(17)
C(3)-C(4)-C(5)-C(6)	−2.90(18)
C(8)-C(4)-C(5)-C(6)	176.32(12)
O(5)-C(5)-C(6)-C(7)	−177.27(11)
C(4)-C(5)-C(6)-C(7)	4.09(18)
O(5)-C(5)-C(6)-C(14)	4.27(18)
C(4)-C(5)-C(6)-C(14)	−174.36(11)
C(5)-C(6)-C(7)-O(7)	176.23(11)
C(14)-C(6)-C(7)-O(7)	−5.30(17)
C(5)-C(6)-C(7)-C(2)	−2.48(18)
C(14)-C(6)-C(7)-C(2)	175.99(11)
C(3)-C(2)-C(7)-O(7)	−178.78(11)
C(1)-C(2)-C(7)-O(7)	3.24(18)
C(3)-C(2)-C(7)-C(6)	−0.14(18)
C(1)-C(2)-C(7)-C(6)	−178.12(11)
C(3)-C(4)-C(8)-C(9)	17.02(17)
C(5)-C(4)-C(8)-C(9)	−162.16(11)
C(4)-C(8)-C(9)-C(13)	−45.13(14)
C(4)-C(8)-C(9)-C(10)	79.11(14)
C(13)-C(9)-C(10)-C(11)	−52.16(15)
C(8)-C(9)-C(10)-C(11)	−175.55(11)
C(9)-C(10)-C(11)-C(12)	54.60(16)
C(10)-C(11)-C(12)-O(4)	−56.39(17)
C(10)-C(9)-C(13)-O(4)	52.91(14)
C(8)-C(9)-C(13)-O(4)	179.19(10)
C(10)-C(9)-C(13)-O(3)	−66.39(13)
C(8)-C(9)-C(13)-O(3)	59.89(13)
C(5)-C(6)-C(14)-C(15)	14.52(16)
C(7)-C(6)-C(14)-C(15)	−163.88(11)
C(6)-C(14)-C(15)-C(16)	−42.59(14)
C(6)-C(14)-C(15)-C(17)	−162.92(11)
C(14)-C(15)-C(16)-O(6)	−61.93(13)

Table S8. *Cont.*

C(17)-C(15)-C(16)-O(6)	61.03(13)
C(14)-C(15)-C(16)-O(5)	57.36(14)
C(17)-C(15)-C(16)-O(5)	−179.68(10)
C(16)-C(15)-C(17)-C(18)	−51.52(15)
C(14)-C(15)-C(17)-C(18)	69.56(15)
C(15)-C(17)-C(18)-C(19)	48.09(16)
C(17)-C(18)-C(19)-O(6)	−52.49(15)
C(4)-C(3)-O(3)-C(13)	15.45(17)
C(2)-C(3)-O(3)-C(13)	−166.04(11)
O(4)-C(13)-O(3)-C(3)	−169.92(10)
C(9)-C(13)-O(3)-C(3)	−45.66(14)
O(3)-C(13)-O(4)-C(12)	67.44(13)
C(9)-C(13)-O(4)-C(12)	−54.69(15)
C(11)-C(12)-O(4)-C(13)	56.34(15)
C(6)-C(5)-O(5)-C(16)	8.67(17)
C(4)-C(5)-O(5)-C(16)	−172.58(10)
O(6)-C(16)-O(5)-C(5)	80.99(13)
C(15)-C(16)-O(5)-C(5)	−40.27(15)
O(5)-C(16)-O(6)-C(19)	168.52(10)
C(15)-C(16)-O(6)-C(19)	−67.69(13)
C(18)-C(19)-O(6)-C(16)	62.76(14)

Table S9. Hydrogen bonds for (±)-**6a** (Å and deg.) [1].

D-H···A	d(D-H)	d(H···A)	d(D···A)	∠ (DHA)
O(1)-H(1)···O(3)	0.84	1.96	2.6345(13)	136.9
O(1)-H(1)···O(6)#1	0.84	2.14	2.7592(13)	129.9
O(7)-H(7)···O(2)	0.84	1.8	2.5416(13)	146.5

[1] Symmetry transformations used to generate equivalent atoms: #1 −x + 1/2, −y + 1/2, −z.
